# Skin permeability prediction with MD simulation sampling spatial and alchemical reaction coordinates

**DOI:** 10.1016/j.bpj.2022.09.009

**Published:** 2022-09-13

**Authors:** Magnus Lundborg, Christian Wennberg, Jack Lidmar, Berk Hess, Erik Lindahl, Lars Norlén

**Affiliations:** 1 ERCO Pharma AB, Science for Life Laboratory, Solna, Sweden; 2 Department of Physics, KTH Royal Institute of Technology, Stockholm, Sweden; 3 Department of Applied Physics, Science for Life Laboratory, KTH Royal Institute of Technology, Solna, Sweden; 4 Department of Biophysics and Biochemistry, Science for Life Laboratory, Stockholm University, Solna, Sweden; 5 Department of Physics, Swedish e-Science Research Center, KTH Royal Institute of Technology, Stockholm, Sweden; 6 Department of Cell and Molecular Biology (CMB), Karolinska Institutet, Stockholm, Sweden; 7 Dermatology Clinic, Karolinska University Hospital, Stockholm, Sweden

## Abstract

A molecular-level understanding of skin permeation may rationalize and streamline product development, and improve quality and control, of transdermal and topical drug delivery systems. It may also facilitate toxicity and safety assessment of cosmetics and skin care products. Here, we present new molecular dynamics simulation approaches that make it possible to efficiently sample the free energy and local diffusion coefficient across the skin’s barrier structure to predict skin permeability and the effects of chemical penetration enhancers. In particular, we introduce a new approach to use two-dimensional reaction coordinates in the accelerated weight histogram method, where we combine sampling along spatial coordinates with an alchemical perturbation virtual coordinate. We present predicted properties for 20 permeants, and demonstrate how our approach improves correlation with ex vivo/in vitro skin permeation data. For the compounds included in this study, the obtained log K_Pexp-calc_ mean square difference was 0.9 cm^2^ h^−2^.

## Significance

Percutaneous drug administration allows for noninvasive, pain-free, continuous drug delivery with reduced side effects and increased patient compliance compared with per oral or intravenous drug administration.

Permeability coefficients can be predicted using molecular dynamics simulations in silico with an atomistic model of the skin’s barrier structure validated by cryo-EM in near-native skin. This can assist the interpretation of ex vivo/in vitro skin permeability data. The possibility to identify the locations of the most significant permeation barriers, as well as where permeation enhancers partition into the barrier structure, can help when designing transdermal, and topical, drug delivery systems. It may also be used for toxicity predictions of cosmetics or hazards in the work environment.

Here, we present the first use of the accelerated weight histogram method with a reaction coordinate combining a spatial dimension with an alchemical perturbation dimension. This yields a more efficient sampling of the free energy landscape and local diffusion coefficient of drugs passing through the skin’s barrier structure. The two-dimensional sampling method can be applied to other molecular dynamics simulation systems with long correlation times.

## Introduction

Transdermal and topical drug delivery allows for noninvasive, pain-free, continuous drug administration with reduced side effects and increased patient compliance compared with per oral or intravenous drug delivery.

Assessment of skin permeability is important for the design of percutaneous drug delivery systems. When designing a pharmaceutical dermal or transdermal formulation, also referred to as a drug delivery vehicle, it is often necessary to add chemical penetration enhancers to improve the uptake. Today, the dominating means of investigating drug skin permeability and the effects thereon of different delivery vehicle excipients is by ex vivo/in vitro testing. There are, however, ethical problems involved in the study of percutaneous drug delivery using excised human or animal skin, or using animals in vivo (1,2). There are also issues of the translation of animal drug delivery data to humans (1, 2, 3). When using human skin, there are complications of inter- and intraindividual variation as well as of interlaboratory differences (1,4). Furthermore, ex vivo/in vitro testing yields no information about how compounds permeate skin.

In silico modeling is an interesting alternative to predict skin permeability and to gain insights into how compounds are absorbed in skin (5), but this requires both faithful models of the skin system and methods that can sample the process efficiently. A skin permeation prediction model relevant for human skin, encompassing the effects of penetration enhancers and other drug delivery vehicle excipients, could help improve transdermal formulation design.

**Figure 1:**
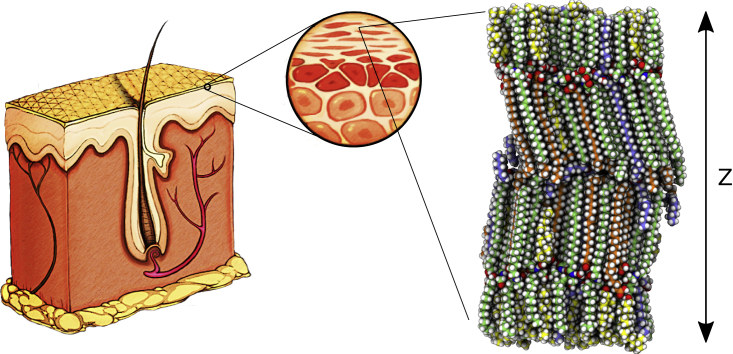
A schematic representation of the structure of epidermis and dermis. To the right is shown a snapshot of the atomistic model of the intercellular lipid structure of human stratum corneum that constitutes the skin’s main permeability barrier (6). The arrow indicates the main permeability direction, also referred to as the *Z* dimension. In the MD simulations the system is in practice repeated infinitely in the three dimensions. The carbon atoms are colored based on the molecule type, where ceramides are green, acyl ceramides (ceramide EOS) are light blue, free fatty acids are orange, and cholesterols are yellow. Hydrogen, nitrogen, and oxygen atoms are colored white, blue, and red, respectively. To see this figure in color, go online.

We have previously shown that an atomistic model of the skin’s barrier structure, i.e., the intercellular lipid matrix of the stratum corneum, validated against cryoelectron microscopy (cryo-EM) data from near-native skin (6) (Fig. 1), could be used in molecular dynamics (MD) simulations to predict the permeability of molecules through skin (7). These models made it possible to reproduce the effects of chemical penetration enhancers on the skin’s barrier structure at least qualitatively (7). This enabled a molecular-level understanding of their mechanisms of action, but the amount of sampling required and limited convergence made it difficult to accurately match experimental values. Improving the sampling efficiency for this type of complex system has led to a number of improvements of simulation protocols (8). We show better sampling of the free energy landscape and local diffusion coefficient of drugs passing through the skin’s barrier structure.

Here, we apply the accelerated weight histogram (AWH) method, using a two-dimensional (2D) reaction coordinate. This combines a spatial dimension, pulling the molecule across the barrier structure, with a second alchemical free energy dimension (8). It is expected that such sampling, allowing decoupling of the permeant, will be efficient in systems with long correlation times (9), such as the skin’s barrier structure. An improved agreement between calculated and measured permeability coefficients is presented. It is shown that, using the AWH method, ex vivo/in vitro measured skin permeability data can be accurately predicted, even though the in silico model selectively accounts for the skin’s barrier structure and not for the complete skin.

This study includes 20 chemical compounds with molar masses ranging from 18 to ∼300 g mol^−1^ and logP_o/w_ from −2.1 to 4.6. The distribution of these two molecular properties can be seen in Fig. S7. The experimentally measured permeability coefficients, log K_P_, of the molecules vary from 0.0 to −4.4 cm h^−1^. In addition to drug compounds, the studied permeants also encompass excipients, such as DMSO, ethanol, urea, and water. Octanol and decanol are not used as excipients, but were chosen to represent longer fatty alcohols. Those are commonly used as permeation enhancers, but do not have publicly available permeability coefficients. Benzene and toluene were included to verify that high-permeability coefficients can be correctly predicted. Transdermal, and topical, drugs have a wide spread of permeability coefficients, but their log K_P_ values are commonly found between −2.0 and −4.5 cm h^−1^. The permeability coefficient on its own cannot determine whether a drug is suitable for transdermal delivery or not. The required therapeutic concentration, solubility in the delivery vehicle as well as possible permeation enhancing techniques must also be taken into account.

The organization of the lipid barrier in human stratum corneum has received much attention for almost half a century. It has long been known that the lipids are organized as stacked layers (10,11). Important to highlight are models proposed by Swartzendruber et al. (12), Bouwstra et al. (13), Hill and Wertz (14), McIntosh (15), and Schröter et al. (16). In 2012, Iwai et al. (17) proposed a model based on CEMOVIS (cryoelectron microscopy of vitreous sections) images, of volar forearm and abdomen skin samples from five individuals, and simulation of EM images (18) from basic modeling of molecular arrangements. That structure proposal, as well as the experimental data and methods, served as the starting point for the model used herein (6), which in turn fits with a recent explanation of the skin barrier’s formation process based on cryo-EM (19). Another recently proposed model is the one by Mojumdar et al. (20), based on neutron diffraction studies of model lipid mixtures. Different experimental techniques, and sample treatment, have resulted in different observed periodicities of the lipid structure. Repeating units of primarily 4.5, 6.5 (or 4.5 + 6.5 nm), 11, and 13 nm have been reported in the studies mentioned above.

### MD skin permeation modeling

Quantitative structure-permeability relationship (QSPR) methods (21, 22, 23, 24, 25) are commonly used to predict skin permeability coefficients. QSPR models have proven very good at predicting permeability coefficients with reported mean square error as low as 0.48 cm^2^ h^−2^ (log units) and R^2^ of 0.7 (22,25). MD simulations constitute an alternative or complement to QSPR methods. MD simulations are significantly slower than QSPR methods, but not as limited to a specific applicability domain. Furthermore, incorporating the effect of formulation excipients on the permeability of active pharmaceutical ingredients (APIs) requires retraining of QSPR models, preferably for each combination of excipients. There have, nevertheless, been QSAR (quantitative structure-activity relationship) (26) studies involving chemical permeation enhancers, but information about these excipients’ mechanisms of action has been difficult to obtain. With MD simulations, formulation excipients can be incorporated in the skin’s barrier structure in their most probable concentrations, locations, and orientations, based on their local free energy potentials. This enables studying how they interact with the barrier and how they affect the permeability of one or more APIs. MD simulations cannot be expected to compete with QSPR models for pure permeability coefficient predictions, but they can provide important additional understanding. However, the first step must be to verify that the permeability coefficients calculated from MD simulations agree with experimental data.

One limitation of the MD simulation approach presented here is that only the structure of the main permeability barrier in skin (27,28), and not the complete skin, is studied in detail. However, it would be possible to complement the calculated permeability coefficients with mathematical models accounting for corneocyte permeability as well as for diffusion through the viable epidermis and dermis (29, 30, 31).

There are several groups that have studied permeation through skin using MD simulations prior to this. Das et al. (32) calculated the permeability coefficient of water, through one single CER:CHOL:FFA 2:2:1 hairpin bilayer in water at 300 K and reported log K_P_ −4.9 cm h^−1^. The permeability through a CER:CHOL:FFA 1:1:1 bilayer was only studied at 350 K and the log K_P_ was −3.5 cm h^−1^. Gupta et al. (33) calculated the permeability of 12 compounds through a fully hydrated CER:CHOL:FFA 1:1:1 hairpin bilayer. Ten of the compounds had experimental measurements. The molecular masses ranged from 18 to 106 g mol^−1^ and log P_o/w_ from −2.1 to 3.3. Their obtained mean square error was ∼13.8 cm^2^ h^−2^. The effect of lateral diffusion on water permeability through a model membrane was studied by Del Regno and Notman (34). They also used multiple levels of hydration in their work. Wang and Klauda (35) used a more complex structure, based on the work of Mojumdar et al. (20), involving two types of ceramides, in addition to ceramide EOS. They calculated the permeability coefficient of ethanol in good agreement with experimental results. MacDermaid et al. (36) employed another method of calculating the permeability coefficient, through a hairpin system including ceramide EOS. Their calculations involved fitting the diffusion pathway length constant using regression. Thereby they obtained a fit of the curve that was very good (log K_P_ mean square error ∼0.5 cm^2^ h^−2^) for nine molecules, with molecular masses ˂300 g mol^−1^ and log P_o/w_ ranging from −3.1 to 3.7.

In our previous studies we showed that it is possible to predict the permeability coefficient of different chemical compounds through an atomistic model of the skin’s barrier structure (6,7), illustrated in Fig. 1. Therein we used MD simulations to pull the permeants through the barrier system using a stiff spring and the forward-reverse (FR) method to calculate the permeability coefficients (37, 38, 39, 40). The lipid barrier model was developed by optimizing the structure based on the fit of simulated cryo-EM images, after MD equilibration, to CEMOVIS images. The model consists of fully splayed ceramides, free fatty acids, and cholesterol, as well as ceramide EOS and a low amount of water, located in the ceramide head group region (6).

Based on our previous proof-of-concept study (7) we have studied the effects of the pulling speed on the calculated permeability coefficients in more detail. In the supporting material (Fig. S1) we illustrate sampling problems using potentials of mean force (PMFs), i.e., the relative free energy difference across the system. In these examples we show testosterone—a permeant that we have experienced to need careful sampling to obtain accurate results. The general observation was that, with slower pulling speeds, the PMFs grew more detailed and the resulting free energy barriers became lower. We attribute this to the skin’s barrier lipid system being in a gel-like state and that the permeant needs to be pulled slowly to remain in a state near equilibrium, even if FR pulling is a nonequilibrium method (39). The experience that slower pulling speeds resulted in lower PMFs correlated in time with the publication by Wang and Klauda (35), in which they stated that 80 (approximately) stacked lipid bilayers of the skin’s barrier structure must be taken into account (29,35,41), since the calculated permeability coefficient is not actually an average speed through the system, even if its unit would indicate that. The effect is that the permeability coefficient should be divided by 80, or 1.9 be subtracted from its log value. When taking 80 stacked lipid bilayers layers into account for calculating the permeability coefficients using the data from our previous study (7) the log K_Pexp-calc_ mean square difference was approximately ∼11 cm^2^ h^−2^ (cf. ∼3 cm^2^ h^−2^ in the original work (7)). This led us to conclude that very slow pulling speeds might be required to obtain correct PMFs. This would, in turn, require very long simulation times for each pulling simulation (see Figs. S1 and S3)—potentially so long that entirely different sampling approaches are required to predict permeability with reasonable amounts of computing time. Since it is not the main topic of this paper we refer to the supporting material for more discussions about FR pulling and umbrella simulations.

A permeant’s skin permeability coefficient is mainly determined by the height of the peaks of its PMF across the skin’s barrier structure, assuming that the passage through the barrier structure is the rate limiting step for diffusion. On the other hand, the partitioning of a permeant from a formulation into the skin’s barrier structure primarily depends on the depth of the troughs of the permeant’s PMF. Therefore, it is important that the MD simulations can reproduce the whole PMF as accurately as possible, given the available computational resources. The calculated PMF of codeine (the *black curve* in the codeine subfigure in Fig. S5) serves as an example. The tight lipid packing in the ceramide fatty acid chain region (Z∼2.1 nm in the PMF) constitutes the main permeation barrier. Closer to the fatty acid tail ends (Z∼0.8 nm in the PMF) there is a combination of nonpolar interactions with the loosely packed lipid chains and hydrophilic interactions with the ceramide EOS ester moiety. These favorable interactions trap the permeant in that region. To improve the permeability of codeine we would propose to choose a permeation enhancer acting in the ceramide fatty acid chain region, rather than in the sphingoid chains. This information would not be accessible if the PMF sampling was poor (see the *red curve* for codeine in Fig. S5) or, presumably, if there were no ceramide EOS in the lipid barrier model structure.

### Skin permeability calculations with the AWH method

The AWH method (42,43) is an extended ensemble technique, in which an adaptive bias is used to flatten the free energy landscape. The applied bias enables sampling high free energy (low probability) configurations to the same extent as low free energy states. It is also possible to customize the target sample distribution to focus more on regions of the free energy landscape that are of higher interest.

**Figure 2:**
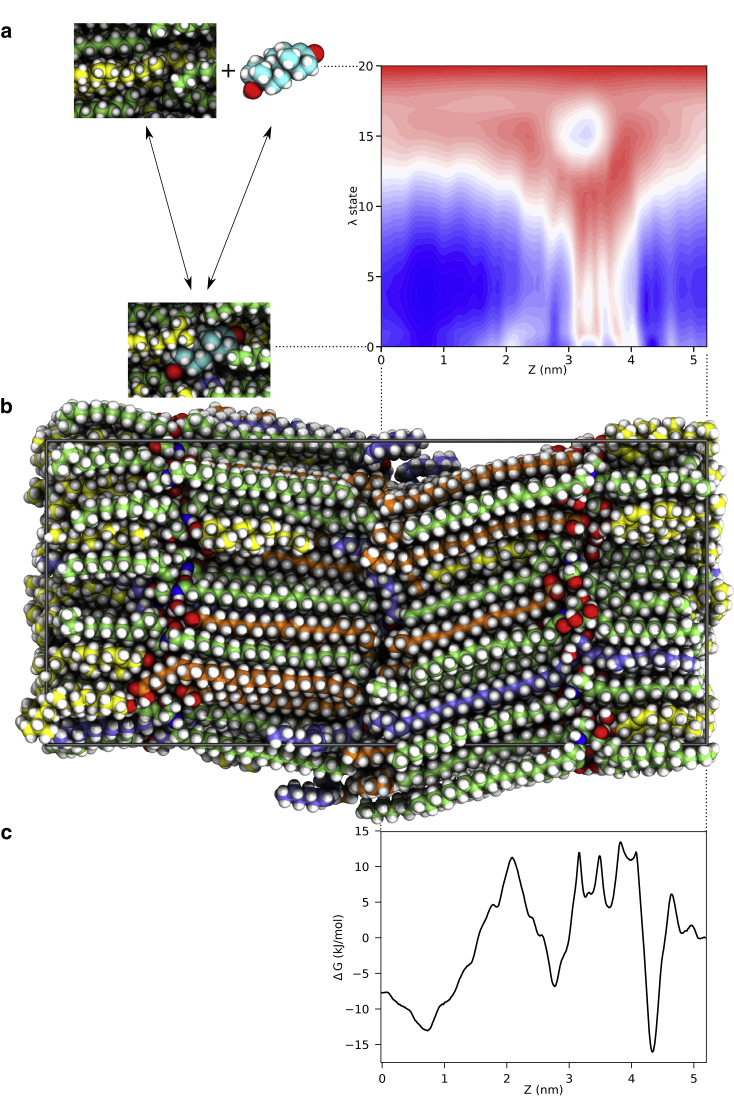
Calculating the PMF of testosterone through the skin’s barrier structure. (*a*) On the right, the 2D free energy landscape of testosterone where the alchemical free energy *λ* state is shown on the *y* axis—0 is fully interacting and 20 is fully decoupled (as illustrated by the miniatures to the left of the free energy landscape). On the *x* axis the *z* coordinate (in nm) through the simulated system is shown. (*b*) The skin’s barrier system, aligned to illustrate how the PMF is mirrored around 0 (the middle of the system). (*c*) The PMF of testosterone through the skin’s barrier structure of the fully interacting *λ* state (0 on the *y* axis in *a*). The PMF is from one set of simulations of 24 communicating AWH walkers, for a total of 11 *μ*s. AWH analyses do not give a reliable error estimate from one set of simulations, therefore there are no error bars presented in this plot. For error estimations based on multiple simulations see Fig. 3. To see this figure in color, go online.

In this work, we have used the AWH method to improve the sampling of the permeability of 20 permeants through the skin’s barrier system. Since GROMACS 2021 it is possible to use a 2D AWH sampling with an alchemical free energy dimension (8) combined with a spatial dimension, i.e., pulling the permeant across the skin’s barrier system. The alchemical free energy calculations imply that the interactions of the permeant with its surroundings can be gradually decoupled (switched on or off), thereby estimating the free energy of insertion. This means that there is no need for separate calibration of the PMFs in relation to the hydration free energy, or solvation free energy in the delivery vehicle of choice, as all points along the PMF will be calibrated compared with a vacuum state, in the same way as the solvation free energies are calculated. This also improves the sampling in regions with very slow diffusion (long correlation times), since turning off the interactions with the surroundings will let the permeant leave that region to sample other parts of the landscape, and later return in a different configuration (9). Fig. 2 shows a general overview of the method applied to calculating the PMF of testosterone through the skin’s barrier structure. With the AWH method it is also trivial to extend the simulations until they are sufficiently converged. This solves the primary sampling problem from previous studies, and it also enables us to calculate the local diffusion coefficient based on the AWH friction metric along the spatial dimension. The free energy of solvation in the solvent, or formulation, should still be calculated, which can be done in a separate 1D alchemical AWH simulation (8).

### Alchemical transformations in a gel-phase system

It is widely accepted that it is more efficient to perform electrostatic and Lennnard-Jones transformations (coupling/decoupling) in sequence to ensure that the Coulomb interactions are switched off before Lennard-Jones interactions are and vice versa (44). This avoids overlap of atoms when the attractive Coulomb forces are strong and the repulsive Lennard-Jones forces are turned off or are very weak. However, the skin’s barrier structure is in a gel-like state with very low mobility. We have found that the optimal orientation of a permeant molecule might be completely different in a state with its Lennard-Jones interactions turned on and Coulomb interactions turned off, compared with in its fully interacting state, and that the interconversion between the orientations can take very long when the Lennard-Jones interactions are turned on. For example, consider inserting (turning on interactions of) 1-decanol in the sphingoid chains of the skin’s barrier structure. If first turning on Lennard-Jones interactions, the probability of inserting the molecule with the hydroxyl group oriented away from the head group region, into the interface between the lipid chains, will be approximately the same as inserting it in its most favorable position with the hydroxyl group oriented toward the headgroup region. When turning on the Coulomb interactions it will be unfavourable if the molecule is oriented in the opposite direction and it will take very long for it to turn around. If turning on the Lennard-Jones and the Coulomb interactions simultaneously the electrostatic interactions will favor inserting the molecule correctly from the start.

In this project we decided to use the same, simultaneous interaction transformation, settings for both the 2D alchemical free energy/spatial AWH simulations as well as for the 1D AWH alchemical hydration free energy calculations, even if the efficiency might not be optimal for the latter. It should be noted that this problem is at least partially avoided if the alchemical free energy simulations are started from a fully interacting and equilibrated state or, as mentioned above, in a system with fast diffusion in all dimensions.

### Number of lipid bilayers to consider

Looking more closely at the published data it can be found that the assumed number of 80 bilayers (35,41) may need revision. Skin samples for skin permeability measurements are usually from the abdominal regions or the chest. Ya-Xian et al. (45) reported the number of cell layers in samples from the trunk, i.e., shoulder, chest, back, abdomen, and buttock, to be 13 on average, with a standard error of the mean (mean ± SE) of 0.4. This was the same source that was used to motivate choosing 15 layers, from a range of 6–20 (29,41). The intercellular lipids are reported to be located between the corneocytes, resulting in 12±0.4 intercellular regions of lipids, each consisting of a number of lipid bilayers. Since the structure of lipid layers above and below the bottom- and topmost corneocytes have not been clearly distinguished with CEMOVIS, these are not included in the number of lipid layers we take into account. These could possibly add to the total permeation resistance, but the difference would be small. Wang et al. (29) reported a range of three to nine bilayers between corneocytes, without a source, and choosing six bilayers as a reasonable average. Elias and Friend (11) have shown that there are commonly three to five continuous sheets between adjacent corneocytes, with an intercellular space ranging from 60 to 100 nm in neonatal mouse skin. From CEMOVIS studies of human skin a range of one to five bilayers between cells are commonly observed (17,46). After further discussion concerning observations from human skin we decided to settle upon an average of 2.5 intercellular bilayers with an approximate mean ± SE of 0.5.(47) This results in an average number of total bilayers of 30±6 (with the mean ± SE as uncertainty), based on 12±0.4×2.5±0.5. This revised number of layers has been used in our calculations.

## Methods

The atomistic model of the human skin’s barrier structure presented by Lundborg et al. (6), and originally called 33/33/33/75/5/0.3 (Relative composition in: molar % ceramides/molar % cholesterol/molar % free fatty acids/relative amount of cholesterol on ceramide sphingoid side/molar % acyl ceramide EOS (included in the relative ceramide concentration)/water molecules per lipid (not included in the molar % concentrations of the lipids)), was used as the starting structure for the simulations. The starting model had been equilibrated for approximately 270 ns, 250 ns of which were without restraints (6).

All MD simulations in this study (not including the data presented in Figs. S1 and S2) were performed using GROMACS 2021 and the second beta version of GROMACS 2022 (48, 49, 50), using the AWH method for alchemical free energy calculations (8,42,43). A source code modification enabled symmetrizing AWH sampling along a spatial (pulling) reaction coordinate dimension and also changed the number of AWH blocks for autocorrelation analysis from 64 to 128. These changes are available from the GROMACS gitlab repository (51).

van der Waals interactions had a cutoff of 1.2 nm with a smooth force-switch from 1.0 to 1.2 nm. The simulations were run without a dispersion correction for energy and pressure. Coulomb interactions were calculated using PME (52) with a radius of 1.2 nm. Bonds to hydrogen atoms were constrained using the P-LINCS algorithm (53,54). TIP3P (55) parameters were used for water molecules. For the lipid molecules, the CHARMM36 lipid force field (56,57) was used. Ceramide parameters were modified to more accurately reproduce the ceramide NP crystal structure (58), as described in (6). To allow a 3 fs integration time step, hydrogen atoms were made three times heavier by repartitioning the corresponding mass from their bound heavy atoms. Temperature was set to 305.15 K by using a stochastic dynamics integrator (also referred to as a velocity Langevin dynamics integrator) with a time step of 3 fs and with a time constant *τ* of 2 ps (corresponding to a friction constant of 0.5 ps^−1^). The pressure was set to 1 atm and controlled using a stochastic cell rescaling barostat (59) with a time constant of 1.0 ps and a compressibility of $4.5\times 10^{-5}$ bar^−1^. In the skin’s barrier system the pressure coupling was semi-isotropic with no compressibility in the *Z* dimension. During the development of the calculation methods, for all permeants, at least one simulation was performed with unmodified hydrogen masses and a 2 fs time step to verify that the longer (3 fs) time step did not affect the results. However, most of those 2 fs time step simulations were run with other parameters. To verify that there were no significant differences, with the same simulation settings as in the simulations used for producing the permeation results, four permeants were re-run with standard hydrogen masses and 2 fs MD simulation time step. As can be seen in Figs. S10 and S11 there were no large differences in the PMFs and diffusion coefficients.

Along the alchemical free energy dimension, 21 equidistantly distributed *λ* states were used for decoupling both van der Waals and Coulomb interactions simultaneously. There were 10 or 100 steps between each Monte Carlo coordinate sampling, along the alchemical free energy *λ*-value and spatial position across the lipid structure. There were 10 samples per update of the $f_{\lambda }$ bias. There was no observable difference between sampling every 10 or 100 steps, but the simulations were faster when sampling less frequently. Soft-core transformations (60) with α=0.5 and σ=0.3 nm were applied to both the van der Waals and Coulomb interactions of the solute.

Topologies, i.e., inter- and intramolecular interaction parameters, for all permeants and formulation components except water, were generated using STaGE (61), which in turn uses Open Babel (62) and MATCH (63) to generate GROMACS topologies compatible with the CGenFF (64) CHARMM force field.

AWH simulations can be performed using multiple walkers. This means that the simulations are run in parallel and that the sampling is communicated between them when the bias and PMF are updated. That means that they share the same bias and effectively share the sampling of the free energy landscape. Since the bias and PMF are shared between the walkers multiple independent sets of simulations need to be performed to estimate the uncertainty. However, currently the AWH friction metric is not communicated. This means that the diffusion coefficient is calculated separately for each AWH walker and that it is possible to estimate an uncertainty of the diffusion coefficient from one set of AWH simulations with multiple walkers. Each AWH walker samples both reaction coordinate dimensions, but it is not necessary that each individual walker samples all points in the 2D free energy landscape.

All images representing molecules were prepared using Tachyon (65) in VMD (66).

### Hydration free energy calculations

The hydration free energy was calculated starting with a water box large enough to solvate the molecule. The molecule was inserted into the system at a random position with its interactions to the surroundings turned off, as if in vacuum. AWH was used to sample the alchemical free energy *λ* states (8) to calculate the solvation free energy. The initial AWH histogram size, which determines the initial update size of the free energy, was set indirectly by specifying an estimate of the diffusion constant along the alchemical free energy *λ*-axis in combination with a rough estimate of the initial error. An input diffusion constant of $1\times 10^{-3}$ ps^−1^ was used for the hydration free energy calculations, which means that it is estimated to take approximately 1 ns to cross the alchemical dimension for one AWH walker. The initial error was set to 10 kJ mol^−1^. Sixteen communicating AWH walkers were run in parallel, with the requirement that only simulations that covered the whole alchemical reaction coordinate counted toward the covering check in the initial AWH stage. The simulations were 60 ns long per walker for a total simulation time of 960 ns per solute. For a more detailed description of alchemical hydration free energy calculations using AWH see Lundborg et al. (8).

### Permeability coefficient calculations

The permeability coefficient, $K_{P}$, and the permeation resistance, *R*, were calculated as follows (67):

(1) $$\frac{1}{K_{P}}=R=\int_{Z_{1}}^{Z_{2}}\frac{e^{\beta \text{Δ}G\left(z\right)}}{D\left(z\right)}dz,$$

where ΔG is the difference in free energy compared with the reference state, and *D* is the local diffusion coefficient across the skin’s barrier structure (dz).

As mentioned above, we calculated the permeability through 30±6 layers of the lipid barrier. The resulting permeability coefficient calculation was:

(2) $$\frac{1}{K_{P}}=R=30\pm 6\times \int_{Z_{1}}^{Z_{2}}\frac{e^{\beta \text{Δ}G_{rel.water}\left(z\right)}}{D\left(z\right)}dz,$$

where $\text{Δ}G_{rel.water}$ is the free energy relative to the hydration free energy. When calculating the permeability coefficients, an additive constant of each PMF was chosen so that the PMF was never below 0, similar to what Wang and Klauda (35), as well as Venable et al. (68), have done before.

The local diffusion coefficient D(z) across the barrier is estimated from the friction metric g(z) calculated in the AWH simulation (69), via an Einstein relation $D\left(z\right)=g^{-1}\left(z\right)$. This rests on the assumption that the motion of the permeant may be approximated as a slow Markovian process, a condition that appears to be well satisfied in the present application. The AWH friction metric is calculated using the autocorrelation of the bias force from the whole simulation, including the AWH initial stage (69). We have verified that effects on the friction metric, from the larger bias fluctuations in the initial stage, are small. The diffusion coefficient from a shorter testosterone simulation with a constant bias, using the bias from a 900 ns simulation as input, was evaluated. It was similar to output at corresponding times from the simulations with adaptive bias in the regions sufficiently sampled. The diffusion coefficient converged toward the results from the longer simulation. To reduce the noise of the local diffusion coefficient curves, an 0.2 nm wide rolling median filter was applied. When symmetrizing the sampling, along the spatial dimension, by using the absolute coordinate values and accounting for the AWH bias across the sampling boundaries there are usually artificial spikes at the edges of the PMFs. These were removed by setting the two lowest (0.005 and 0.015 nm) and highest (5.200 and 5.210 nm) points in the PMF to the value of their neighbors (0.025 and 5.19 nm, respectively). These minor adjustments had no effects on the calculated permeability coefficients.

At the start of each AWH walker simulation the permeating molecule was inserted in the lipid barrier structure at a random position with all interactions to its environment turned off. The free energy profile through the skin’s barrier structure was calculated using a 2D AWH setup, using a harmonic potential to steer the permeant across the system, also referred to as the *Z* dimension, and an alchemical free energy reaction coordinate (8). This allows sampling the free energy along the permeation direction and also the relative insertion free energy of the permeant from vacuum. In turn, this enables a direct calibration to the hydration free energy, since the vacuum state is the same in both cases. As when calculating the hydration free energy, the estimated AWH initial error was set to 10 kJ mol^−1^. The AWH input diffusion constant was set to $3\times 10^{-5}$ nm^2^ ps^−1^ for the spatial pulling dimension and $5\times 10^{-5}$ ps^−1^ along the alchemical free energy dimension. The input diffusion constant affects only the AWH histogram size, it does not affect the computed diffusion coefficient, which is obtained from the AWH friction metric during data analysis. The AWH force constant along the spatial *Z* dimension (normal to the lamellar stack), steering the permeant relative to the ceramide fatty acid chains using a harmonic pull potential, was set to 25,000 kJ mol^−1^ nm^−2^. The force constant also determines the resolution along the reaction coordinate dimension. For each permeant, five sets of simulations were run with heavy hydrogen atoms (see above) and a 3 fs integration time step. These were run using 24 communicating walkers, each running for 450 ns. The covering check in the initial AWH stage only took into account simulations that covered the whole alchemical dimension and at least a diameter of 0.8 nm along the spatial dimension. From these simulations a combined diffusion coefficient was calculated using the AWH friction metric from all contributing walkers. The combined PMF was derived from the average of the independent PMFs from the five sets of simulations. The combined outputs were used to calculate the permeability coefficients presented in Table S1.

Along the alchemical free energy dimension it is the end states, i.e., the fully interacting and fully decoupled states, that are of highest interest, as the difference in free energy between them corresponds to the probability of transferring the permeant from vacuum into the skin’s barrier structure. Therefore, the target distribution used in these simulations put more weight on the end states, especially the state with interactions fully turned on. The target distribution along the alchemical free energy dimension that was used is shown in Fig. S4. Along the spatial pulling dimension the target distribution was uniform.

One-dimensional AWH simulations of testosterone across the barrier structure were also performed for evaluation and comparison with the 2D AWH results. They were run using 4 independent sets of simulations, each using 24 communicating AWH walkers running for 600 ns. The simulation settings were the same as specified above, except that there was no alchemical free energy dimension.

## Results

### Permeability coefficient calculations

**Figure 3:**
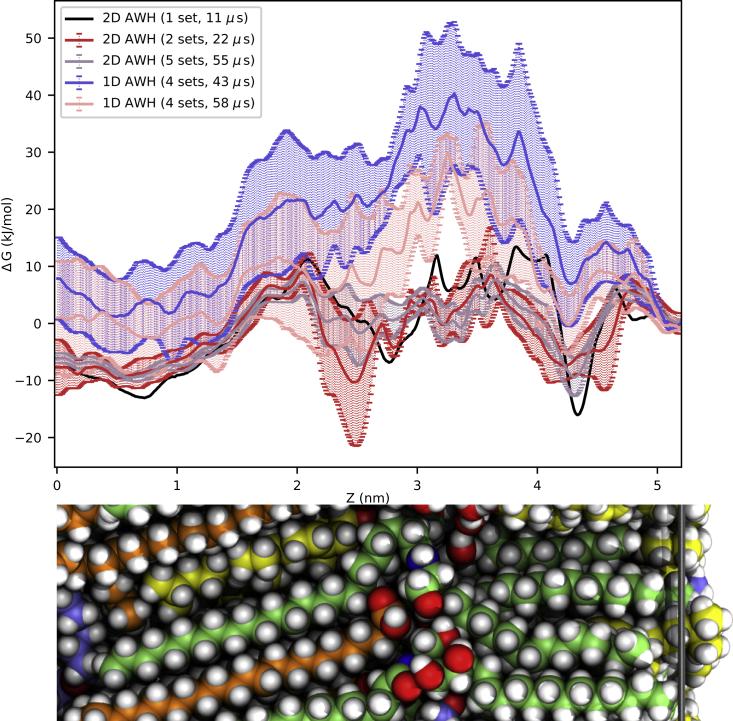
Faster convergence with 2D AWH. The PMFs of testosterone are from 2D spatial/alchemical AWH (in the fully interacting alchemical *λ* state) and 1D AWH with only a spatial reaction coordinate dimension. In both cases the PMFs are symmetrized and only half the PMFs are presented, and the PMFs are calibrated to 0 at the ceramide sphingoid chain interface (5.2 nm) to make comparisons easier. The uncertainties represent one standard error of the mean between the independent sets of simulations. The 11 *μ*s 2D AWH results are from one set of 24 communicating walkers. AWH analyses do not give a reliable error estimate from one set of simulations, therefore there is no error presented for the 11 *μ*s AWH plot (in black). The 1D AWH simulations of 58 *μ*s required approximately the same computation time as 22 *μ*s of 2D AWH. A snapshot of the molecular system is shown below the plot to indicate where the head groups are located (at ≈ 3.1–3.3 nm). To see this figure in color, go online.

Running 1D AWH simulations, with only a spatial reaction coordinate dimension across the barrier structure, did not sample the free energy profile of testosterone efficiently. None of four sets of simulations, each consisting of 24 communicating walkers sharing the same AWH bias and running for 600 ns for a total simulation time of 14.4 *μ*s in each of the four independent simulation sets, sampled enough to cover the reaction coordinate with the condition that a single walker must cover a diameter of 0.8 nm to be taken into account. It is possible that this requirement was too strict for a system with inherently slow diffusion. This meant that the 1D AWH simulations did not leave the AWH histogram equilibration stage as part of the AWH initial stage. Increasing the AWH input diffusion constant would have increased the bias more quickly, meaning that covering of the reaction coordinate would have been quicker. However, that might also have resulted in too high free energy barriers in the PMF. The average PMF from the four sets of 1D AWH simulations is compared with the results from the 2D AWH simulations, including an alchemical dimension, in Fig. 3. The results from four sets, of totally 58 *μ*s, of 1D AWH were worse than after 11 *μ*s of 2D AWH with an alchemical reaction coordinate; i.e., assuming that the five sets (55 *μ*s) of 2D AWH simulations are closest to convergence. The PMF output from 1D AWH were fairly similar to the FR pulling simulations shown in Fig. S3, but with higher uncertainty, i.e., a larger difference between the four sets of simulations.

**Figure 4:**
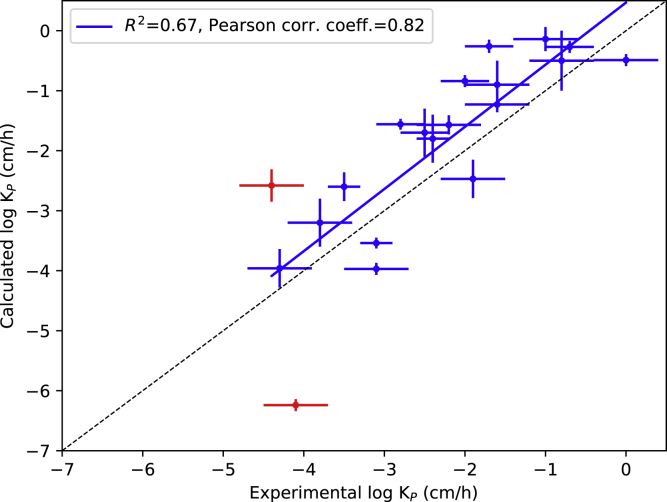
The correlation between experimental log K_P_ and those calculated from MD simulations. The blue line shows the linear regression, whereas the dashed black line is the identity line. The two largest outliers, urea and hydromorphone, are marked in red (still included in the linear regression). The error bars represent 1 mean ± SE (standard error of the mean), which is approximated for experimental values. Details about the approximation is presented in the header of Table S1. To see this figure in color, go online.

The results from the 2D AWH simulations agreed well with the experimental data, thanks to the improved sampling of the free energy landscape. The relationship between calculated and experimental permeability coefficients are presented in Fig. 4. The numerical results are presented in Table S1. The largest outlier was urea, followed by hydromorphone with log K_P_ deviations of −2.1 and 1.8 cm h^−1^, respectively. The calculated permeability coefficients tended to be overestimated, on average by 0.38 log units. The average absolute log K_P_ deviation was 0.84 cm h^−1^ (see Table S1). If excluding urea and hydromorphone it would be reduced to 0.71 cm h^−1^.

In the supporting material (Figs. S5 and S6), the PMFs and local diffusion coefficients across the skin’s barrier structure are shown. There are also comparisons to our previously published results (7) using FR pulling. Fig. S7 shows the molecular mass and log P_o/w_ properties of the permeants as well as the differences between calculated and experimental log K_P_. No obvious clusters with high errors can be observed.

### Convergence of diffusion coefficients and PMFs

We have observed that the calculated diffusion coefficients depend on the simulation time, with lower diffusion coefficients the longer the simulation time. In general, the same trend is also observed regarding the PMF, i.e., lower free energy barriers with more sampling. We have seen this using all MD simulation sampling methods mentioned herein: AWH (1D with a spatial dimension and 2D with an added alchemical free energy perturbation dimension), umbrella sampling, and FR pulling. Examples of the effect on the calculated water diffusion coefficient from different methods and simulation settings are shown in Fig. S13. For permeability coefficient calculations, all simulations in this study were run in 5 independent sets of 24 communicating AWH walkers, each running for 450 ns. Our observations are that the calculated permeability coefficients are largely unaffected when increasing the simulation time by a factor two, from 450 to 900 ns per AWH walker (see Table S2 and Figs S12 and S13).

In general, the resulting PMFs from 2D AWH are more stable, when extending the total simulation time, than those from 1D AWH and FR pulling. When simulating for longer periods, the calculated PMFs from 1D AWH and FR pulling are observed to approach those from 2D AWH.

## Discussion

When comparing PMFs of testosterone from the 2D AWH simulations performed in this study to previously performed (unpublished) FR pulling simulations (Fig. S3), it is clear that the PMFs from the 30 *μ*s pulling simulations are closer to the PMFs from AWH. The calculated log K_P_ for testosterone from the FR pulling simulations (taking the 30 stacked lipid bilayers into account) was −3.8 cm h^−1^, compared with −1.7 cm h^−1^ from the AWH simulations and −2.5 cm h^−1^ for the ex vivo/in vitro measurements (with reports ranging from −1.9 to −3.2 cm h^−1^ (70, 71, 72, 73), corrected to a temperature of 305 K).

The 2D AWH sampling, with one alchemical dimension, was also more efficient than using a 1D approach with only a spatial reaction coordinate, as shown in Fig. 3. The main drawback with an alchemical free energy reaction coordinate dimension is that the simulation throughput is lower compared with FR pulling simulations as well as 1D AWH simulations using only a spatial coordinate. However, the possibility to more accurately sample the free energy landscape makes it attractive for problems that are difficult to address with conventional methods, such as permeability coefficient predictions in gel-phase systems, like the skin’s barrier structure.

Another advantage is that it is easier to prepare permeability calculation simulations using an alchemical dimension than with conventional procedures, as the permeant molecule can be inserted anywhere in the system with all interactions turned off. The AWH simulations will then turn on the interactions where favorable. On the contrary, with standard FR pulling, as well as with 1D AWH pulling or umbrella simulations, the molecule must be grown into the system and/or pulled to suitable starting positions, followed by equilibration of the simulation system, before the production simulations are started. To determine the free energy landscape without an alchemical dimension in the calculation, it would also be necessary to know the free energy of inserting the molecular for at least one position along the barrier structure; otherwise the PMF cannot be calibrated compared with a solution or formulation.

The permeability coefficient calculations presented herein only account for the passage of a chemical compound across the skin’s barrier structure. Lateral diffusion within the skin’s barrier structure is not calculated, and permeation through the stratum corneum cell interiors, as well as through viable cell layers of epidermis, are ignored. Likewise, potential contributions from the corneocyte cell lipid envelopes are not included. While these are admittedly approximations, the contributions from these factors are expected to be negligible in comparison with the permeation resistance across the skin’s barrier structure for all but very lipophilic compounds (74). It is primarily the permeability across the skin’s barrier structure that can be tuned using chemical permeation enhancers, and thereby exploited in transdermal drug delivery design.

As can be inferred from Wang and Klauda (35) and from Scheuplein and Blank (74), as well as from Eq. 20 in Marrink and Berendsen (67), and discussed in the introduction, the permeability coefficient does not correspond to an average permeation speed through the skin, but will become lower the thicker the skin sample. Therefore, the permeability coefficient through the skin’s barrier structure alone would be higher than through full-thickness skin samples used in ex vivo/in vitro permeability measurements. We would therefore expect calculated permeability coefficients, referring exclusively to the skin’s barrier structure, to be slightly higher than those measured ex vivo/in vitro through full-thickness skin, assuming that the skin samples are intact. Our calculated log K_P_ is 0.38 cm h^−1^ higher than experimental results on average, and 0.44 cm h^−1^ higher if excluding urea and hydromorphone—the two largest outliers.

Measuring skin permeability using diffusion cells, such as Franz cells, requires long experiment times: how long depends on the permeant’s lag time (75). It is common to run experiments for 24–72 h (75). In extreme cases, with low permeability and long lag times, this might even be insufficient. However, long-time exposure to water leads to skin degradation and increased skin permeability (75,76). These factors can make it difficult to compare theoretical results with those obtained ex vivo/in vitro, since the ex vivo/in vitro measurements may sometimes be too short for the permeation lag time and too long for keeping the skin sample intact.

On the other hand, the main disadvantage with MD simulations is that they take a lot longer to run compared with in vitro/ex vivo experiments. This is even more striking when it comes to studying the gel-like lipid barrier structure in stratum corneum, which is the problem we have mitigated using more efficient sampling. Even if MD simulations do not need any training, in contrast to QSAR models, they still depend on the quality of the force field that is used. Given the resource requirements, MD simulations should be considered a complement to laboratory diffusion cell experiments, as well as QSPR calculations or other mathematical models. There is no reason to exclusively depend on one method.

The largest outliers in this study (see Table S1) were urea (log K_P_ diff. −2.1 cm h^−1^) and hydromorphone (log K_P_ diff. = 1.8 cm h^−1^), followed by salicylic acid and ethanol. The correlation plot of the results, excluding urea and hydromorphone, is shown in Fig. S8. The permeation enhancing effects of urea (77) were not taken into account in these simulations, which could be a reason for the discrepancy. QSPR simulations also underestimate the permeability coefficient of urea, but only by 0.6 log units on average (21,22,25,78), so another explanation is needed. It has been proposed that small hydrophilic permeants pass through “polar pathways,” shunts, or through lipid defects (79). Lateral diffusion in the lipid barrier structure may be important to find these more permeable regions (34), even if their exact nature is not yet fully explained. For hydromorphone, there is a remarkable similarity between the average of three different predicted log K_P_ values obtained using mathematical QSPR models of −3.2 cm h^−1^ (21,22,25,78) and our predicted log K_P_ at 32°C of −2.9 cm h^−1^, contrasting with the experimentally obtained value of −4.8 cm h^−1^ at 37°C (80), with no corrections for pH/pK_a_. This discrepancy between predicted and experimental data may call for re-investigation of the experimentally measured permeability coefficient of hydromorphone, preferably from multiple laboratories, although it could of course also indicate permeation properties not picked up by QSPR models either. Looking at Fig. S7, the calculated and experimental log K_P_ square difference does not seem to correlate specifically with molecular mass or log P_o/w_.

## Conclusion

We have shown that the model of the skin’s barrier structure that was proposed a few years ago (6) can be used to accurately predict permeability coefficients of a large spectrum of molecules, with clearly better computational efficiency than previous methods.

To our knowledge, this is the first report describing the use of a 2D reaction coordinate with one spatial dimension and one alchemical free energy dimension. It is also the first study to employ the AWH method to sample such a free energy landscape. The obtained PMFs are detailed and some of their features only hinted at when using FR pulling simulations with very slow pulling speeds. When taking the multiple bilayers of the skin’s barrier structure into account, our previously published calculations underestimated the skin permeability coefficients (7), largely explained by the inability of the FR pulling method to properly sample the free energy landscape.

Experimental permeability coefficient measurements are associated with variations between laboratories as well as with inter- and intraindividual differences. This makes it treacherous to strictly compare calculated permeability coefficients with experimental data. This needs, particularly, to be taken into account when creating QSPR models (4). A suggested approach for building a reliable QSPR model would therefore be that either all training data should come from one laboratory using the same experimental setup for all compounds or, preferably, there should be measurements from several laboratories, using the same methods for each compound. When predicting permeability coefficients using MD simulation there is no need for training the method with experimental data, but reliable experimental measurements are required for verifying the techniques and simulation results.

The correlation between calculated and experimental permeability coefficients presented here proves the usefulness of MD simulation for transdermal drug delivery design, although the method’s precision may presently not always allow for differentiation of very similar molecules.

We do not argue that MD simulations should replace ex vivo/in vitro measurements, but the two methods can complement each other. Perhaps most important for transdermal drug delivery design is that MD simulation can explain experimental ex vivo/in vitro data by predicting where in the skin’s barrier structure the main permeation barriers are located for each specific permeant. This knowledge, along with the PMFs (in effect partition profiles) of chemical permeation enhancers, can help selecting suitable drug delivery formulation excipient combinations tailored for each specific API.

## Author contributions

M.L., L.N., C.W., and E.L. designed the research. M.L., J.L., B.H., and C.W. developed the simulation protocols. M.L. carried out all simulations and analyzed the data with assistance from C.W. All authors wrote the article, with M.L. being the primary author.

## Data Availability

The input data and parameters are available for download from https://doi.org/10.5281/zenodo.5883410.

## Supporting material

Document S1. Figures S1–S13, Tables S1, and S2

Document S2. Article plus supporting material
