# Supplementary material for: Skin permeability prediction with MD simulation sampling spatial and alchemical reaction coordinates
Source: Biophys J. 2022 Sep 13;121(20):3837–49. doi: 10.1016/j.bpj.2022.09.009 (PMC9674988; doi:10.1016/j.bpj.2022.09.009)
Supplement: Document S1. Figures S1–S13, Tables S1, and S2 [file mmc1.pdf]

Biophysical Journal, Volume *121*

Supplemental information

Skin permeability prediction with MD simulation sampling spatial and alchemical reaction coordinates

Magnus Lundborg, Christian Wennberg, Jack Lidmar, Berk Hess, Erik Lindahl, and Lars Norlén

Supplement

Supplementary information for: Skin Permeability Prediction with MD Simulation Sampling Spatial and Alchemical Reaction Coordinates

Magnus Lundborg[1,*], Christian Wennberg[1], Jack Lidmar[2], Berk Hess[3], Erik Lindahl[4,5], and Lars Norlén[6,7,*]

[1]ERCO Pharma AB, Science for Life Laboratory, Solna, Sweden
[2]Department of Physics, KTH Royal Institute of Technology, Stockholm, Sweden
[3]Department of Applied Physics, Science for Life Laboratory, KTH Royal Institute of Technology, Solna, Sweden
[4]Department of Biophysics and Biochemistry, Science for Life Laboratory, Stockholm University, Solna, Sweden
[5]Department of Physics, Swedish e-Science Research Center, KTH Royal Institute of Technology, Stockholm, Sweden
[6]Department of Cell and Molecular Biology (CMB), Karolinska Institutet, Stockholm, Sweden
[7]Dermatology Clinic, Karolinska University Hospital, Stockholm, Sweden
[*]Correspondence: magnus.lundborg@ercopharma.com and lars.norlen@ki.se

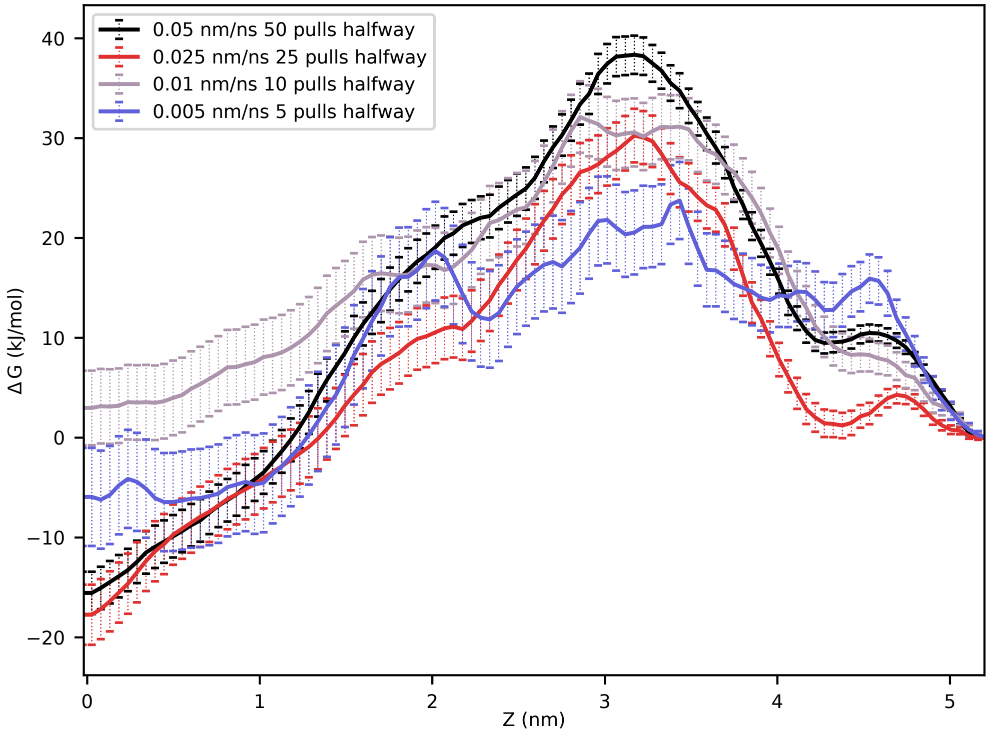

Figure S1: PMFs of testosterone, from forward-reverse pulling simulations, with different pulling speeds. The results are from 10 µs of sampling and the PMFs are calibrated to 0 at the ceramide sphingoid chain interface (5.2 nm), to make comparisons easier. The general trend is that slower pulling speeds give lower free energy barriers and more detailed PMFs. However, fewer pulls also means a larger variability. Two permeants were pulled halfway through the system in order to shorten the simulations.

S1 METHODS

When running AWH simulations it is possible to run multiple simulations in parallel, referred to as AWH walkers. The simulations can share their sampling, and thereby use the same bias, by communicating at regular intervals. This can improve the total simulation throughput compared to running a single simulation for a longer time. For the simulations of the lipid barrier structure 24 MD simulations with communicating AWH walkers were run, whereas 16 communicating AWH walkers were used for the hydration free energy calculations. The AWH histograms were equilibrated as part of the initial stage, using the criterion that at least 80 % of the target region was sampled within 20 % of the target distribution.

During the course of this project it was discovered that GROMACS 2021 running AWH with an alchemical free energy dimension did not properly take PME contributions into account along the alchemical dimension when calculating PME on a GPU accelerator. After this bug was fixed all hydration free energy calculations were rerun as well as at least two independent sets, with 24 communicating AWH walkers, each running 450 ns, calculating the permeability through the barrier. It was found that a systematic shift in the PMF, relative to the vacuum state, in the range of 0 kJ mol^{-1} to 13 kJ mol^{-1} depending on the permeant, was the only significant difference. The PMF results from the GROMACS version with the bug were shifted to align with the new correct output.

S2 RESULTS

S2.1 Comparisons to other MD simulation methods

In the data presented in this section we pulled testosterone using a constraint, i.e., an infinitely stiff spring, to avoid having to select a suitable spring constant for each permeant (1). Compared to the 2018 study we also equilibrated the system longer after inserting the two copies of the permeant into the system, (20 ns instead of 1 ns). As can be seen in Fig. S1 the results, for testosterone, were very dependent on the pulling speeds. As these results are not the focus of this study — they are just included for comparison — we will refer to the original paper(2) for a more thorough description of the methods.

We also evaluated umbrella sampling simulations for calculating the PMFs. We inserted four copies of testosterone, evenly

distributed along the reaction coordinate, and grew them in, by turning on their interactions with their environment, over a period of 1 ns. This was repeated in 80 steps along the reaction coordinate, giving 320 umbrella sampling positions at random lateral positions. During the umbrella simulations the permeants were held in place using a spring constant of 5000 kJ mol^{-1} nm^{-2}. The 80 simulations were run for 150 ns (for a total of 12 000 ns). To estimate the equilibration times (how much to discard in the beginning of the simulations) a time window of 100 ns, with a shifting starting time, was analyzed. As can be seen in Fig. S2, even if the difference between the shortest and longest equilibration times was not statistically significant, there was an obvious systematic drift in the PMF with increasing equilibration times, most clearly seen in Fig. S2b. This indicated that discarding 50 ns (out of 150 ns) might not be enough. While, in the case shown here, the effect is not very large for the symmetrized PMF (Fig. S2a) the long times to reach convergence was not very attractive. Comparing Fig. S2a) to the previously published results from FR pulling simulations(2), shown by the red curve in the Testosterone sub figure in Fig. S5, indicates that the height of the free energy profile from these umbrella simulations roughly matched the ones from FR pulling at a pulling speed of 0.2 nm ns^{-1}. However, they required approximately the same total simulation time as the pulling simulations at 0.005 nm ns^{-1}. We expect that the umbrella sampling simulations can be further optimized, and do not claim that umbrella sampling is better or worse than FR pulling. Both methods face similar challenges with the long correlation times of the skin's barrier system existing in a gel-like state.

S2.2 Comparison of testosterone PMFs from 2D AWH to FR Pulling

Using the slowest pulling speed shown in Fig. S1 we compared the data from FR pulling simulations at 10 and 30 µs to the two-dimensional AWH setup, that was used for this project, with 1, 3 and 5 independent sets of simulations (11 , 32 and 55 µs). The results are presented in Fig. S3 and show that the differences were small between the AWH simulations with 3 and 5 sets and that the results from 30 µs FR pulling were further away from the final AWH results than the results after 1 set (11 µs) of AWH simulations. It can also be noted that the results from FR pulling simulations seem to approach those from AWH, but since the output from 30 µs of FR pulling is still far from the AWH results it can be concluded that many more FR pulling simulations, in order to achieve a longer total simulation time, would be needed to come close to the AWH results. Looking also at the testosterone diffusion coefficient profile in Fig. S6 it can be concluded that the largest differences between FR pulling and AWH are observed in the region with the slowest diffusion, i.e., Z ~2.5 nm to ~4.5 nm.

S2.3 Permeability Coefficient Calculations

The permeability coefficients were calculated using Eq. 1 (in the main paper) using the PMF and diffusion coefficient profiles presented in Figs. S5 and S6. The results are presented in Table S1 and in Fig. 4 (in the main paper).

In this study we have been focusing on the permeability coefficients of neutral (non-ionized) forms of the permeants. There were eight molecules that are ionizable under the *ex vivo/in vitro* conditions: codeine, diclofenac, hydromorphone, ibuprofen, lidocaine, naproxen, nicotine and salicylic acid. For most of them there are published permeability coefficients available, whereas for codeine and hydromorphone the corrections by Abraham and Martins (3) were used. In order to examine whether the neutral forms of ionizable permeants were in agreement with experimental results we divided the molecules into two groups, separating those that are not ionizable under the *ex vivo/in vitro* conditions from those that are ionizable. The correlation plots from these analyses are presented in Fig. S9 and show that there is no clear difference between the two groups, but the permeability coefficients of the set of non-ionizable compounds are better predicted by MD simulations.

We also studied whether there was any clear correlation between permeant molecular mass or log $P_{o/w}$ and large differences between experimental and calculated permeability coefficients. The results are presented in Fig. S7.

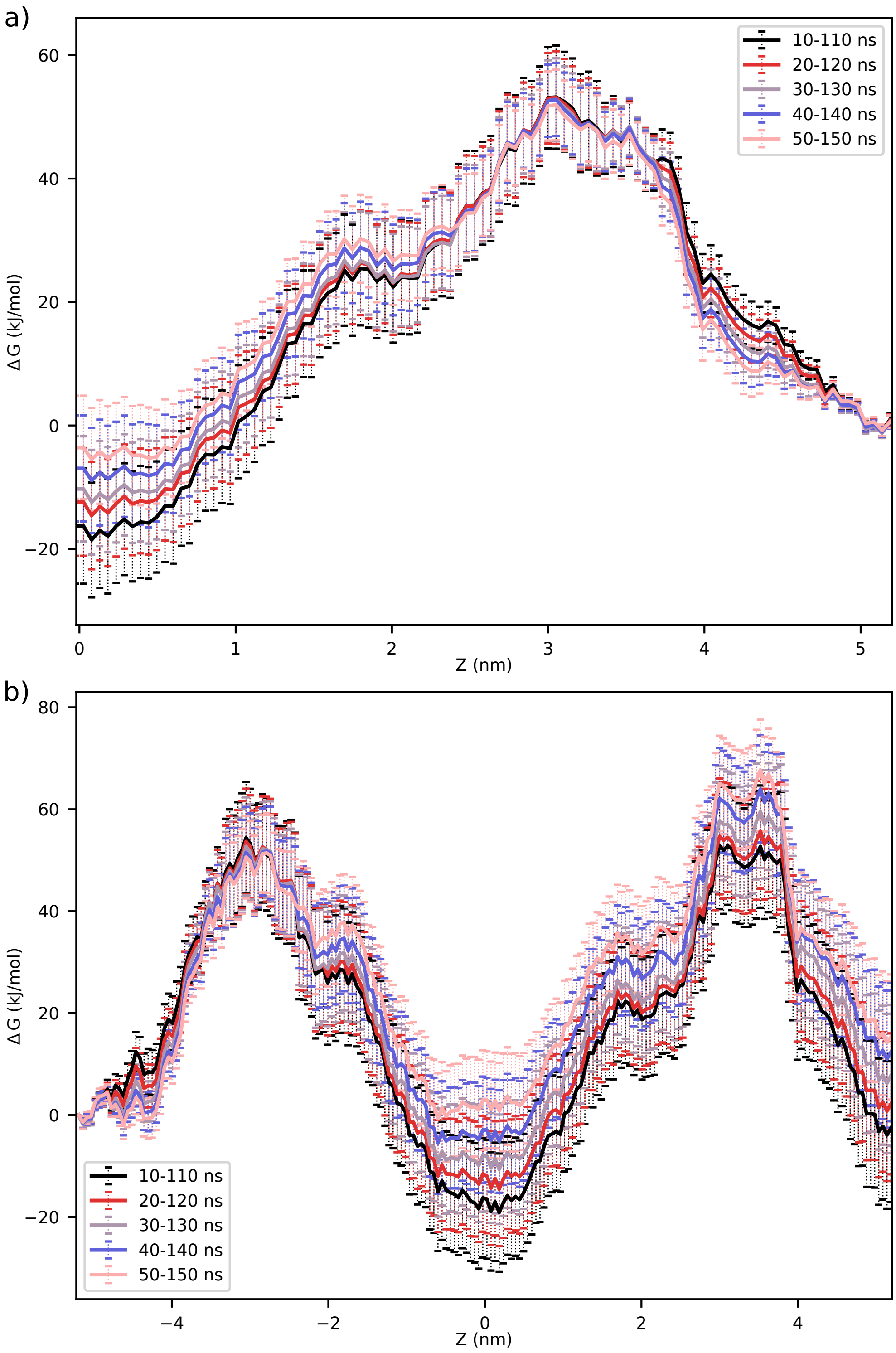

Figure S2: PMFs of testosterone from umbrella sampling simulations with increasing equilibration time. In a) the data is enforced to be periodic, with connected end points, and symmetrized. The PMFs are calibrated to 0 at the ceramide sphingoid chain interface (5.2 nm), to make comparisons easier. In b) the data is not symmetrized without connected end points (the PMF is set to 0 only at −5.2 nm).

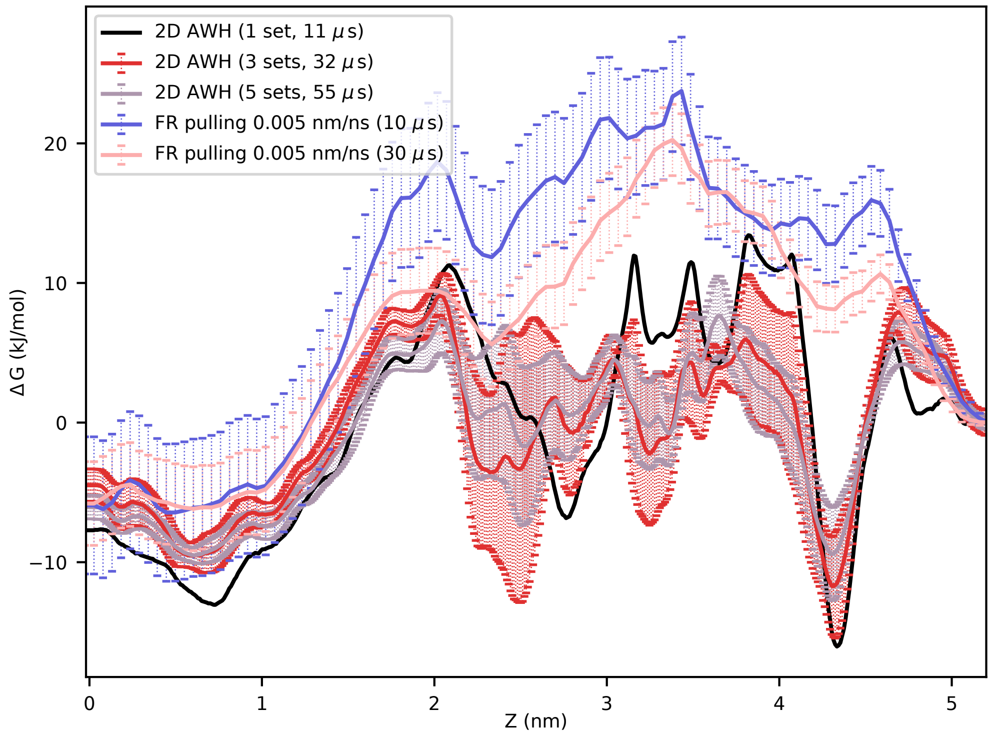

Figure S3: PMFs of testosterone comparing the 2D spatial/alchemical AWH (in the fully interacting alchemical λ state) results to slow forward-reverse pulling experiments. In both cases the PMFs are symmetrized and only half the PMFs are presented and the PMFs are calibrated to 0 at the ceramide sphingoid chain interface (5.2 nm), to make comparisons easier. AWH analyses do not give a reliable error estimate from one set of simulations, therefore there is no error presented for the 1 set, 11 µs AWH plot (in black). In the pulling experiments two permeants were pulled halfways through the system in order to shorten the simulations. FR pulling simulations of 30 µs required approximately the same computation time as 11 µs of 2D AWH.

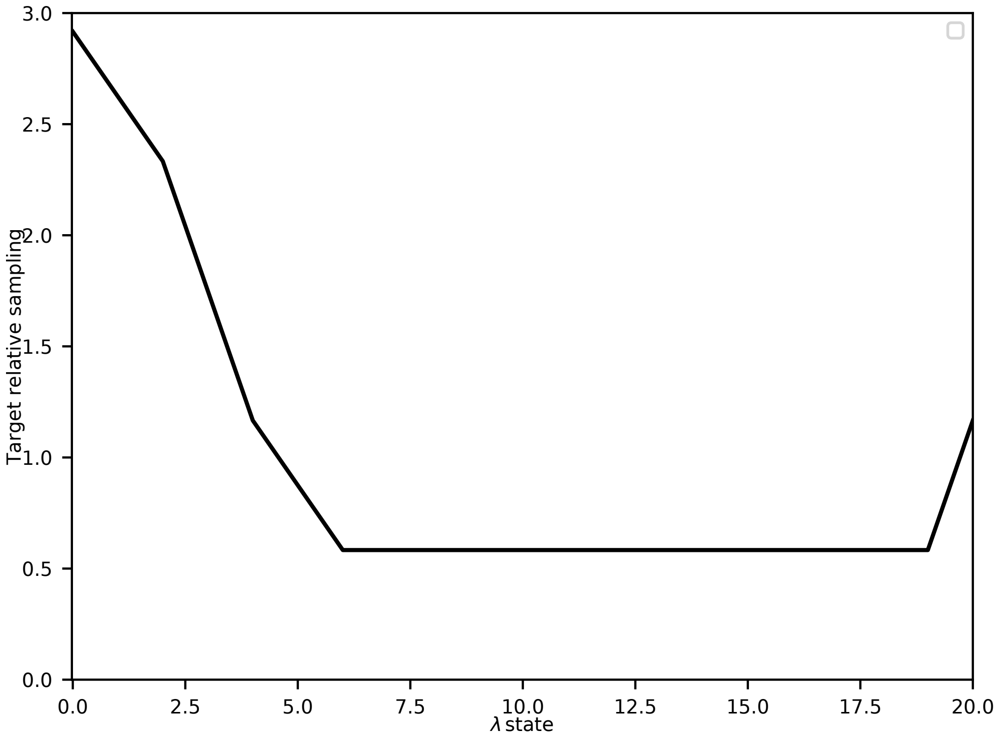

Figure S4: AWH target distribution along the alchemical free energy axis. λ state 0 was fully interacting and at λ state 20 there were no interactions between the molecule and its surroundings. Along the pull axis the target distribution was uniform.

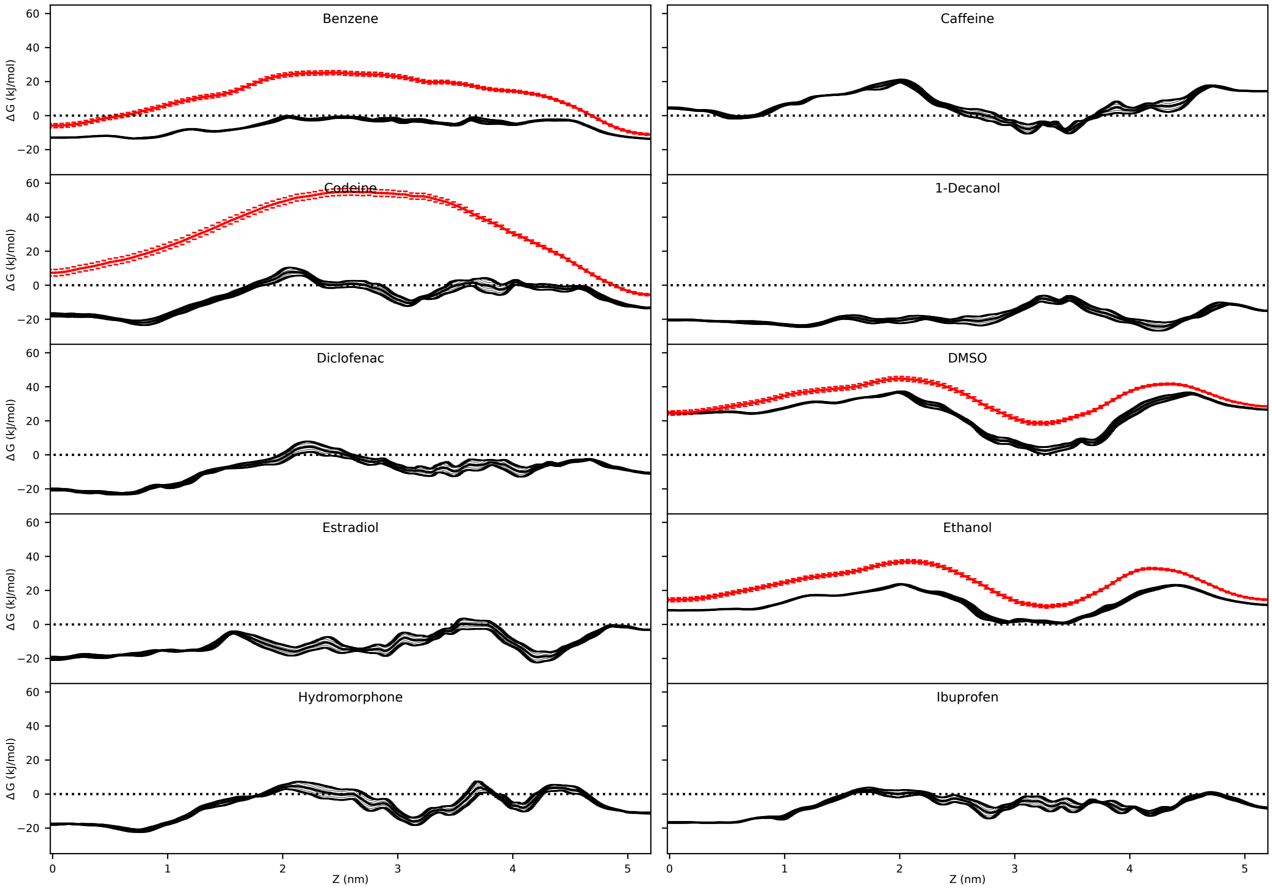

Figure S5: PMFs of 20 permeants. The PMFs are calibrated relative to the hydration free energies of the compounds. The profiles in black show the results from this study, whereas the profiles in red are from our previous FR pulling simulations (pulling at 0.2 nm ns^{-1}) (2). The curves in red were only calibrated based on the free energy difference, to the water solvent, at the ceramide sphingoid chain interface region (at ~5.2 nm). When calculating the permeability coefficients, the PMFs (in black) were shifted (up) so that their minima were ≥0 kJ mol^{-1}. I.e., the zero point was set to the lowest of the hydration free energy and the lowest point in the PMF. *Continued on next page.*

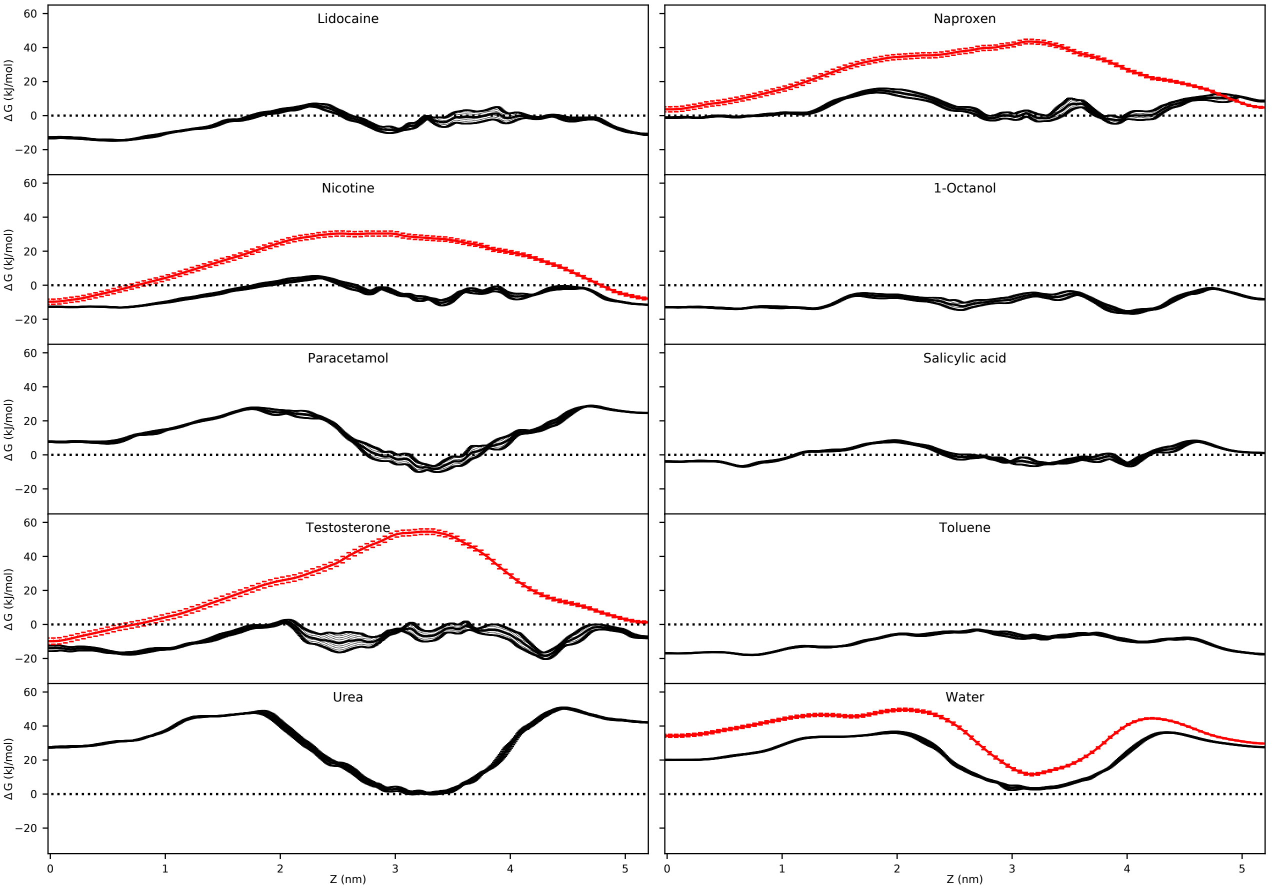

Figure S5: PMFs of 20 permeants. The PMFs are calibrated relative to the hydration free energies of the compounds. The profiles in black show the results from this study, whereas the profiles in red are from our previous FR pulling simulations (pulling at 0.2 nm ns^{-1}) (2). The curves in red were only calibrated based on the free energy difference, to the water solvent, at the ceramide sphingoid chain interface region (at ~5.2 nm). When calculating the permeability coefficients, the PMFs (in black) were shifted (up) so that their minima were ≥0 kJ mol^{-1}. I.e., the zero point was set to the lowest of the hydration free energy and the lowest point in the PMF. *Continued from previous page.*

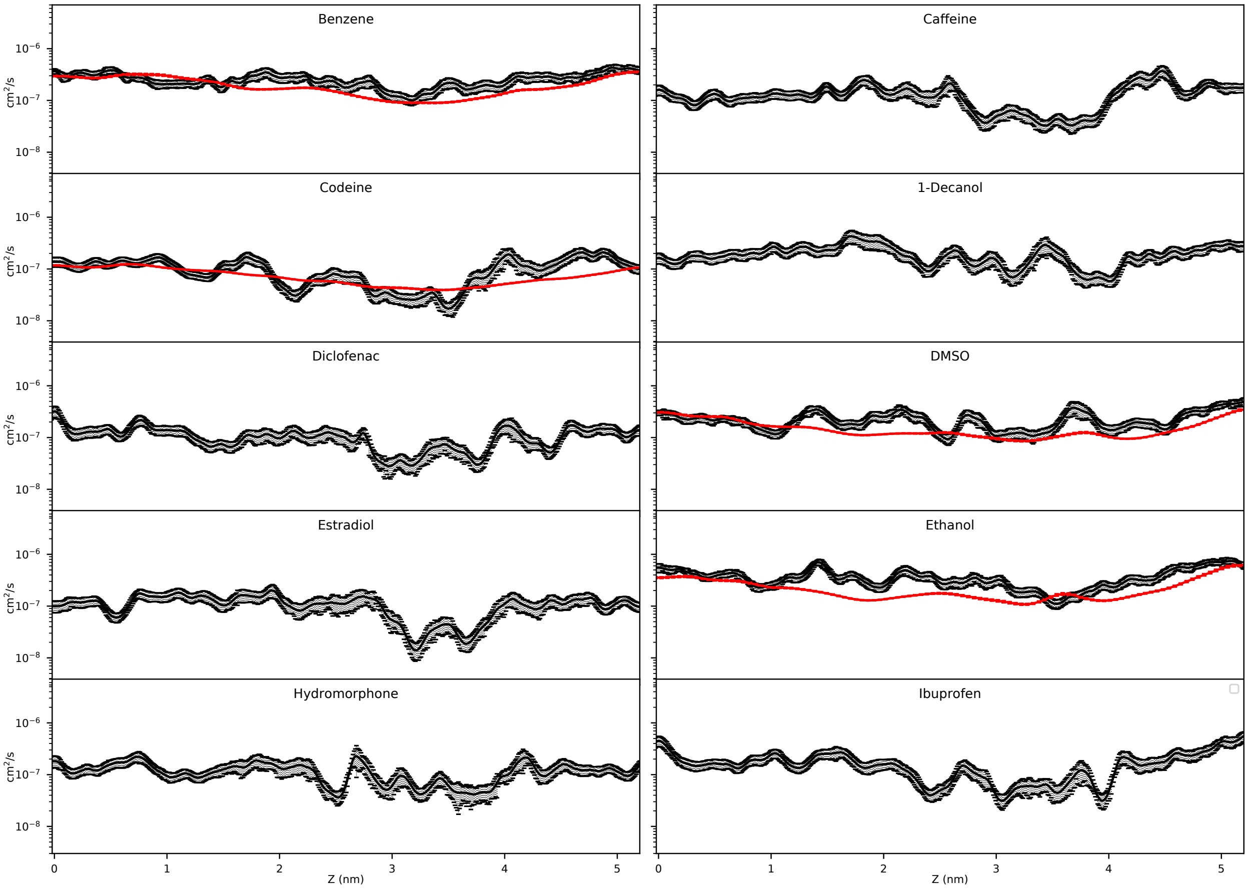

Figure S6: Diffusion coefficients of 20 permeants. The profiles in black show the results from this study, whereas the profiles in red are from our previous FR pulling simulations (pulling at 0.2 nm ns^{-1}) (2). The diffusion profiles from AWH were smoothened using a running median filter of 0.2 nm width, whereas a Hanning filter of 11 point (0.5 nm) width was used for the diffusion profiles from FR pulling. *Continued on next page.*

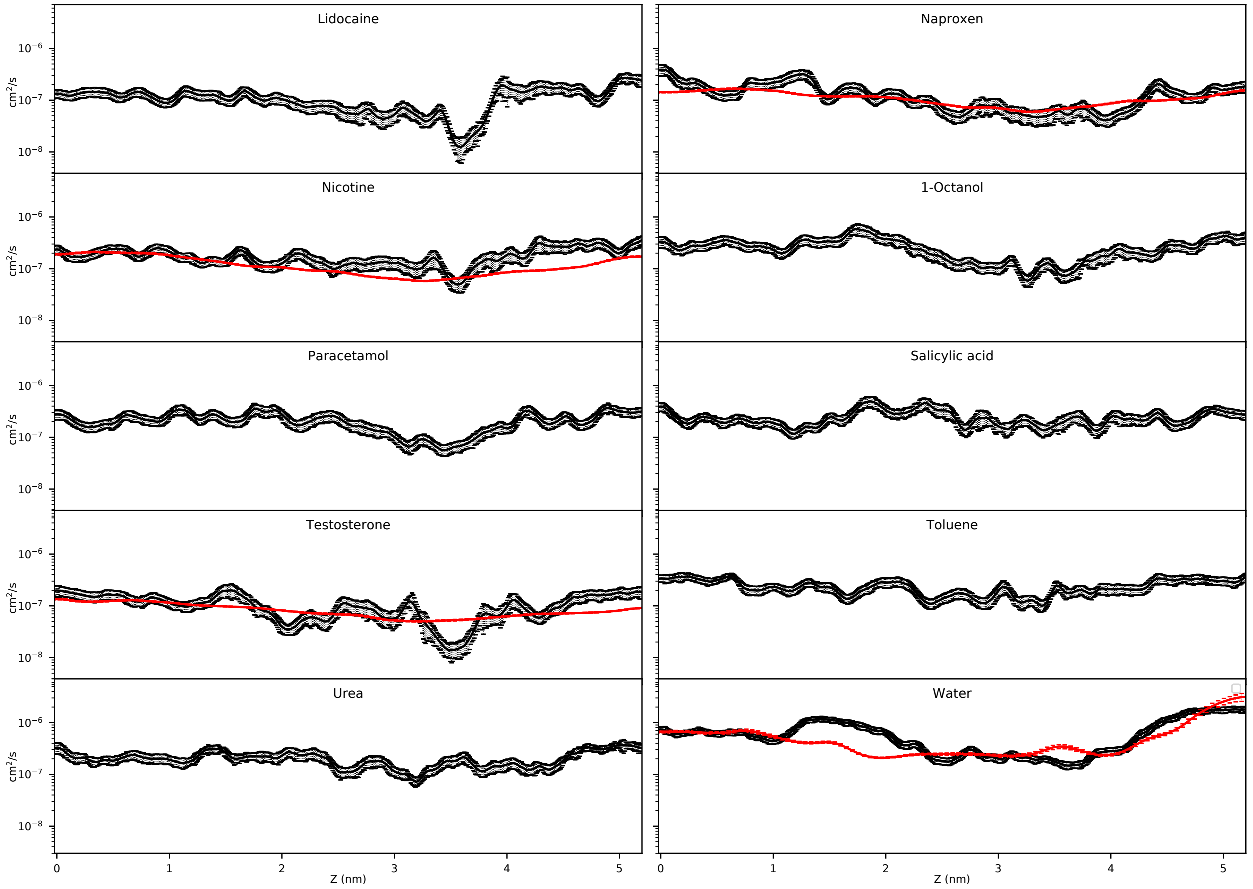

Figure S6: Diffusion coefficients of 20 permeants. The profiles in black show the results from this study, whereas the profiles in red are from our previous FR pulling simulations (pulling at 0.2 nm ns^{-1}) (2). The diffusion profiles from AWH were smoothened using a running median filter of 0.2 nm width, whereas a Hanning filter of 11 point (0.5 nm) width was used for the diffusion profiles from FR pulling. *Continued from previous page.*

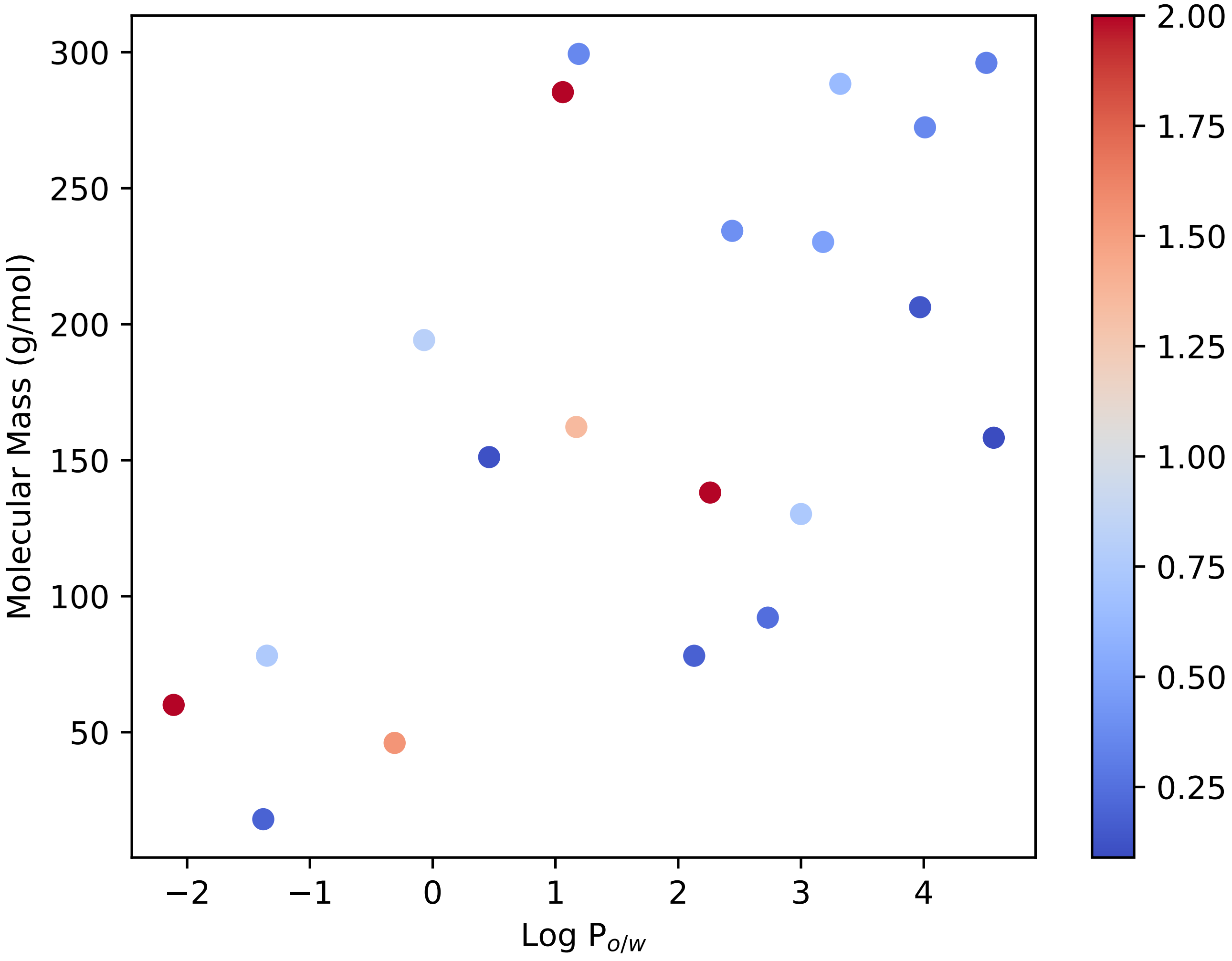

Figure S7: Molecular mass and log $P_{o/w}$ of permeants as well as their log K_P square difference (cm^2h^{-2}), between experimental data and calculation results, shown by the color scale.

Table S1: Permeability coefficients of unionized species at 32 °C, with corrections for temperature differences according to Abraham and Martins (3). If only observations from ionized species were found, corrections for pH/pK_a were then obtained from Abraham and Martins (3). The log $K_{P_{exp}}$ uncertainty is the standard error of the mean (SEM) of the measurements, if there were at least three reported measurements available. If there were only two experimental measurements the SEM was assumed to be 0.3 and shown in italics and if there was only one experimental measurement the SEM was reported as 0.4 (in italics). The calculated results are from 5 independent sets of simulations, each running 24 communicating AWH walkers for 450 ns.

**Permeant**	**log $K_{P_{exp}}$** (cm/h)	**log $K_{P_{calc}}$** (cm/h)
Benzene	−0.7 ± *0.3*[a]	−0.3 ± 0.1
Caffeine	−3.5 ± 0.2[b]	−2.6 ± 0.2
Codeine	−3.8 ± *0.4*[c]	−3.2 ± 0.4
1-Decanol	−0.8 ± *0.4*[d]	−0.5 ± 0.5
Diclofenac	−1.9 ± *0.4*[e]	−2.5 ± 0.3
DMSO	−3.1 ± *0.4*[f]	−4.0 ± 0.1
Estradiol	−2.4 ± 0.2[g]	−1.8 ± 0.4
Ethanol	−2.8 ± *0.3*[h]	−1.6 ± 0.1
Hydromorphone	−4.4 ± *0.4*[i]	−2.6 ± 0.3
Ibuprofen	−1.6 ± *0.4*[j]	−1.2 ± 0.1
Lidocaine	−2.2 ± *0.4*[k]	−1.6 ± 0.2
Naproxen	−1.6 ± *0.4*[l]	−0.9 ± 0.4
Nicotine	−2.0 ± *0.3*[m]	−0.8 ± 0.1
1-Octanol	−1.0 ± *0.4*[n]	−0.1 ± 0.2
Paracetamol	−4.3 ± *0.4*[o]	−4.0 ± 0.3
Salicylic acid	−1.7 ± *0.3*[p]	−0.3 ± 0.1
Testosterone	−2.5 ± 0.3[q]	−1.7 ± 0.4
Toluene	0.0 ± *0.4*[r]	−0.5 ± 0.1
Urea	−4.1 ± *0.3*[s]	−6.2 ± 0.1
Water	−3.1 ± 0.2[t]	−3.5 ± 0.1
Mean diff.		0.38
Mean abs. diff.		0.84
Mean sq. diff.		0.93[u]

[a] log $K_{P_{exp}}$ −0.80 cm h^{-1} at 25 °C (4) and −0.95 cm h^{-1} at 31 °C (5).
[b] log $K_{P_{exp}}$ −4.00 cm h^{-1} at 25 °C (4), −3.59 cm h^{-1} at 32 °C (6) and −3.19 cm h^{-1} at 32 °C (7).
[c] log $K_{P_{exp}}$ −4.31 cm h^{-1} at 37 °C and pH 7.4 (8) (pK_a 8.2).
[d] log $K_{P_{exp}}$ −1.10 cm h^{-1} at 25 °C (9).
[e] log $K_{P_{exp}}$ −1.74 cm h^{-1} at 37 °C, unionized (10).
[f] log $K_{P_{exp}}$ −3.10 cm h^{-1} at 5 % conc. at unspecified temperature (11). Cf., at 40 % conc. the log $K_{P_{exp}}$ was −1.80 cm h^{-1}.
[g] log $K_{P_{exp}}$ −2.01 cm h^{-1} at 37 °C (12), −2.28 cm h^{-1} at 30 °C (4), −2.34 cm h^{-1} at 34 °C (SC temperature) (13), −2.37 cm h^{-1} at 32 °C (14), −2.41 cm h^{-1} through full skin at 30 °C (15), −2.49 cm h^{-1} at 25 °C (4) and −3.52 cm h^{-1} at 26 °C (16).
[h] log $K_{P_{exp}}$ −3.14 cm h^{-1} at 25 °C (9) and −3.00 cm h^{-1} at 25 °C (17).
[i] log $K_{P_{exp}}$ −4.82 cm h^{-1} at 37 °C, pH 7.4 (8) (pK_a 8.2).
[j] log $K_{P_{exp}}$ −1.44 cm h^{-1} at 37 °C, presumably unionized (18).
[k] log $K_{P_{exp}}$ −2.15 cm h^{-1} at 32 °C, pH 10.0 (8) (pK_a 8.0).
[l] log $K_{P_{exp}}$ −1.42 cm h^{-1} at 37 °C, unionized (10).
[m] log $K_{P_{exp}}$ −1.71 cm h^{-1} at unknown temperature, unionized (19), −2.08 cm h^{-1} at 37 °C, unionized (20) .
[n] log $K_{P_{exp}}$ −1.28 cm h^{-1} at 25 °C (9).
[o] log $K_{P_{exp}}$ −4.30 cm h^{-1} at 32 °C (21).
[p] log $K_{P_{exp}}$ −1.52 cm h^{-1} at 37 °C, unionized (10) and −1.58 cm h^{-1} at 32 °C, pH 2.5 (22) (pK_a 3.0).
[q] log $K_{P_{exp}}$ −2.66 cm h^{-1} at 25 °C (4), −3.40 cm h^{-1} at 26 °C (16), −1.85 cm h^{-1} at 32 °C (23) and −3.07 cm h^{-1} at 25 °C (24).
[r] log $K_{P_{exp}}$ 0.0 cm h^{-1} at unknown temperature (25).
[s] log $K_{P_{exp}}$ −3.89 cm h^{-1} at 37 °C (26) and −4.04 cm h^{-1} at 34 °C (SC temperature) (13).
[t] log $K_{P_{exp}}$ −3.30 cm h^{-1} at 25 °C (9), −3.70 cm h^{-1} at 25 °C (9), −3.00 cm h^{-1} at 25 °C (17) and −3.40 cm h^{-1} at unspecified temperature (11).
[u] cm^2 h^{-2}

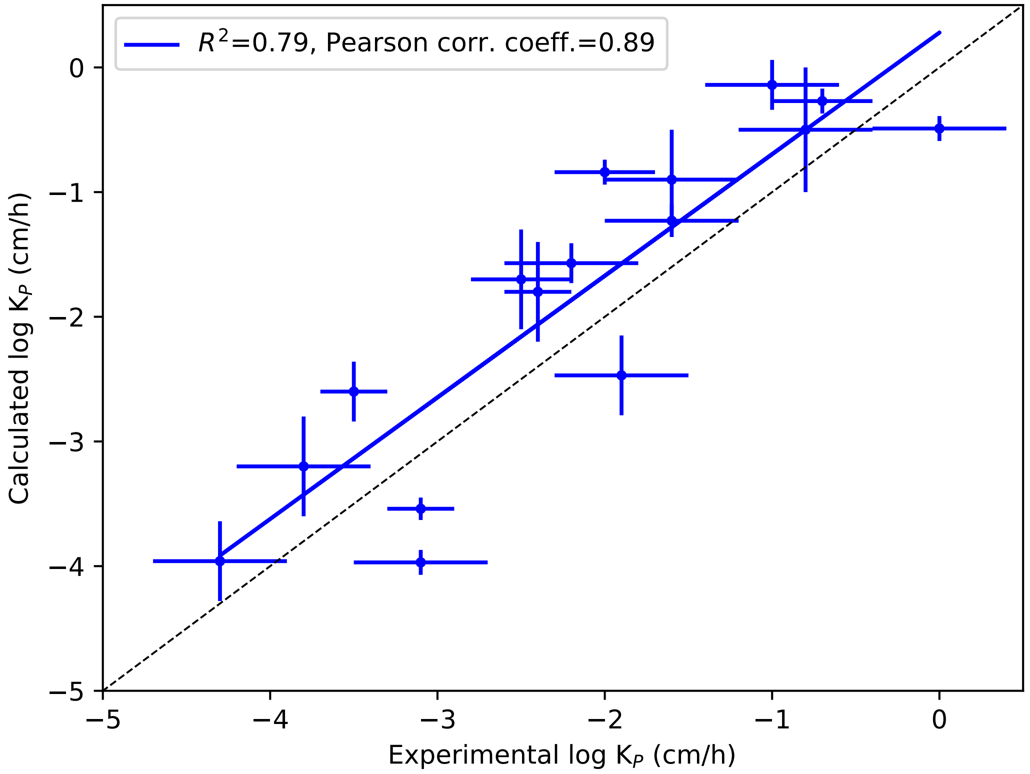

Figure S8: The relationship between experimental log K_P and those calculated from MD simulations, with the two largest outliers (urea and hydromorphone) excluded. The blue line shows the linear regression, whereas the dashed black line is the identity line. The error bars represent 1 SEM (standard error of the mean), which is approximated for experimental values. Details about the approximation is presented in the header of Table S1.

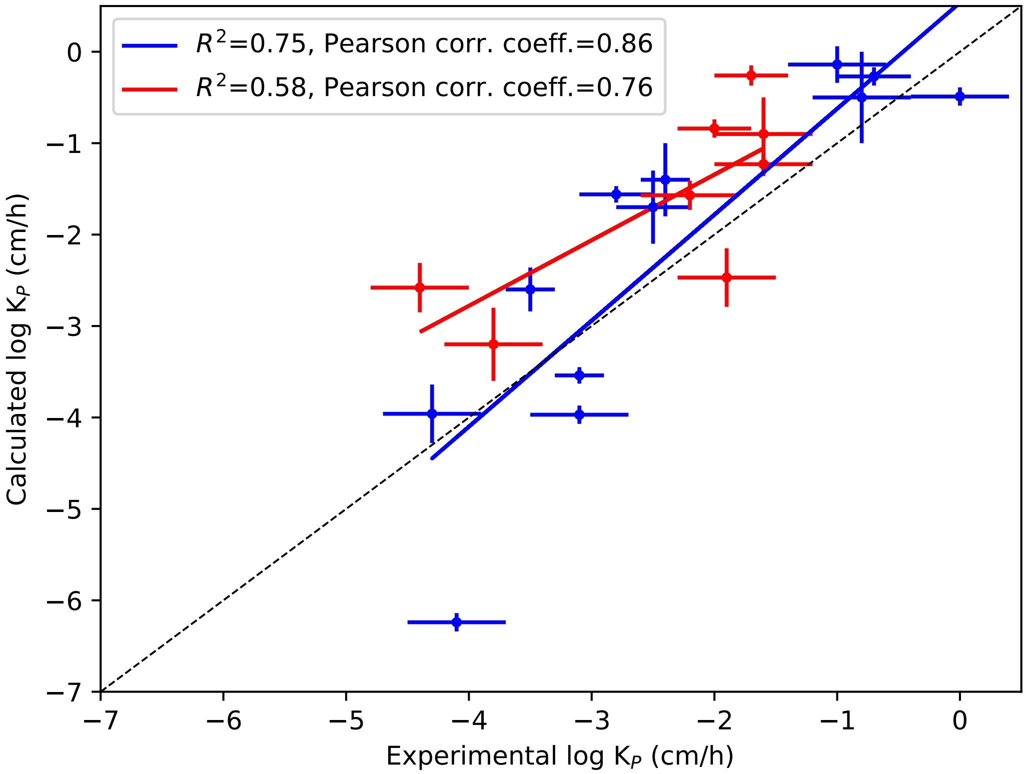

Figure S9: Separating molecules that are not ionizable (shown in blue) under the *ex vivo/in vitro* conditions from those that are ionizable (shown in red). The ionizable compounds are codeine, diclofenac, hydromorphone, ibuprofen, lidocaine, naproxen, nicotine and salicylic acid.

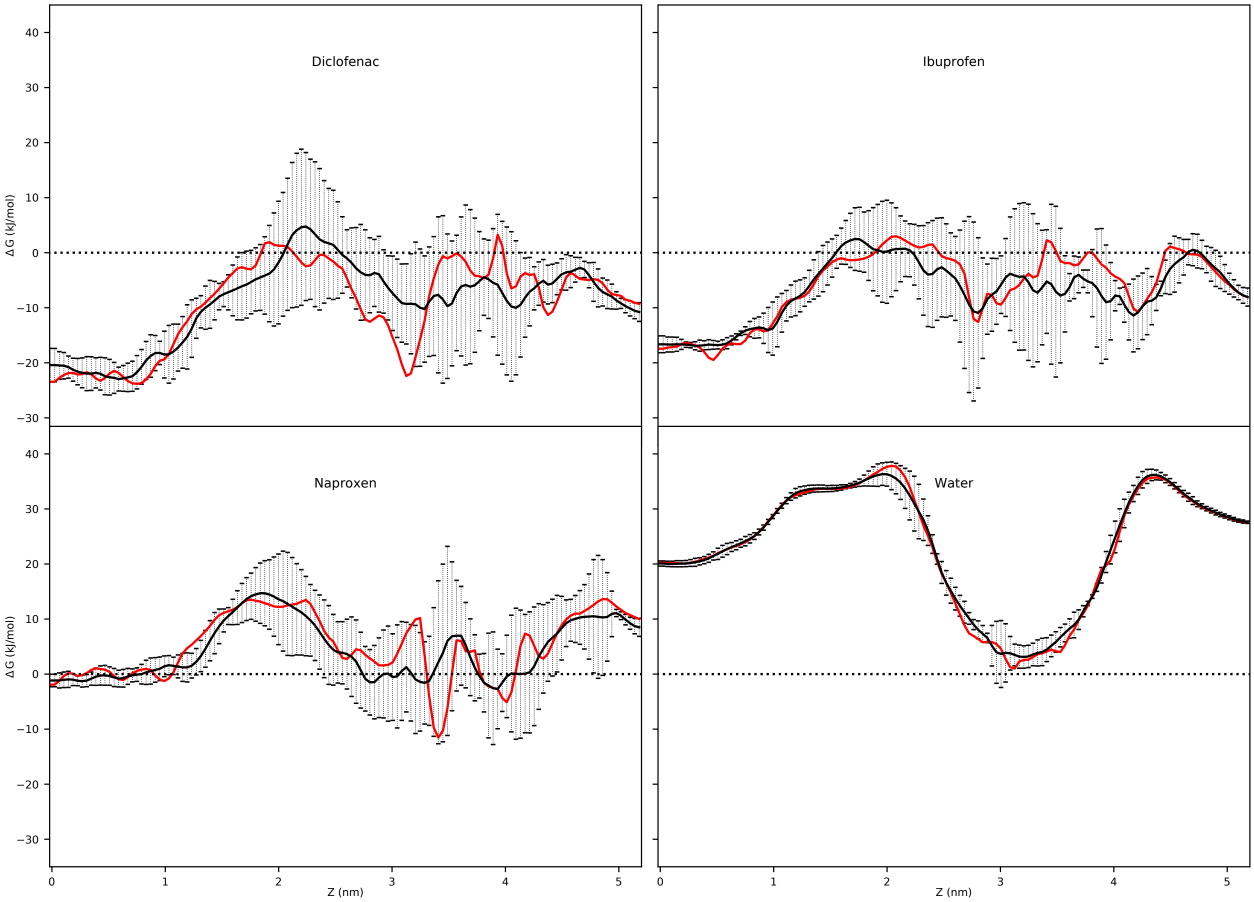

Figure S10: Comparing PMFs from simulations with 3 fs and 2 fs MD time steps. The black curves show the results from the five sets of simulations run with heavy hydrogen atoms and a 3 fs MD time step (cf. Fig.S5). The uncertainties here represent 2 STDs in order to allow a quick comparison to the red curves. The red curves show the results from one set of AWH simulations run with normal hydrogen mass and a 2 fs MD time step. Only every 4th data point is plotted in order to improve the clarity.

S2.4 Evaluating a 3 fs MD Time Step

The reported results are from simulations with a 3 fs MD iteration time step. That required increasing the mass of hydrogen atoms, by repartitioning mass from their bound heavy atom. In order to verify that this did not affect the results the PMFs and local diffusion coefficients of four molecules were calculated using normal hydrogen mass and a standard 2 fs MD iteration time step. The comparison is shown in Fig. S10 and S11. No significant differences were observed between the two alternative simulation parameter settings.

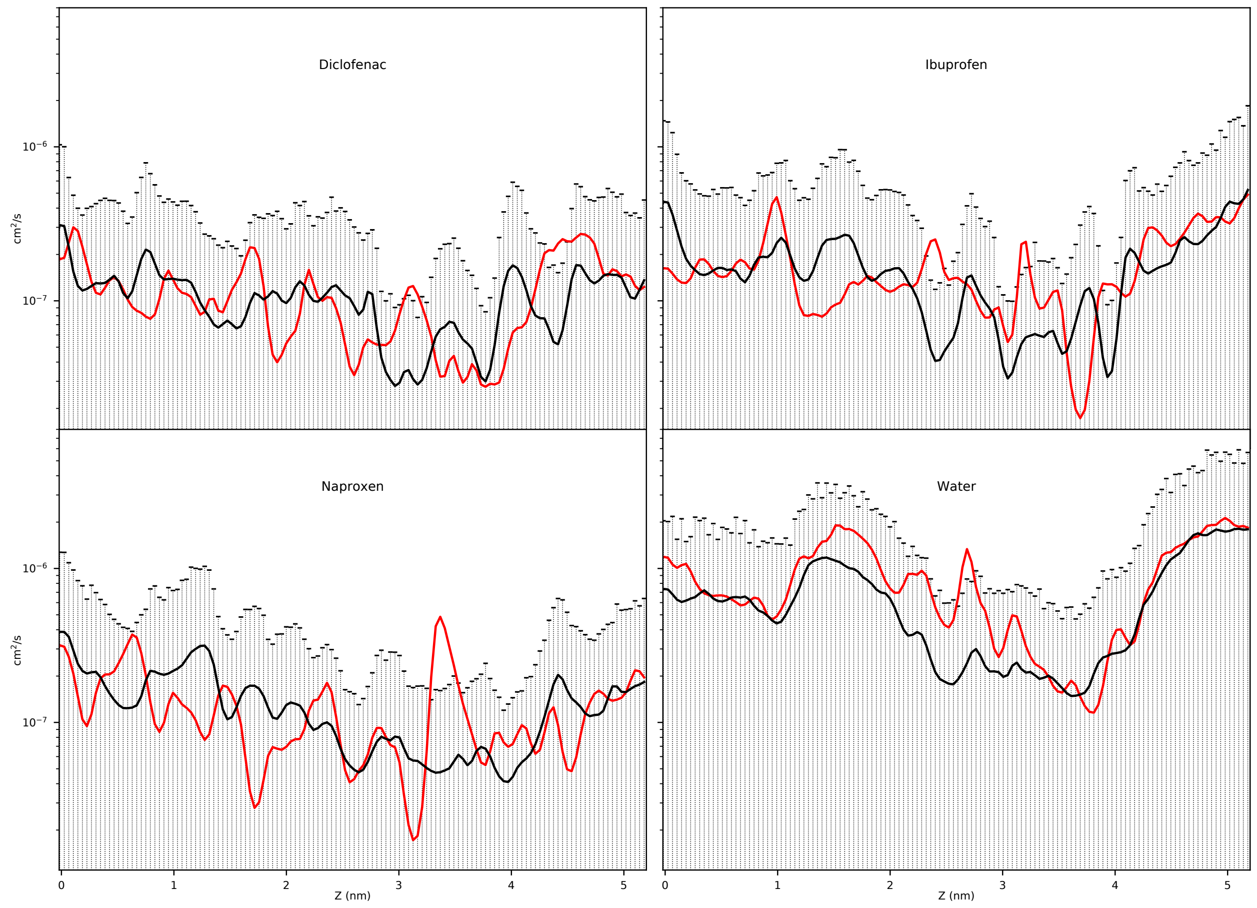

Figure S11: Comparing diffusion coefficients from simulations with 3 fs and 2 fs MD time steps. The black curves show the results from the five sets of simulations run with heavy hydrogen atoms and a 3 fs MD time step (cf. Fig.S6). The uncertainties here represent 2 STDs in order to allow a quick comparison to the red curves. The red curves show the results from one set of AWH simulations run with normal hydrogen mass and a 2 fs MD time step. Only every 4th data point is plotted in order to improve the clarity.

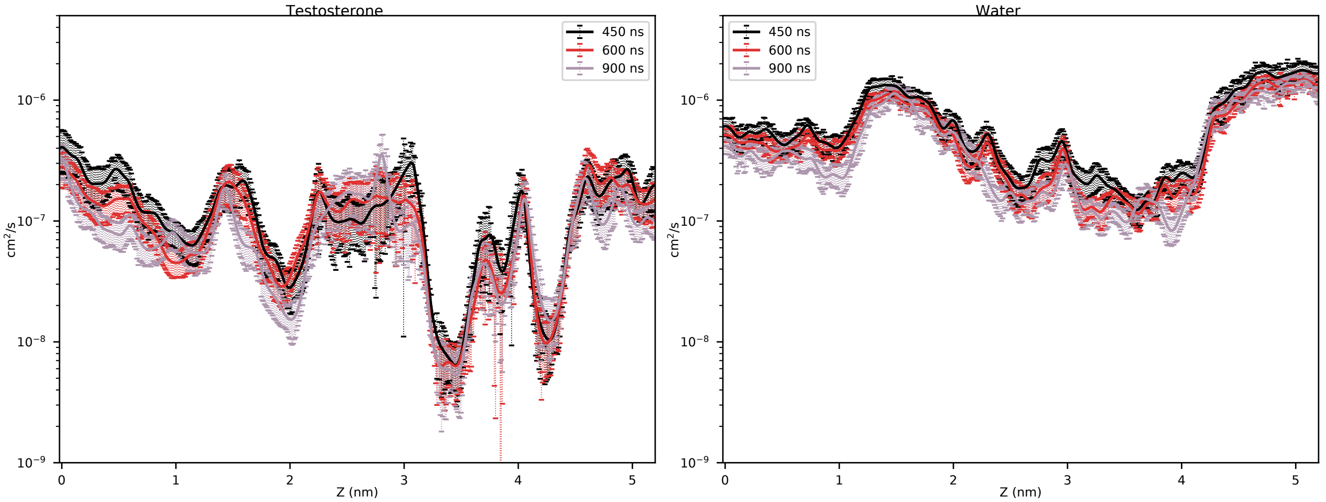

Figure S12: Calculated diffusion coefficients of testosterone and water from extended AWH simulations (24 communicating AWH walkers). The average diffusion coefficients through the system for testosterone were 1.3×10^{-7} cm^2 s^{-1}, 1.1×10^{-7} cm^2 s^{-1} and 8.8×10^{-8} cm^2 s^{-1} at 450 ns, 600 ns and 900 ns, respectively. For water they were 6.7×10^{-7} cm^2 s^{-1}, 5.5×10^{-7} cm^2 s^{-1} and 4.9×10^{-7} cm^2 s^{-1} at the same time points.

Table S2: Permeability coefficients of testosterone and water from extended AWH simulations. The AWH uncertainties from one single set of simulations (24 communicating AWH walkers) are rough estimates and are expected to be too low.

	**Simulation time (ns) per AWH walker (24 walkers)**		
**Permeant**	**450**	**600**	**900**
Testosterone	−2.9 ± 0.4	−2.8 ± 0.3	−2.8 ± 0.3
Water	−3.5 ± 0.2	−3.5 ± 0.2	−3.5 ± 0.2

S2.5 Convergence of Diffusion Coefficients

Fig. S12 shows how the calculated diffusion coefficients change with extended simulation times. Especially for testosterone it can be seen that longer simulation times result in lower diffusion coefficients. Table S2 shows the calculated permeability coefficients from the same simulations. Similar trends have been observed when lowering the pulling speed in FR simulations or increasing the sampling time in umbrella sampling simulations. A comparison of water diffusion coefficients from methods is shown in Fig. S13. The umbrella sampling results are from the same set of simulations, but the US 145-150 ns results discarded the first 145 ns from each simulation.

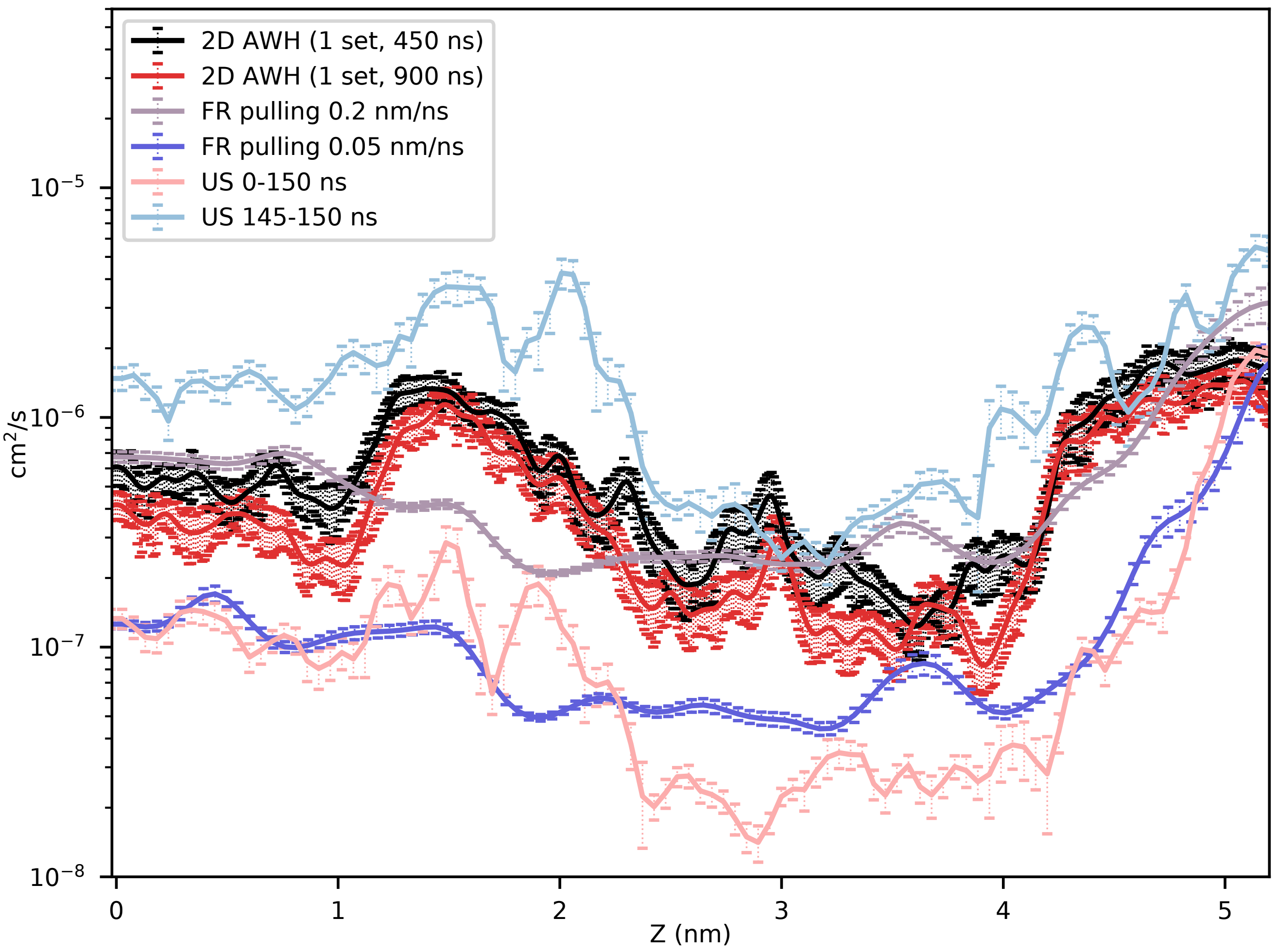

Figure S13: Calculated diffusion coefficients of water using 2D AWH, FR pulling and umbrella sampling. Longer sampling, or slower pulling, results in lower diffusion coefficients through the gel-like skin barrier structure. The AWH simulations results are from 1 set of 24 communicating AWH walkers, each running 450 or 900 ns for 11 and 22 µs, respectively. The 0.2 nm/ns FR pulling output is from 50 simulations pulling two molecules in each direction, for a total of 5 µs. The 0.05 nm/ns FR data is from 60 simulations pulling two molecules halfway across the system in each direction, for a total of 12 µs. The full set of umbrella sampling simulations is from 120 simulations, each with 4 water molecules, for a total of 18 µs for the full 0-150 ns data. The US 145-150 ns set only analyzed the last 5 ns from the same simulations. The diffusion profiles from AWH were smoothened using a running median filter of 0.2 nm width. A Hanning filter of 0.5 nm width was used for the diffusion profiles from FR pulling and 0.2 nm for umbrella sampling.

REFERENCES

1. Nategholeslam, M., C. G. Gray, and B. Tomberli, 2017. Stiff Spring Approximation Revisited: Inertial Effects in Nonequilibrium Trajectories. *J. Phys. Chem. B* 121:391–403. http://pubs.acs.org/doi/abs/10.1021/acs.jpcb.6b08701.

2. Lundborg, M., C. L. Wennberg, A. Narangifard, E. Lindahl, and L. Norlén, 2018. Predicting drug permeability through skin using molecular dynamics simulation. *J. Control. Release* 283:269–279. http://www.sciencedirect.com/science/article/pii/S016836591830292X.

3. Abraham, M. H., and F. Martins, 2004. Human skin permeation and partition: General linear free-energy relationship analyses. *J. Pharm. Sci.* 93:1508–1523. http://onlinelibrary.wiley.com/doi/10.1002/jps.20070/abstract.

4. Mitragotri, S., D. A. Edwards, D. Blankschtein, and R. Langer, 1995. A mechanistic study of ultrasonically-enhanced transdermal drug delivery. *J. Pharm. Sci.* 84:697–706. http://onlinelibrary.wiley.com/doi/10.1002/jps.2600840607/abstract.

5. Blank, I. H., and D. J. McAuliffe, 1985. Penetration of Benzene Through Human Skin. *J. Invest. Dermatol.* 85:522–526. http://www.sciencedirect.com/science/article/pii/S0022202X15438461.

6. Dias, M., A. Farinha, E. Faustino, J. Hadgraft, J. Pais, and C. Toscano, 1999. Topical delivery of caffeine from some commercial formulations. *Int. J. Pharm.* 182:41–47. http://www.sciencedirect.com/science/article/pii/S0378517399000678.

7. Uchida, T., W. R. Kadhum, S. Kanai, H. Todo, T. Oshizaka, and K. Sugibayashi, 2015. Prediction of skin permeation by chemical compounds using the artificial membrane, Strat-M™. *Eur. J. Pharm. Sci.* 67:113–118. http://www.sciencedirect.com/science/article/pii/S0928098714004163.

8. Roy, S. D., and G. L. Flynn, 1989. Transdermal Delivery of Narcotic Analgesics: Comparative Permeabilities of Narcotic Analgesics Through Human Cadaver Skin. *Pharm. Res.* 6:825–832. http://link.springer.com/article/10.1023/A:1015944018555.

9. Scheuplein, R. J., and I. H. Blank, 1973. Mechanism of Percutaneous Absorption. Iv. Penetration of Nonelectrolytes (alcohols) from Aqueous Solutions and from Pure Liquids. *J. Invest. Dermatol.* 60:286–296. http://www.nature.com.ezp.sub.su.se/jid/journal/v60/n5/abs/5617718a.html.

10. Singh, P., and M. S. Roberts, 1994. Skin permeability and local tissue concentrations of nonsteroidal anti-inflammatory drugs after topical application. *J. Pharmacol. Exp. Ther.* 268:144–151. https://jpet.aspetjournals.org/content/268/1/144.

11. Daugherty, J. E., 1997. Fundamentals of Receptor Assessment. *In* Assessment of Chemical Exposures: Calculation Methods for Environmental Professionals, CRC Press, 309.

12. Liu, P., W. I. Higuchi, A.-H. Ghanem, and W. R. Good, 1994. Transport of beta-Estradiol in Freshly Excised Human Skin in Vitro: Diffusion and Metabolism in Each Skin Layer. *Pharm. Res.* 11:1777–1784. https://doi.org/10.1023/A:1018975602818.

13. Intarakumhaeng, R., A. Wanasathop, and S. K. Li, 2018. Effects of solvents on skin absorption of nonvolatile lipophilic and polar solutes under finite dose conditions. *International Journal of Pharmaceutics* 536:405–413. https://linkinghub.elsevier.com/retrieve/pii/S0378517317310931.

14. Megrab, N. A., A. C. Williams, and B. W. Barry, 1995. Oestradiol permeation across human skin, silastic and snake skin membranes: The effects of ethanol/water co-solvent systems. *Int. J. Pharm.* 116:101–112. http://www.sciencedirect.com/science/article/pii/037851739400321U.

15. Galey, W. R., H. K. Lonsdale, and S. Nacht, 1976. The In Vitro Permeability Of Skin And Buccal Mucosa To Selected Drugs And Tritiated Water. *J. Invest. Dermatol.* 67:713–717. http://www.sciencedirect.com/science/article/pii/S0022202X15449334.

16. Scheuplein, R. J., I. H. Blank, G. J. Brauner, and D. J. Macfarlane, 1969. Percutaneous Absorption of Steroids. *J. Invest. Dermatol.* 52:63–70. http://www.sciencedirect.com/science/article/pii/S0022202X15476195.

17. Scheuplein, R. J., 1965. Mechanism of Percutaneous Adsorption. *J. Invest. Dermatol.* 45:334–346. http://www.sciencedirect.com/science/article/pii/S0022202X15470058.

18. Degim, I. T., W. J. Pugh, and J. Hadgraft, 1998. Skin permeability data: anomalous results. *Int. J. Pharm.* 170:129–133. http://www.sciencedirect.com/science/article/pii/S0378517398001136.

19. Flynn, G. L., 1990. Physicochemical determinants of skin absorption. *In* T. R. Gerrity, and C. J. Henry, editors, Principles of Route-to-route Extrapolation for Risk Assessment, Elsevier, New York, 93–127.

20. Zorin, S., F. Kuylenstierna, and H. Thulin, 1999. In vitro test of nicotine's permeability through human skin. Risk evaluation and safety aspects. *Ann. Occup. Hyg.* 43:405–413. https://doi.org/10.1093/annhyg/43.6.405.

21. Priprem, A., C. Khamlert, T. Pongjanyakul, S. Radapong, T. Rittirod, and P. Chitropas, 2008. Comparative Permeation Studies between Scale Region of Shed Snake Skin and Human Skin In vitro. *Am. J. Agricult. Biol. Sci.* https://agris.fao.org/agris-search/search.do?recordID=DJ2012079471.

22. Qvist, M. H., U. Hoeck, B. Kreilgaard, F. Madsen, and S. Frokjaer, 2000. Evaluation of Göttingen minipig skin for transdermal in vitro permeation studies. *European Journal of Pharmaceutical Sciences* 11:59–68. http://www.sciencedirect.com/science/article/pii/S0928098700000919.

23. An-eX Analytical Services Ltd, 2017. Assessment of the permeation of testosterone through human skin in vitro from saturated aqueous solution. Technical Report E05/02/17.

24. Siddiqui, O., M. S. Roberts, and A. E. Polack, 1989. Percutaneous absorption of steroids: relative contributions of epidermal penetration and dermal clearance. *J. Pharmacokinet. Biopharm.* 17:405–424.

25. Dutkiewicz, T., and H. Tyras, 1968. Skin absorption of toluene, styrene, and xylene by man. *Br. J. Ind. Med.* 25:243. https://www.ncbi.nlm.nih.gov/pmc/articles/PMC1008781/.

26. Peck, K. D., A. Ghanem, and W. I. Higuchi, 1994. Hindered Diffusion of Polar Molecules Through and Effective Pore Radii Estimates of Intact and Ethanol Treated Human Epidermal Membrane. *Pharm. Res.* 11:1306–1314. http://link.springer.com/10.1023/A:1018998529283.
