# Supplementary material for: Skin permeability prediction with MD simulation sampling spatial and alchemical reaction coordinates
Source: Biophys J. 2022 Sep 13;121(20):3837–49. doi: 10.1016/j.bpj.2022.09.009 (PMC9674988; doi:10.1016/j.bpj.2022.09.009)
Supplement: Document S2. Article plus supporting material [file mmc2.pdf]

Biophysical Journal
Article

Biophysical Society

Skin permeability prediction with MD simulation sampling spatial and alchemical reaction coordinates

Magnus Lundborg,[1,*] Christian Wennberg,[1] Jack Lidmar,[2] Berk Hess,[3] Erik Lindahl,[4,5] and Lars Norlén[6,7,*]
[1]ERCO Pharma AB, Science for Life Laboratory, Solna, Sweden; [2]Department of Physics, KTH Royal Institute of Technology, Stockholm, Sweden; [3]Department of Applied Physics, Science for Life Laboratory, KTH Royal Institute of Technology, Solna, Sweden; [4]Department of Biophysics and Biochemistry, Science for Life Laboratory, Stockholm University, Solna, Sweden; [5]Department of Physics, Swedish e-Science Research Center, KTH Royal Institute of Technology, Stockholm, Sweden; [6]Department of Cell and Molecular Biology (CMB), Karolinska Institutet, Stockholm, Sweden; and [7]Dermatology Clinic, Karolinska University Hospital, Stockholm, Sweden

ABSTRACT A molecular-level understanding of skin permeation may rationalize and streamline product development, and improve quality and control, of transdermal and topical drug delivery systems. It may also facilitate toxicity and safety assessment of cosmetics and skin care products. Here, we present new molecular dynamics simulation approaches that make it possible to efficiently sample the free energy and local diffusion coefficient across the skin's barrier structure to predict skin permeability and the effects of chemical penetration enhancers. In particular, we introduce a new approach to use two-dimensional reaction coordinates in the accelerated weight histogram method, where we combine sampling along spatial coordinates with an alchemical perturbation virtual coordinate. We present predicted properties for 20 permeants, and demonstrate how our approach improves correlation with ex vivo/in vitro skin permeation data. For the compounds included in this study, the obtained log $K_{Pexp\text{-}calc}$ mean square difference was 0.9 cm^2 h^{-2}.

SIGNIFICANCE Percutaneous drug administration allows for noninvasive, pain-free, continuous drug delivery with reduced side effects and increased patient compliance compared with per oral or intravenous drug administration. Permeability coefficients can be predicted using molecular dynamics simulations in silico with an atomistic model of the skin's barrier structure validated by cryo-EM in near-native skin. This can assist the interpretation of ex vivo/in vitro skin permeability data. The possibility to identify the locations of the most significant permeation barriers, as well as where permeation enhancers partition into the barrier structure, can help when designing transdermal, and topical, drug delivery systems. It may also be used for toxicity predictions of cosmetics or hazards in the work environment.
Here, we present the first use of the accelerated weight histogram method with a reaction coordinate combining a spatial dimension with an alchemical perturbation dimension. This yields a more efficient sampling of the free energy landscape and local diffusion coefficient of drugs passing through the skin's barrier structure. The two-dimensional sampling method can be applied to other molecular dynamics simulation systems with long correlation times.

INTRODUCTION

Transdermal and topical drug delivery allows for noninvasive, pain-free, continuous drug administration with reduced side effects and increased patient compliance compared with per oral or intravenous drug delivery.

Assessment of skin permeability is important for the design of percutaneous drug delivery systems. When designing a pharmaceutical dermal or transdermal formulation, also referred to as a drug delivery vehicle, it is often necessary to add chemical penetration enhancers to improve the uptake. Today, the dominating means of investigating drug skin permeability and the effects thereon of different delivery vehicle excipients is by ex vivo/in vitro testing. There are, however, ethical problems involved in the study of percutaneous drug delivery using excised human or animal skin, or using animals in vivo (1,2). There are also issues of the translation of animal drug delivery data to

Submitted March 15, 2022, and accepted for publication September 8, 2022.
*Correspondence: magnus.lundborg@ercopharma.com or lars.norlen@ki.se
Editor: Alan Grossfield.
https://doi.org/10.1016/j.bpj.2022.09.009
© 2022 Biophysical Society.
This is an open access article under the CC BY license (http://creativecommons.org/licenses/by/4.0/).

humans (1–3). When using human skin, there are complications of inter- and intraindividual variation as well as of interlaboratory differences (1,4). Furthermore, ex vivo/in vitro testing yields no information about how compounds permeate skin.

In silico modeling is an interesting alternative to predict skin permeability and to gain insights into how compounds are absorbed in skin (5), but this requires both faithful models of the skin system and methods that can sample the process efficiently. A skin permeation prediction model relevant for human skin, encompassing the effects of penetration enhancers and other drug delivery vehicle excipients, could help improve transdermal formulation design.

We have previously shown that an atomistic model of the skin's barrier structure, i.e., the intercellular lipid matrix of the stratum corneum, validated against cryoelectron microscopy (cryo-EM) data from near-native skin (6) (Fig. 1), could be used in molecular dynamics (MD) simulations to predict the permeability of molecules through skin (7). These models made it possible to reproduce the effects of chemical penetration enhancers on the skin's barrier structure at least qualitatively (7). This enabled a molecular-level understanding of their mechanisms of action, but the amount of sampling required and limited convergence made it difficult to accurately match experimental values. Improving the sampling efficiency for this type of complex system has led to a number of improvements of simulation protocols (8). We show better sampling of the free energy landscape and local diffusion coefficient of drugs passing through the skin's barrier structure.

Here, we apply the accelerated weight histogram (AWH) method, using a two-dimensional (2D) reaction coordinate. This combines a spatial dimension, pulling the molecule across the barrier structure, with a second alchemical free energy dimension (8). It is expected that such sampling, allowing decoupling of the permeant, will be efficient in systems with long correlation times (9), such as the skin's barrier structure. An improved agreement between calculated and measured permeability coefficients is presented. It is shown that, using the AWH method, ex vivo/in vitro measured skin permeability data can be accurately predicted, even though the in silico model selectively accounts for the skin's barrier structure and not for the complete skin.

This study includes 20 chemical compounds with molar masses ranging from 18 to ~300 g mol^{-1} and $\log P_{o/w}$ from −2.1 to 4.6. The distribution of these two molecular properties can be seen in Fig. S7. The experimentally measured permeability coefficients, $\log K_P$, of the molecules vary from 0.0 to −4.4 cm h^{-1}. In addition to drug compounds, the studied permeants also encompass excipients, such as DMSO, ethanol, urea, and water. Octanol and decanol are not used as excipients, but were chosen to represent longer fatty alcohols. Those are commonly used as permeation enhancers, but do not have publicly available permeability coefficients. Benzene and toluene were included to verify that high-permeability coefficients can be correctly predicted. Transdermal, and topical, drugs have a wide spread of permeability coefficients, but their $\log K_P$ values are commonly found between −2.0 and −4.5 cm h^{-1}. The permeability coefficient on its own cannot determine whether a drug is suitable for transdermal delivery or not. The required therapeutic concentration, solubility in the delivery vehicle as well as possible permeation enhancing techniques must also be taken into account.

The organization of the lipid barrier in human stratum corneum has received much attention for almost half a century. It has long been known that the lipids are organized as stacked layers (10,11). Important to highlight are models proposed by Swartzendruber et al. (12), Bouwstra et al. (13), Hill and Wertz (14), McIntosh (15), and Schröter et al. (16). In 2012, Iwai et al. (17) proposed a model based on CEMOVIS (cryoelectron microscopy of vitreous sections) images, of volar forearm and abdomen skin samples from five individuals, and simulation of EM images (18) from basic modeling of molecular arrangements. That structure proposal, as well as the experimental data and methods, served as the starting point for the model used herein (6), which in turn fits with a recent explanation of the skin

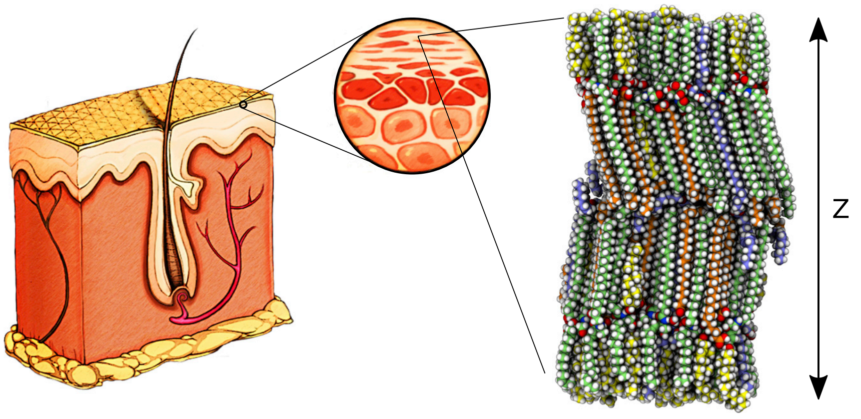

FIGURE 1 A schematic representation of the structure of epidermis and dermis. To the right is shown a snapshot of the atomistic model of the intercellular lipid structure of human stratum corneum that constitutes the skin's main permeability barrier (6). The arrow indicates the main permeability direction, also referred to as the *Z* dimension. In the MD simulations the system is in practice repeated infinitely in the three dimensions. The carbon atoms are colored based on the molecule type, where ceramides are green, acyl ceramides (ceramide EOS) are light blue, free fatty acids are orange, and cholesterols are yellow. Hydrogen, nitrogen, and oxygen atoms are colored white, blue, and red, respectively. To see this figure in color, go online.

barrier's formation process based on cryo-EM (19). Another recently proposed model is the one by Mojumdar et al. (20), based on neutron diffraction studies of model lipid mixtures. Different experimental techniques, and sample treatment, have resulted in different observed periodicities of the lipid structure. Repeating units of primarily 4.5, 6.5 (or 4.5 + 6.5 nm), 11, and 13 nm have been reported in the studies mentioned above.

MD skin permeation modeling

Quantitative structure-permeability relationship (QSPR) methods (21–25) are commonly used to predict skin permeability coefficients. QSPR models have proven very good at predicting permeability coefficients with reported mean square error as low as 0.48 $cm^2\ h^{-2}$ (log units) and R^2 of 0.7 (22,25). MD simulations constitute an alternative or complement to QSPR methods. MD simulations are significantly slower than QSPR methods, but not as limited to a specific applicability domain. Furthermore, incorporating the effect of formulation excipients on the permeability of active pharmaceutical ingredients (APIs) requires retraining of QSPR models, preferably for each combination of excipients. There have, nevertheless, been QSAR (quantitative structure-activity relationship) (26) studies involving chemical permeation enhancers, but information about these excipients' mechanisms of action has been difficult to obtain. With MD simulations, formulation excipients can be incorporated in the skin's barrier structure in their most probable concentrations, locations, and orientations, based on their local free energy potentials. This enables studying how they interact with the barrier and how they affect the permeability of one or more APIs. MD simulations cannot be expected to compete with QSPR models for pure permeability coefficient predictions, but they can provide important additional understanding. However, the first step must be to verify that the permeability coefficients calculated from MD simulations agree with experimental data.

One limitation of the MD simulation approach presented here is that only the structure of the main permeability barrier in skin (27,28), and not the complete skin, is studied in detail. However, it would be possible to complement the calculated permeability coefficients with mathematical models accounting for corneocyte permeability as well as for diffusion through the viable epidermis and dermis (29–31).

There are several groups that have studied permeation through skin using MD simulations prior to this. Das et al. (32) calculated the permeability coefficient of water, through one single CER:CHOL:FFA 2:2:1 hairpin bilayer in water at 300 K and reported log K_P −4.9 cm h^{-1}. The permeability through a CER:CHOL:FFA 1:1:1 bilayer was only studied at 350 K and the log K_P was −3.5 cm h^{-1}. Gupta et al. (33) calculated the permeability of 12 compounds through a fully hydrated CER:CHOL:FFA 1:1:1 hairpin bilayer. Ten of the compounds had experimental measurements. The molecular masses ranged from 18 to 106 g mol^{-1} and log $P_{o/w}$ from −2.1 to 3.3. Their obtained mean square error was ∼13.8 $cm^2\ h^{-2}$. The effect of lateral diffusion on water permeability through a model membrane was studied by Del Regno and Notman (34). They also used multiple levels of hydration in their work. Wang and Klauda (35) used a more complex structure, based on the work of Mojumdar et al. (20), involving two types of ceramides, in addition to ceramide EOS. They calculated the permeability coefficient of ethanol in good agreement with experimental results. MacDermaid et al. (36) employed another method of calculating the permeability coefficient, through a hairpin system including ceramide EOS. Their calculations involved fitting the diffusion pathway length constant using regression. Thereby they obtained a fit of the curve that was very good (log K_P mean square error ∼0.5 $cm^2\ h^{-2}$) for nine molecules, with molecular masses <300 g mol^{-1} and log $P_{o/w}$ ranging from −3.1 to 3.7.

In our previous studies we showed that it is possible to predict the permeability coefficient of different chemical compounds through an atomistic model of the skin's barrier structure (6,7), illustrated in Fig. 1. Therein we used MD simulations to pull the permeants through the barrier system using a stiff spring and the forward-reverse (FR) method to calculate the permeability coefficients (37–40). The lipid barrier model was developed by optimizing the structure based on the fit of simulated cryo-EM images, after MD equilibration, to CEMOVIS images. The model consists of fully splayed ceramides, free fatty acids, and cholesterol, as well as ceramide EOS and a low amount of water, located in the ceramide head group region (6).

Based on our previous proof-of-concept study (7) we have studied the effects of the pulling speed on the calculated permeability coefficients in more detail. In the supporting material (Fig. S1) we illustrate sampling problems using potentials of mean force (PMFs), i.e., the relative free energy difference across the system. In these examples we show testosterone—a permeant that we have experienced to need careful sampling to obtain accurate results. The general observation was that, with slower pulling speeds, the PMFs grew more detailed and the resulting free energy barriers became lower. We attribute this to the skin's barrier lipid system being in a gel-like state and that the permeant needs to be pulled slowly to remain in a state near equilibrium, even if FR pulling is a nonequilibrium method (39). The experience that slower pulling speeds resulted in lower PMFs correlated in time with the publication by Wang and Klauda (35), in which they stated that 80 (approximately) stacked lipid bilayers of the skin's barrier structure must be taken into account (29,35,41), since the calculated permeability coefficient is not actually an average speed through the system, even if its unit would indicate that. The effect is that the permeability coefficient should be divided by 80, or 1.9 be subtracted from its log value. When taking 80 stacked lipid bilayers layers into

account for calculating the permeability coefficients using the data from our previous study (7) the log $K_{Pexp\text{-}calc}$ mean square difference was approximately ~11 cm^2 h^{-2} (cf. ~3 cm^2 h^{-2} in the original work (7)). This led us to conclude that very slow pulling speeds might be required to obtain correct PMFs. This would, in turn, require very long simulation times for each pulling simulation (see Figs. S1 and S3)—potentially so long that entirely different sampling approaches are required to predict permeability with reasonable amounts of computing time. Since it is not the main topic of this paper we refer to the supporting material for more discussions about FR pulling and umbrella simulations.

A permeant's skin permeability coefficient is mainly determined by the height of the peaks of its PMF across the skin's barrier structure, assuming that the passage through the barrier structure is the rate limiting step for diffusion. On the other hand, the partitioning of a permeant from a formulation into the skin's barrier structure primarily depends on the depth of the troughs of the permeant's PMF. Therefore, it is important that the MD simulations can reproduce the whole PMF as accurately as possible, given the available computational resources. The calculated PMF of codeine (the *black curve* in the codeine subfigure in Fig. S5) serves as an example. The tight lipid packing in the ceramide fatty acid chain region ($Z \sim 2.1$ nm in the PMF) constitutes the main permeation barrier. Closer to the fatty acid tail ends ($Z \sim 0.8$ nm in the PMF) there is a combination of nonpolar interactions with the loosely packed lipid chains and hydrophilic interactions with the ceramide EOS ester moiety. These favorable interactions trap the permeant in that region. To improve the permeability of codeine we would propose to choose a permeation enhancer acting in the ceramide fatty acid chain region, rather than in the sphingoid chains. This information would not be accessible if the PMF sampling was poor (see the *red curve* for codeine in Fig. S5) or, presumably, if there were no ceramide EOS in the lipid barrier model structure.

Skin permeability calculations with the AWH method

The AWH method (42,43) is an extended ensemble technique, in which an adaptive bias is used to flatten the free energy landscape. The applied bias enables sampling high free energy (low probability) configurations to the same extent as low free energy states. It is also possible to customize the target sample distribution to focus more on regions of the free energy landscape that are of higher interest.

In this work, we have used the AWH method to improve the sampling of the permeability of 20 permeants through the skin's barrier system. Since GROMACS 2021 it is possible to use a 2D AWH sampling with an alchemical free energy dimension (8) combined with a spatial dimension, i.e., pulling the permeant across the skin's barrier system. The alchemical free energy calculations imply that the interactions of the permeant with its surroundings can be gradually decoupled (switched on or off), thereby estimating the free energy of insertion. This means that there is no need for separate calibration of the PMFs in relation to the hydration free energy, or solvation free energy in the delivery vehicle of choice, as all points along the PMF will be calibrated compared with a vacuum state, in the same way as the solvation free energies are calculated. This also improves the sampling in regions with very slow diffusion (long correlation times), since turning off the interactions with the surroundings will let the permeant leave that region to sample other parts of the landscape, and later return in a different configuration (9). Fig. 2 shows a general overview of the method applied to calculating the PMF of testosterone through the skin's barrier structure. With the AWH method it is also trivial to extend the simulations until they are sufficiently converged. This solves the primary sampling problem from previous studies, and it also enables us to calculate the local diffusion coefficient based on the AWH friction metric along the spatial dimension. The free energy of solvation in the solvent, or formulation, should still be calculated, which can be done in a separate 1D alchemical AWH simulation (8).

Alchemical transformations in a gel-phase system

It is widely accepted that it is more efficient to perform electrostatic and Lennard-Jones transformations (coupling/decoupling) in sequence to ensure that the Coulomb interactions are switched off before Lennard-Jones interactions are and vice versa (44). This avoids overlap of atoms when the attractive Coulomb forces are strong and the repulsive Lennard-Jones forces are turned off or are very weak. However, the skin's barrier structure is in a gel-like state with very low mobility. We have found that the optimal orientation of a permeant molecule might be completely different in a state with its Lennard-Jones interactions turned on and Coulomb interactions turned off, compared with in its fully interacting state, and that the interconversion between the orientations can take very long when the Lennard-Jones interactions are turned on. For example, consider inserting (turning on interactions of) 1-decanol in the sphingoid chains of the skin's barrier structure. If first turning on Lennard-Jones interactions, the probability of inserting the molecule with the hydroxyl group oriented away from the head group region, into the interface between the lipid chains, will be approximately the same as inserting it in its most favorable position with the hydroxyl group oriented toward the headgroup region. When turning on the Coulomb interactions it will be unfavourable if the molecule is oriented in the opposite direction and it will take very long for it to turn around. If turning on the Lennard-Jones and the

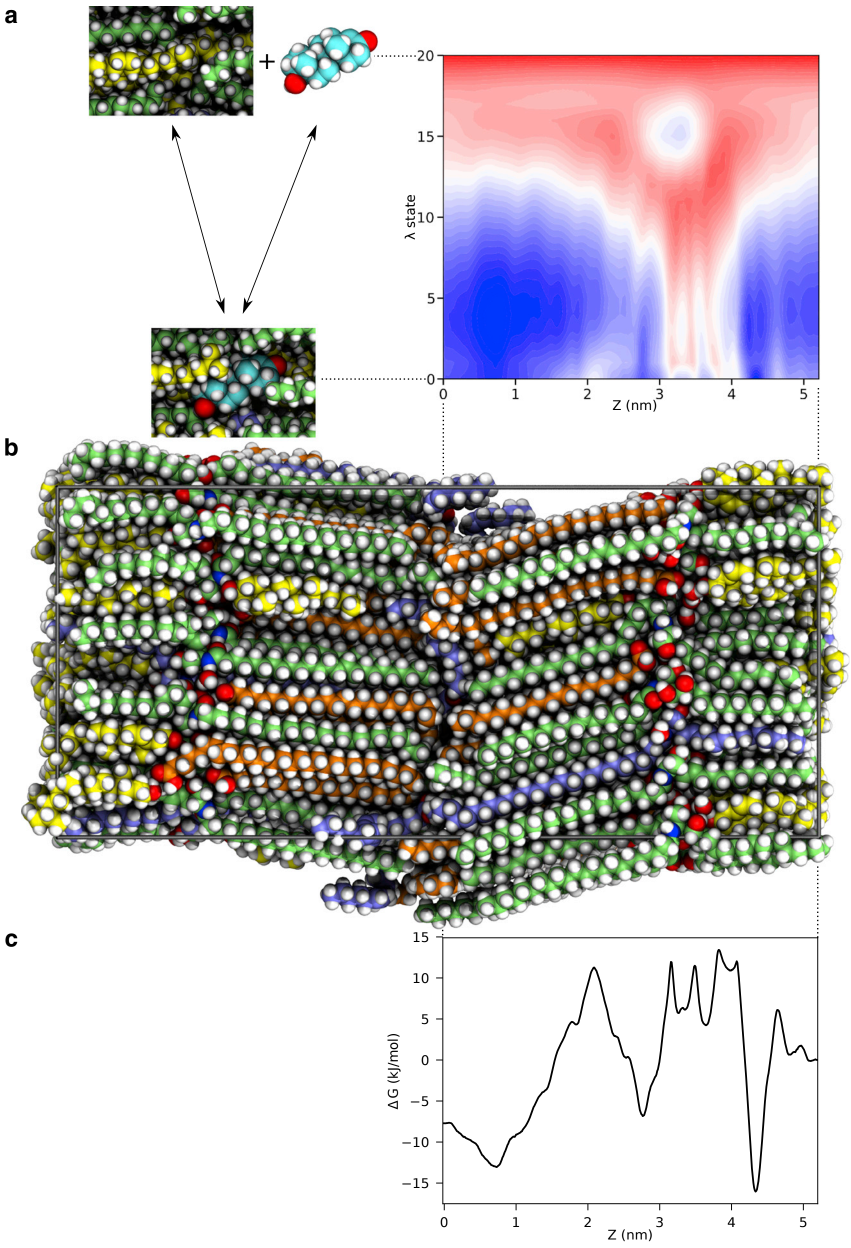

FIGURE 2 Calculating the PMF of testosterone through the skin's barrier structure. (*a*) On the right, the 2D free energy landscape of testosterone where the alchemical free energy λ state is shown on the y axis—0 is fully interacting and 20 is fully decoupled (as illustrated by the miniatures to the left of the free energy landscape). On the x axis the z coordinate (in nm) through the simulated system is shown. (*b*) The skin's barrier system, aligned to illustrate how the PMF is mirrored around 0 (the middle of the system). (*c*) The PMF of testosterone through the skin's barrier structure of the fully interacting λ state (0 on the y axis in *a*). The PMF is from one set of simulations of 24 communicating AWH walkers, for a total of 11 μs. AWH analyses do not give a reliable error estimate from one set of simulations, therefore there are no error bars presented in this plot. For error estimations based on multiple simulations see Fig. 3. To see this figure in color, go online.

Coulomb interactions simultaneously the electrostatic interactions will favor inserting the molecule correctly from the start.

In this project we decided to use the same, simultaneous interaction transformation, settings for both the 2D alchemical free energy/spatial AWH simulations as well as for the 1D AWH alchemical hydration free energy calculations, even if the efficiency might not be optimal for the latter. It should be noted that this problem is at least partially avoided if the alchemical free energy simulations are started from a fully interacting and equilibrated state or, as mentioned above, in a system with fast diffusion in all dimensions.

Number of lipid bilayers to consider

Looking more closely at the published data it can be found that the assumed number of 80 bilayers (35,41) may need revision. Skin samples for skin permeability measurements are usually from the abdominal regions or the chest. Ya-Xian et al. (45) reported the number of cell layers in samples from the trunk, i.e., shoulder, chest, back, abdomen, and buttock, to be 13 on average, with a standard error of the mean (mean $\pm$ SE) of 0.4. This was the same source that was used to motivate choosing 15 layers, from a range of 6–20 (29,41). The intercellular lipids are reported to be located between the corneocytes, resulting in 12 ± 0.4

intercellular regions of lipids, each consisting of a number of lipid bilayers. Since the structure of lipid layers above and below the bottom- and topmost corneocytes have not been clearly distinguished with CEMOVIS, these are not included in the number of lipid layers we take into account. These could possibly add to the total permeation resistance, but the difference would be small. Wang et al. (29) reported a range of three to nine bilayers between corneocytes, without a source, and choosing six bilayers as a reasonable average. Elias and Friend (11) have shown that there are commonly three to five continuous sheets between adjacent corneocytes, with an intercellular space ranging from 60 to 100 nm in neonatal mouse skin. From CEMOVIS studies of human skin a range of one to five bilayers between cells are commonly observed (17,46). After further discussion concerning observations from human skin we decided to settle upon an average of 2.5 intercellular bilayers with an approximate mean $\pm$ SE of 0.5.(47) This results in an average number of total bilayers of 30 ± 6 (with the mean $\pm$ SE as uncertainty), based on $12 \pm 0.4 \times 2.5 \pm 0.5$. This revised number of layers has been used in our calculations.

METHODS

The atomistic model of the human skin's barrier structure presented by Lundborg et al. (6), and originally called 33/33/33/75/5/0.3 (Relative composition in: molar % ceramides/molar % cholesterol/molar % free fatty acids/relative amount of cholesterol on ceramide sphingoid side/molar % acyl ceramide EOS (included in the relative ceramide concentration)/water molecules per lipid (not included in the molar % concentrations of the lipids)), was used as the starting structure for the simulations. The starting model had been equilibrated for approximately 270 ns, 250 ns of which were without restraints (6).

All MD simulations in this study (not including the data presented in Figs. S1 and S2) were performed using GROMACS 2021 and the second beta version of GROMACS 2022 (48–50), using the AWH method for alchemical free energy calculations (8,42,43). A source code modification enabled symmetrizing AWH sampling along a spatial (pulling) reaction coordinate dimension and also changed the number of AWH blocks for autocorrelation analysis from 64 to 128. These changes are available from the GROMACS gitlab repository (51).

van der Waals interactions had a cutoff of 1.2 nm with a smooth force-switch from 1.0 to 1.2 nm. The simulations were run without a dispersion correction for energy and pressure. Coulomb interactions were calculated using PME (52) with a radius of 1.2 nm. Bonds to hydrogen atoms were constrained using the P-LINCS algorithm (53,54). TIP3P (55) parameters were used for water molecules. For the lipid molecules, the CHARMM36 lipid force field (56,57) was used. Ceramide parameters were modified to more accurately reproduce the ceramide NP crystal structure (58), as described in (6). To allow a 3 fs integration time step, hydrogen atoms were made three times heavier by repartitioning the corresponding mass from their bound heavy atoms. Temperature was set to 305.15 K by using a stochastic dynamics integrator (also referred to as a velocity Langevin dynamics integrator) with a time step of 3 fs and with a time constant τ of 2 ps (corresponding to a friction constant of 0.5 ps^{-1}). The pressure was set to 1 atm and controlled using a stochastic cell rescaling barostat (59) with a time constant of 1.0 ps and a compressibility of 4.5×10^{-5} bar^{-1}. In the skin's barrier system the pressure coupling was semi-isotropic with no compressibility in the Z dimension. During the development of the calculation methods, for all permeants, at least one simulation was performed with unmodified hydrogen masses and a 2 fs time step to verify that the longer (3 fs) time step did not affect the results. However, most of those 2 fs time step simulations were run with other parameters. To verify that there were no significant differences, with the same simulation settings as in the simulations used for producing the permeation results, four permeants were re-run with standard hydrogen masses and 2 fs MD simulation time step. As can be seen in Figs. S10 and S11 there were no large differences in the PMFs and diffusion coefficients.

Along the alchemical free energy dimension, 21 equidistantly distributed λ states were used for decoupling both van der Waals and Coulomb interactions simultaneously. There were 10 or 100 steps between each Monte Carlo coordinate sampling, along the alchemical free energy λ-value and spatial position across the lipid structure. There were 10 samples per update of the f_λ bias. There was no observable difference between sampling every 10 or 100 steps, but the simulations were faster when sampling less frequently. Soft-core transformations (60) with $\alpha = 0.5$ and $\sigma = 0.3$ nm were applied to both the van der Waals and Coulomb interactions of the solute.

Topologies, i.e., inter- and intramolecular interaction parameters, for all permeants and formulation components except water, were generated using STaGE (61), which in turn uses Open Babel (62) and MATCH (63) to generate GROMACS topologies compatible with the CGenFF (64) CHARMM force field.

AWH simulations can be performed using multiple walkers. This means that the simulations are run in parallel and that the sampling is communicated between them when the bias and PMF are updated. That means that they share the same bias and effectively share the sampling of the free energy landscape. Since the bias and PMF are shared between the walkers multiple independent sets of simulations need to be performed to estimate the uncertainty. However, currently the AWH friction metric is not communicated. This means that the diffusion coefficient is calculated separately for each AWH walker and that it is possible to estimate an uncertainty of the diffusion coefficient from one set of AWH simulations with multiple walkers. Each AWH walker samples both reaction coordinate dimensions, but it is not necessary that each individual walker samples all points in the 2D free energy landscape.

All images representing molecules were prepared using Tachyon (65) in VMD (66).

Hydration free energy calculations

The hydration free energy was calculated starting with a water box large enough to solvate the molecule. The molecule was inserted into the system at a random position with its interactions to the surroundings turned off, as if in vacuum. AWH was used to sample the alchemical free energy λ states (8) to calculate the solvation free energy. The initial AWH histogram size, which determines the initial update size of the free energy, was set indirectly by specifying an estimate of the diffusion constant along the alchemical free energy λ-axis in combination with a rough estimate of the initial error. An input diffusion constant of 1×10^{-3} ps^{-1} was used for the hydration free energy calculations, which means that it is estimated to take approximately 1 ns to cross the alchemical dimension for one AWH walker. The initial error was set to 10 kJ mol^{-1}. Sixteen communicating AWH walkers were run in parallel, with the requirement that only simulations that covered the whole alchemical reaction coordinate counted toward the covering check in the initial AWH stage. The simulations were 60 ns long per walker for a total simulation time of 960 ns per solute. For a more detailed description of alchemical hydration free energy calculations using AWH see Lundborg et al. (8).

Permeability coefficient calculations

The permeability coefficient, K_P, and the permeation resistance, R, were calculated as follows (67):

$$\frac{1}{K_P} = R = \int_{Z_1}^{Z_2} \frac{e^{\beta \Delta G(z)}}{D(z)} dz, \tag{1}$$

where ΔG is the difference in free energy compared with the reference state, and D is the local diffusion coefficient across the skin's barrier structure (dz).

As mentioned above, we calculated the permeability through 30 ± 6 layers of the lipid barrier. The resulting permeability coefficient calculation was:

$$\frac{1}{K_P} = R = 30 \pm 6 \times \int_{Z_1}^{Z_2} \frac{e^{\beta \Delta G_{rel.water}(z)}}{D(z)} dz, \qquad (2)$$

where $\Delta G_{rel.water}$ is the free energy relative to the hydration free energy. When calculating the permeability coefficients, an additive constant of each PMF was chosen so that the PMF was never below 0, similar to what Wang and Klauda (35), as well as Venable et al. (68), have done before.

The local diffusion coefficient $D(z)$ across the barrier is estimated from the friction metric $g(z)$ calculated in the AWH simulation (69), via an Einstein relation $D(z) = g^{-1}(z)$. This rests on the assumption that the motion of the permeant may be approximated as a slow Markovian process, a condition that appears to be well satisfied in the present application. The AWH friction metric is calculated using the autocorrelation of the bias force from the whole simulation, including the AWH initial stage (69). We have verified that effects on the friction metric, from the larger bias fluctuations in the initial stage, are small. The diffusion coefficient from a shorter testosterone simulation with a constant bias, using the bias from a 900 ns simulation as input, was evaluated. It was similar to output at corresponding times from the simulations with adaptive bias in the regions sufficiently sampled. The diffusion coefficient converged toward the results from the longer simulation. To reduce the noise of the local diffusion coefficient curves, an 0.2 nm wide rolling median filter was applied. When symmetrizing the sampling, along the spatial dimension, by using the absolute coordinate values and accounting for the AWH bias across the sampling boundaries there are usually artificial spikes at the edges of the PMFs. These were removed by setting the two lowest (0.005 and 0.015 nm) and highest (5.200 and 5.210 nm) points in the PMF to the value of their neighbors (0.025 and 5.19 nm, respectively). These minor adjustments had no effects on the calculated permeability coefficients.

At the start of each AWH walker simulation the permeating molecule was inserted in the lipid barrier structure at a random position with all interactions to its environment turned off. The free energy profile through the skin's barrier structure was calculated using a 2D AWH setup, using a harmonic potential to steer the permeant across the system, also referred to as the Z dimension, and an alchemical free energy reaction coordinate (8). This allows sampling the free energy along the permeation direction and also the relative insertion free energy of the permeant from vacuum. In turn, this enables a direct calibration to the hydration free energy, since the vacuum state is the same in both cases. As when calculating the hydration free energy, the estimated AWH initial error was set to 10 kJ mol^{-1}. The AWH input diffusion constant was set to 3×10^{-5} nm^2 ps^{-1} for the spatial pulling dimension and 5×10^{-5} ps^{-1} along the alchemical free energy dimension. The input diffusion constant affects only the AWH histogram size, it does not affect the computed diffusion coefficient, which is obtained from the AWH friction metric during data analysis. The AWH force constant along the spatial Z dimension (normal to the lamellar stack), steering the permeant relative to the ceramide fatty acid chains using a harmonic pull potential, was set to 25,000 kJ mol^{-1} nm^{-2}. The force constant also determines the resolution along the reaction coordinate dimension. For each permeant, five sets of simulations were run with heavy hydrogen atoms (see above) and a 3 fs integration time step. These were run using 24 communicating walkers, each running for 450 ns. The covering check in the initial AWH stage only took into account simulations that covered the whole alchemical dimension and at least a diameter of 0.8 nm along the spatial dimension. From these simulations a combined diffusion coefficient was calculated using the AWH friction metric from all contributing walkers. The combined PMF was derived from the average of the independent PMFs from the five sets of simulations. The combined outputs were used to calculate the permeability coefficients presented in Table S1.

Along the alchemical free energy dimension it is the end states, i.e., the fully interacting and fully decoupled states, that are of highest interest, as the difference in free energy between them corresponds to the probability of transferring the permeant from vacuum into the skin's barrier structure. Therefore, the target distribution used in these simulations put more weight on the end states, especially the state with interactions fully turned on. The target distribution along the alchemical free energy dimension that was used is shown in Fig. S4. Along the spatial pulling dimension the target distribution was uniform.

One-dimensional AWH simulations of testosterone across the barrier structure were also performed for evaluation and comparison with the 2D AWH results. They were run using 4 independent sets of simulations, each using 24 communicating AWH walkers running for 600 ns. The simulation settings were the same as specified above, except that there was no alchemical free energy dimension.

RESULTS

Permeability coefficient calculations

Running 1D AWH simulations, with only a spatial reaction coordinate dimension across the barrier structure, did not sample the free energy profile of testosterone efficiently. None of four sets of simulations, each consisting of 24 communicating walkers sharing the same AWH bias and running for 600 ns for a total simulation time of 14.4 μs in each of the four independent simulation sets, sampled enough to cover the reaction coordinate with the condition that a single walker must cover a diameter of 0.8 nm to be taken into account. It is possible that this requirement was too strict for a system with inherently slow diffusion. This meant that the 1D AWH simulations did not leave the AWH histogram equilibration stage as part of the AWH initial stage. Increasing the AWH input diffusion constant would have increased the bias more quickly, meaning that covering of the reaction coordinate would have been quicker. However, that might also have resulted in too high free energy barriers in the PMF. The average PMF from the four sets of 1D AWH simulations is compared with the results from the 2D AWH simulations, including an alchemical dimension, in Fig. 3. The results from four sets, of totally 58 μs, of 1D AWH were worse than after 11 μs of 2D AWH with an alchemical reaction coordinate; i.e., assuming that the five sets (55 μs) of 2D AWH simulations are closest to convergence. The PMF output from 1D AWH were fairly similar to the FR pulling simulations shown in Fig. S3, but with higher uncertainty, i.e., a larger difference between the four sets of simulations.

The results from the 2D AWH simulations agreed well with the experimental data, thanks to the improved sampling of the free energy landscape. The relationship between calculated and experimental permeability coefficients are presented in Fig. 4. The numerical results are presented in Table S1. The largest outlier was urea, followed by hydromorphone with log K_P deviations of −2.1 and 1.8 cm h^{-1}, respectively. The calculated permeability coefficients

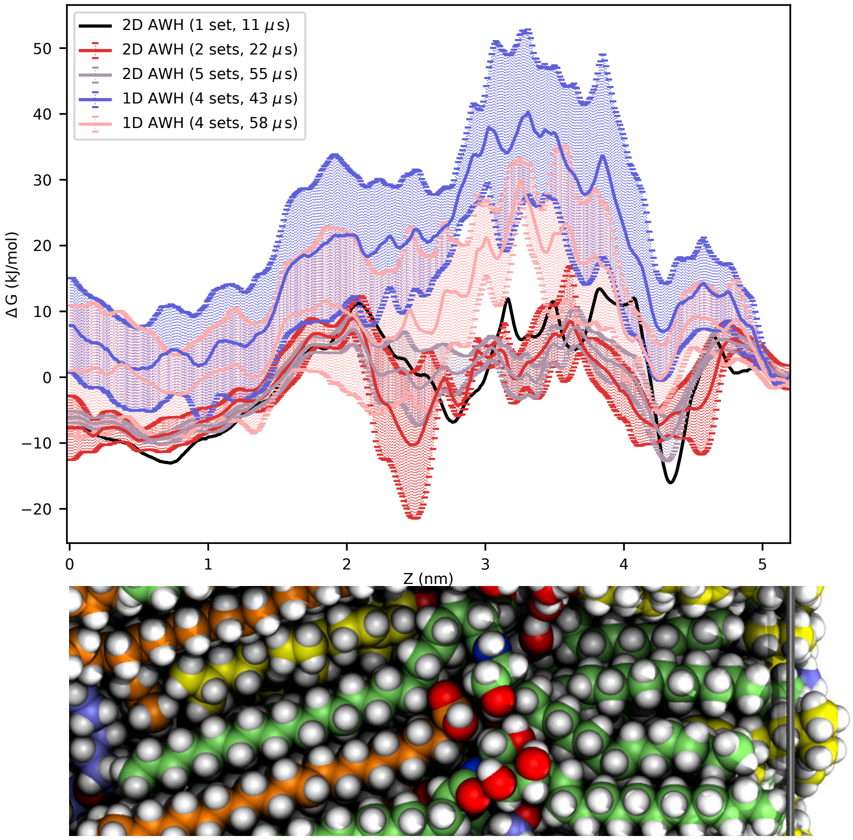

FIGURE 3 Faster convergence with 2D AWH. The PMFs of testosterone are from 2D spatial/alchemical AWH (in the fully interacting alchemical λ state) and 1D AWH with only a spatial reaction coordinate dimension. In both cases the PMFs are symmetrized and only half the PMFs are presented, and the PMFs are calibrated to 0 at the ceramide sphingoid chain interface (5.2 nm) to make comparisons easier. The uncertainties represent one standard error of the mean between the independent sets of simulations. The 11 μs 2D AWH results are from one set of 24 communicating walkers. AWH analyses do not give a reliable error estimate from one set of simulations, therefore there is no error presented for the 11 μs AWH plot (in black). The 1D AWH simulations of 58 μs required approximately the same computation time as 22 μs of 2D AWH. A snapshot of the molecular system is shown below the plot to indicate where the head groups are located (at $\approx$ 3.1–3.3 nm). To see this figure in color, go online.

tended to be overestimated, on average by 0.38 log units. The average absolute log K_P deviation was 0.84 cm h^{-1} (see Table S1). If excluding urea and hydromorphone it would be reduced to 0.71 cm h^{-1}.

In the supporting material (Figs. S5 and S6), the PMFs and local diffusion coefficients across the skin's barrier structure are shown. There are also comparisons to our previously published results (7) using FR pulling. Fig. S7 shows the molecular mass and log $P_{o/w}$ properties of the permeants as well as the differences between calculated and experimental log K_P. No obvious clusters with high errors can be observed.

For permeability coefficient calculations, all simulations in this study were run in 5 independent sets of 24 communicating AWH walkers, each running for 450 ns. Our observations are that the calculated permeability coefficients are largely unaffected when increasing the simulation time by a factor two, from 450 to 900 ns per AWH walker (see Table S2 and Figs S12 and S13).

In general, the resulting PMFs from 2D AWH are more stable, when extending the total simulation time, than those from 1D AWH and FR pulling. When simulating for longer periods, the calculated PMFs from 1D AWH and FR pulling are observed to approach those from 2D AWH.

Convergence of diffusion coefficients and PMFs

We have observed that the calculated diffusion coefficients depend on the simulation time, with lower diffusion coefficients the longer the simulation time. In general, the same trend is also observed regarding the PMF, i.e., lower free energy barriers with more sampling. We have seen this using all MD simulation sampling methods mentioned herein: AWH (1D with a spatial dimension and 2D with an added alchemical free energy perturbation dimension), umbrella sampling, and FR pulling. Examples of the effect on the calculated water diffusion coefficient from different methods and simulation settings are shown in Fig. S13.

DISCUSSION

When comparing PMFs of testosterone from the 2D AWH simulations performed in this study to previously performed (unpublished) FR pulling simulations (Fig. S3), it is clear that the PMFs from the 30 μs pulling simulations are closer to the PMFs from AWH. The calculated log K_P for testosterone from the FR pulling simulations (taking the 30 stacked lipid bilayers into account) was −3.8 cm h^{-1}, compared with −1.7 cm h^{-1} from the AWH simulations and −2.5 cm h^{-1} for the ex vivo/in vitro measurements (with reports ranging from −1.9 to −3.2 cm h^{-1} (70–73), corrected to a temperature of 305 K).

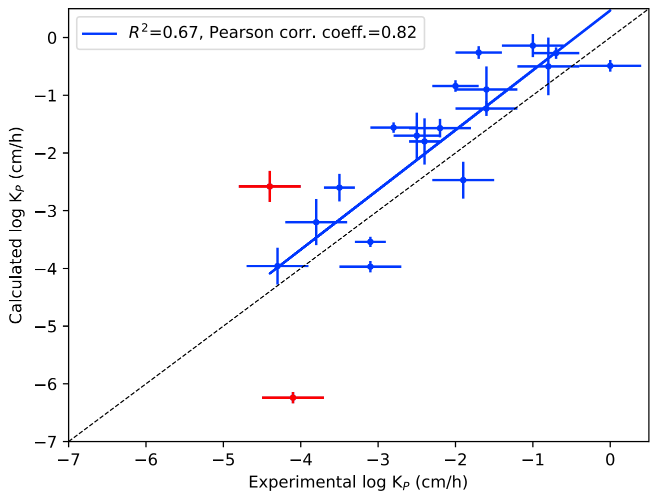

FIGURE 4 The correlation between experimental log K_P and those calculated from MD simulations. The blue line shows the linear regression, whereas the dashed black line is the identity line. The two largest outliers, urea and hydromorphone, are marked in red (still included in the linear regression). The error bars represent 1 mean ± SE (standard error of the mean), which is approximated for experimental values. Details about the approximation is presented in the header of Table S1. To see this figure in color, go online.

The 2D AWH sampling, with one alchemical dimension, was also more efficient than using a 1D approach with only a spatial reaction coordinate, as shown in Fig. 3. The main drawback with an alchemical free energy reaction coordinate dimension is that the simulation throughput is lower compared with FR pulling simulations as well as 1D AWH simulations using only a spatial coordinate. However, the possibility to more accurately sample the free energy landscape makes it attractive for problems that are difficult to address with conventional methods, such as permeability coefficient predictions in gel-phase systems, like the skin's barrier structure.

Another advantage is that it is easier to prepare permeability calculation simulations using an alchemical dimension than with conventional procedures, as the permeant molecule can be inserted anywhere in the system with all interactions turned off. The AWH simulations will then turn on the interactions where favorable. On the contrary, with standard FR pulling, as well as with 1D AWH pulling or umbrella simulations, the molecule must be grown into the system and/or pulled to suitable starting positions, followed by equilibration of the simulation system, before the production simulations are started. To determine the free energy landscape without an alchemical dimension in the calculation, it would also be necessary to know the free energy of inserting the molecular for at least one position along the barrier structure; otherwise the PMF cannot be calibrated compared with a solution or formulation.

The permeability coefficient calculations presented herein only account for the passage of a chemical compound across the skin's barrier structure. Lateral diffusion within the skin's barrier structure is not calculated, and permeation through the stratum corneum cell interiors, as well as through viable cell layers of epidermis, are ignored. Likewise, potential contributions from the corneocyte cell lipid envelopes are not included. While these are admittedly approximations, the contributions from these factors are expected to be negligible in comparison with the permeation resistance across the skin's barrier structure for all but very lipophilic compounds (74). It is primarily the permeability across the skin's barrier structure that can be tuned using chemical permeation enhancers, and thereby exploited in transdermal drug delivery design.

As can be inferred from Wang and Klauda (35) and from Scheuplein and Blank (74), as well as from Eq. 20 in Marrink and Berendsen (67), and discussed in the introduction, the permeability coefficient does not correspond to an average permeation speed through the skin, but will become lower the thicker the skin sample. Therefore, the permeability coefficient through the skin's barrier structure alone would be higher than through full-thickness skin samples used in ex vivo/in vitro permeability measurements. We would therefore expect calculated permeability coefficients, referring exclusively to the skin's barrier structure, to be slightly higher than those measured ex vivo/in vitro through full-thickness skin, assuming that the skin samples are intact. Our calculated log K_P is 0.38 cm h^{-1} higher than experimental results on average, and 0.44 cm h^{-1} higher if excluding urea and hydromorphone—the two largest outliers.

Measuring skin permeability using diffusion cells, such as Franz cells, requires long experiment times: how long depends on the permeant's lag time (75). It is common to run experiments for 24–72 h (75). In extreme cases, with low permeability and long lag times, this might even be insufficient. However, long-time exposure to water leads to skin degradation and increased skin permeability (75,76). These factors can make it difficult to compare theoretical results with those obtained ex vivo/in vitro, since the ex vivo/in vitro measurements may sometimes be too short for the permeation lag time and too long for keeping the skin sample intact.

On the other hand, the main disadvantage with MD simulations is that they take a lot longer to run compared with in vitro/ex vivo experiments. This is even more striking when it comes to studying the gel-like lipid barrier structure in stratum corneum, which is the problem we have mitigated using more efficient sampling. Even if MD simulations do not need any training, in contrast to QSAR models, they still depend on the quality of the force field that is used. Given the resource requirements, MD simulations should be considered a complement to laboratory diffusion cell experiments, as well as QSPR calculations or other mathematical models. There is no reason to exclusively depend on one method.

The largest outliers in this study (see Table S1) were urea (log K_P diff. −2.1 cm h^{-1}) and hydromorphone (log K_P

diff. = 1.8 cm h^{-1}), followed by salicylic acid and ethanol. The correlation plot of the results, excluding urea and hydromorphone, is shown in Fig. S8. The permeation enhancing effects of urea (77) were not taken into account in these simulations, which could be a reason for the discrepancy. QSPR simulations also underestimate the permeability coefficient of urea, but only by 0.6 log units on average (21,22,25,78), so another explanation is needed. It has been proposed that small hydrophilic permeants pass through "polar pathways," shunts, or through lipid defects (79). Lateral diffusion in the lipid barrier structure may be important to find these more permeable regions (34), even if their exact nature is not yet fully explained. For hydromorphone, there is a remarkable similarity between the average of three different predicted log K_P values obtained using mathematical QSPR models of −3.2 cm h^{-1} (21,22,25,78) and our predicted log K_P at 32°C of −2.9 cm h^{-1}, contrasting with the experimentally obtained value of −4.8 cm h^{-1} at 37°C (80), with no corrections for pH/pK_a. This discrepancy between predicted and experimental data may call for re-investigation of the experimentally measured permeability coefficient of hydromorphone, preferably from multiple laboratories, although it could of course also indicate permeation properties not picked up by QSPR models either. Looking at Fig. S7, the calculated and experimental log K_P square difference does not seem to correlate specifically with molecular mass or log $P_{o/w}$.

CONCLUSION

We have shown that the model of the skin's barrier structure that was proposed a few years ago (6) can be used to accurately predict permeability coefficients of a large spectrum of molecules, with clearly better computational efficiency than previous methods.

To our knowledge, this is the first report describing the use of a 2D reaction coordinate with one spatial dimension and one alchemical free energy dimension. It is also the first study to employ the AWH method to sample such a free energy landscape. The obtained PMFs are detailed and some of their features only hinted at when using FR pulling simulations with very slow pulling speeds. When taking the multiple bilayers of the skin's barrier structure into account, our previously published calculations underestimated the skin permeability coefficients (7), largely explained by the inability of the FR pulling method to properly sample the free energy landscape.

Experimental permeability coefficient measurements are associated with variations between laboratories as well as with inter- and intraindividual differences. This makes it treacherous to strictly compare calculated permeability coefficients with experimental data. This needs, particularly, to be taken into account when creating QSPR models (4). A suggested approach for building a reliable QSPR model would therefore be that either all training data should come from one laboratory using the same experimental setup for all compounds or, preferably, there should be measurements from several laboratories, using the same methods for each compound. When predicting permeability coefficients using MD simulation there is no need for training the method with experimental data, but reliable experimental measurements are required for verifying the techniques and simulation results.

The correlation between calculated and experimental permeability coefficients presented here proves the usefulness of MD simulation for transdermal drug delivery design, although the method's precision may presently not always allow for differentiation of very similar molecules.

We do not argue that MD simulations should replace ex vivo/in vitro measurements, but the two methods can complement each other. Perhaps most important for transdermal drug delivery design is that MD simulation can explain experimental ex vivo/in vitro data by predicting where in the skin's barrier structure the main permeation barriers are located for each specific permeant. This knowledge, along with the PMFs (in effect partition profiles) of chemical permeation enhancers, can help selecting suitable drug delivery formulation excipient combinations tailored for each specific API.

SUPPORTING MATERIAL

Supporting material can be found online at https://doi.org/10.1016/j.bpj.2022.09.009.

DATA AVAILABILITY

The input data and parameters are available for download from https://doi.org/10.5281/zenodo.5883410.

AUTHOR CONTRIBUTIONS

M.L., L.N., C.W., and E.L. designed the research. M.L., J.L., B.H., and C.W. developed the simulation protocols. M.L. carried out all simulations and analyzed the data with assistance from C.W. All authors wrote the article, with M.L. being the primary author.

ACKNOWLEDGMENTS

The computations were enabled by resources provided by the Swedish National Infrastructure for Computing (SNIC 2020/5-220 and 2021/5-217) at PDC Center for High Performance Computing at KTH and NSC at Linköping University, as well as internal resources from ERCO Pharma AB and E.L.'s research group. The authors also gratefully acknowledge the HPC RIVR consortium (www.hpc-rivr.si) and EuroHPC JU (eurohpc-ju.europa.eu) for funding this research by providing computing resources of the HPC system Vega at the Institute of Information Science (www.izum.si).

DECLARATION OF INTERESTS

ERCO Pharma AB has submitted a patent application covering using this lipid barrier model for skin permeability calculations (application no. PCT/EP2017/076,237 and title "skin permeability prediction").

L.N., M.L., and C.W. have stock options in ERCO Pharma AB. M.L. and C.W. are employed by ERCO Pharma AB.

REFERENCES

1. Todo, H. 2017. Transdermal permeation of drugs in various animal species. *Pharmaceutics.* 9:33. http://www.mdpi.com/1999-4923/9/3/33.
2. Buist, H., P. Craig, A. Chiusolo; …, European Food Safety Authority EFSA. 2017. Guidance on dermal absorption. *EFSA J.* 15:4873. https://efsa.onlinelibrary.wiley.com/doi/abs/10.2903/j.efsa.2017.4873.
3. Jung, E. C., and H. I. Maibach. 2015. Animal models for percutaneous absorption: animal models for percutaneous absorption. *J. Appl. Toxicol.* 35:1–10.
4. Degim, I., W. Pugh, and J. Hadgraft. 1998. Skin permeability data: anomalous results. *Int. J. Pharm. X.* 170:129–133. http://www.sciencedirect.com/science/article/pii/S0378517398001136.
5. Pecoraro, B., M. Tutone, …, M. Traynor. 2019. Predicting skin permeability by means of computational approaches: reliability and caveats in pharmaceutical studies. *J. Chem. Inf. Model.* 59:1759–1771. http://pubs.acs.org/doi/10.1021/acs.jcim.8b00934.
6. Lundborg, M., A. Narangifard, …, L. Norlén. 2018. Human skin barrier structure and function analyzed by cryo-EM and molecular dynamics simulation. *J. Struct. Biol.* 203:149–161. http://www.sciencedirect.com/science/article/pii/S1047847718301138.
7. Lundborg, M., C. L. Wennberg, …, L. Norlén. 2018. Predicting drug permeability through skin using molecular dynamics simulation. *J. Contr. Release.* 283:269–279. http://www.sciencedirect.com/science/article/pii/S016836591830292X.
8. Lundborg, M., J. Lidmar, and B. Hess. 2021. The accelerated weight histogram method for alchemical free energy calculations. *J. Chem. Phys.* 154:204103. https://aip.scitation.org/doi/10.1063/5.0044352.
9. Rizzi, A., T. Jensen, …, J. D. Chodera. 2020. The SAMPL6 SAMPLing challenge: assessing the reliability and efficiency of binding free energy calculations. *J. Comput. Aided Mol. Des.* 34:601–633. http://link.springer.com/10.1007/s10822-020-00290-5.
10. Breathnach, A. S., T. Goodman, …, M. Gross. 1973. Freeze-fracture replication of cells of stratum corneum of human epidermis. *J. Anat.* 114:65–81. https://www.ncbi.nlm.nih.gov/pmc/articles/PMC1271426/.
11. Elias, P. M., and D. S. Friend. 1975. The permeability barrier in mammalian epidermis. *J. Cell Biol.* 65:180–191. http://jcb.rupress.org/content/65/1/180.
12. Swartzendruber, D. C., P. W. Wertz, …, D. T. Downing. 1989. Molecular models of the intercellular lipid lamellae in mammalian stratum corneum. *J. Invest. Dermatol.* 92:251–257. http://www.sciencedirect.com/science/article/pii/S0022202X89901619.
13. Bouwstra, J., G. Pilgram, …, M. Ponec. 2001. New aspects of the skin barrier organization. *Skin Pharmacol. Appl. Skin Physiol.* 14:52–62. http://www.karger.com/?doi=10.1159/000056391.
14. Hill, J. R., and P. W. Wertz. 2003. Molecular models of the intercellular lipid lamellae from epidermal stratum corneum. *Biochim. Biophys. Acta.* 1616:121–126. http://www.sciencedirect.com/science/article/pii/S0005273603002384.
15. McIntosh, T. J. 2003. Organization of skin stratum corneum extracellular lamellae: diffraction evidence for asymmetric distribution of cholesterol. *Biophys. J.* 85:1675–1681. http://www.sciencedirect.com/science/article/pii/S0006349503745974.
16. Schröter, A., D. Kessner, R. H. H. Neubert…, 2009. Basic nanostructure of stratum corneum lipid matrices based on ceramides [EOS] and [AP]: a neutron diffraction study. *Biophys. J.* 97:1104–1114. http://www.sciencedirect.com/science/article/pii/S0006349509010492.
17. Iwai, I., H. Han, …, L. Norlén. 2012. The human skin barrier is organized as stacked bilayers of fully extended ceramides with cholesterol molecules associated with the ceramide sphingoid moiety. *J. Invest. Dermatol.* 132:2215–2225. http://www.nature.com/jid/journal/v132/n9/abs/jid201243a.html. https://creativecommons.org/licenses/by-nc-nd/3.0/legalcode.
18. Rullgård, H., L.-G. Öfverstedt, …, O. Öktem. 2011. Simulation of transmission electron microscope images of biological specimens. *J. Microsc.* 243:234–256.
19. Norlén, L., M. Lundborg, …, B. Daneholt. 2022. The skin's barrier: a cryo-EM based overview of its architecture and stepwise formation. *J. Invest. Dermatol.* 142:285–292. https://linkinghub.elsevier.com/retrieve/pii/S0022202X21016870.
20. Mojumdar, E. H., G. S. Gooris, …, J. A. Bouwstra. 2016. Stratum corneum lipid matrix: location of acyl ceramide and cholesterol in the unit cell of the long periodicity phase. *Biochim. Biophys. Acta.* 1858:1926–1934. http://www.sciencedirect.com/science/article/pii/S0005273616301493.
21. Potts, R. O., and R. H. Guy. 1992. Predicting skin permeability. *Pharm. Res.* 9:663–669. http://link.springer.com/article/10.1023/A%3A1015810312465.
22. Mitragotri, S. 2002. A theoretical analysis of permeation of small hydrophobic solutes across the stratum corneum based on Scaled Particle Theory. *J. Pharmacol. Sci.* 91:744–752. http://linkinghub.elsevier.com/retrieve/pii/S0022354916309327.
23. Abraham, M. H., and F. Martins. 2004. Human skin permeation and partition: general linear free-energy relationship analyses. *J. Pharmacol. Sci.* 93:1508–1523.
24. Tsakovska, I., I. Pajeva, …, M. T. D. Cronin. 2017. Quantitative structure-skin permeability relationships. *Toxicology.* 387:27–42. https://linkinghub.elsevier.com/retrieve/pii/S0300483X17301750.
25. Lian, G., L. Chen, and L. Han. 2008. An evaluation of mathematical models for predicting skin permeability. *J. Pharmacol. Sci.* 97:584–598. http://linkinghub.elsevier.com/retrieve/pii/S0022354916324388.
26. Pugh, W. J., R. Wong, …, G. P. Moss. 2005. Discriminant analysis as a tool to identify compounds with potential as transdermal enhancers. *J. Pharm. Pharmacol.* 57:1389–1396. https://academic.oup.com/jpp/article/57/11/1389/6147618.
27. Albery, W. J., and J. Hadgraft. 1979. Percutaneous absorption: in vivo experiments. *J. Pharm. Pharmacol.* 31:140–147.
28. Lee, S. H., S. K. Jeong, and S. K. Ahn. 2006. An update of the defensive barrier function of skin. *Yonsei Med. J.* 47:293–306. https://synapse.koreamed.org/DOIx.php?id=10.3349/ymj.2006.47.3.293.
29. Wang, T.-F., G. B. Kasting, and J. M. Nitsche. 2006. A multiphase microscopic diffusion model for stratum corneum permeability. I. Formulation, solution, and illustrative results for representative compounds. *J. Pharmacol. Sci.* 95:620–648. http://linkinghub.elsevier.com/retrieve/pii/S0022354916319633.
30. Naegel, A., S. Hansen, …, M. Heisig. 2008. In-silico model of skin penetration based on experimentally determined input parameters. Part II: mathematical modelling of in-vitro diffusion experiments. Identification of critical input parameters. *Eur. J. Pharm. Biopharm.* 68:368–379. https://linkinghub.elsevier.com/retrieve/pii/S0939641107002032.
31. Selzer, D., D. Neumann, and U. F. Schaefer. 2015. Mathematical models for dermal drug absorption. *Expert Opin. Drug Metabol. Toxicol.* 11:1567–1583. http://www.tandfonline.com/doi/full/10.1517/17425255.2015.1063615.
32. Das, C., P. D. Olmsted, and M. G. Noro. 2009. Water permeation through stratum corneum lipid bilayers from atomistic simulations. *Soft Matter.* 5:4549–4555. http://pubs.rsc.org.ezp.sub.su.se/en/content/articlelanding/2009/sm/b911257j.
33. Gupta, R., D. B. Sridhar, and B. Rai. 2016. Molecular dynamics simulation study of permeation of molecules through skin lipid bilayer. *J. Phys. Chem. B.* 120:8987–8996.
34. Del Regno, A., and R. Notman. 2018. Permeation pathways through lateral domains in model membranes of skin lipids. *Phys. Chem. Chem. Phys.* 20:2162–2174.
35. Wang, E., and J. B. Klauda. 2019. Molecular structure of the long periodicity phase in the stratum corneum. *J. Am. Chem. Soc.* 141:16930–16943. https://pubs.acs.org/doi/10.1021/jacs.9b08995.
36. MacDermaid, C. M., K. W. Hall, …, G. Fiorin. 2020. Coexistence of lipid phases stabilizes interstitial water in the outer layer of mammalian

skin. *Biophys. J.* 118:1588–1601. https://linkinghub.elsevier.com/retrieve/pii/S0006349520301211.

37. Crooks, G. E. 1999. Entropy production fluctuation theorem and the nonequilibrium work relation for free energy differences. *Phys. Rev. E.* 60:2721–2726. http://link.aps.org/doi/10.1103/PhysRevE.60.2721.
38. Crooks, G. E. 2000. Path-ensemble averages in systems driven far from equilibrium. *Phys. Rev. E.* 61:2361–2366. http://link.aps.org/doi/10.1103/PhysRevE.61.2361.
39. Kosztin, I., B. Barz, and L. Janosi. 2006. Calculating potentials of mean force and diffusion coefficients from nonequilibrium processes without Jablonski's equality. *J. Chem. Phys.* 124:064106.
40. Chen, L. Y. 2008. Nonequilibrium fluctuation-dissipation theorem of Brownian dynamics. *J. Chem. Phys.* 129:144113.
41. Wang, E., and J. B. Klauda. 2019. Structure and permeability of ceramide bilayers and multilayers. *J. Phys. Chem. B.* 123:2525–2535.
42. Lidmar, J. 2012. Improving the efficiency of extended ensemble simulations: the accelerated weight histogram method. *Phys. Rev. E - Stat. Nonlin. Soft Matter Phys.* 85:056708.
43. Lindahl, V., J. Lidmar, and B. Hess. 2014. Accelerated weight histogram method for exploring free energy landscapes. *J. Chem. Phys.* 141:044110.
44. Mey, A. S. J. S., B. K. Allen, …, H. Xu. 2020. Best practices for alchemical free energy calculations [article v1.0]. *LiveCoMS.* 2:18378. https://www.livecomsjournal.org/article/18378-best-practices-for-alchemical-free-energy-calculations-article-v1-0.
45. Ya-Xian, Z., T. Suetake, and H. Tagami. 1999. Number of cell layers of the stratum corneum in normal skin - relationship to the anatomical location on the body, age, sex and physical parameters. *Arch. Dermatol. Res.* 291:555–559. http://link.springer.com/10.1007/s004030050453.
46. Al-Amoudi, A., J. Dubochet, and L. Norlén. 2005. Nanostructure of the epidermal extracellular space as observed by cryo-electron microscopy of vitreous sections of human skin. *J. Invest. Dermatol.* 124:764–777. http://www.jidonline.org/article/S0022202X15322260/abstract.
47. Norlén, L. 2022. Personal Communication: Number of Lipid Bilayers Between Corneocytes in CEMOVIS Images from Human Stratum Corneum.
48. Abraham, M. J., T. Murtola, E. Lindahl…, 2015. GROMACS: high performance molecular simulations through multi-level parallelism from laptops to supercomputers. *SoftwareX.* 1–2:19–25. http://www.sciencedirect.com/science/article/pii/S2352711015000059.
49. Lindahl, E., M. J. Abraham, …, D. van der Spoel. 2021. GROMACS 2021 Manual.https://doi.org/10.5281/zenodo.4457591.
50. Páll, S., A. Zhmurov, …, E. Lindahl. 2020. Heterogeneous parallelization and acceleration of molecular dynamics simulations in GROMACS. *J. Chem. Phys.* 153:134110.
51. GROMACS. 2022. GROMACS Gitlab: 2022-Awhsymm-Awhcorrblocks. https://gitlab.com/gromacs/gromacs/-/tree/2022-awhsymm-awhcorrblocks.
52. Essmann, U., L. Perera, …, L. G. Pedersen. 1995. A smooth particle mesh Ewald method. *J. Chem. Phys.* 103:8577–8593. http://jcp.aip.org/resource/1/jcpsa6/v103/i19/p8577_s1?isAuthorized=no.
53. Hess, B., H. Bekker, …, J. G. E. M. Fraaije. 1997. LINCS: a linear constraint solver for molecular simulations. *J. Comput. Chem.* 18:1463–1472.
54. Miyamoto, S., and P. A. Kollman. 1992. Settle: an analytical version of the SHAKE and RATTLE algorithm for rigid water models. *J. Comput. Chem.* 13:952–962.
55. Jorgensen, W. L., J. Chandrasekhar, …, M. L. Klein. 1983. Comparison of simple potential functions for simulating liquid water. *J. Chem. Phys.* 79:926–935. http://link.aip.org/link/JCPSA6/v79/i2/p926/s1&Agg=doi.
56. Klauda, J. B., R. M. Venable, …, R. W. Pastor. 2010. Update of the CHARMM all-atom additive force field for lipids: validation on six lipid types. *J. Phys. Chem. B.* 114:7830–7843.
57. Venable, R. M., A. J. Sodt, …, J. B. Klauda. 2014. CHARMM all-atom additive force field for sphingomyelin: elucidation of hydrogen bonding and of positive curvature. *Biophys. J.* 107:134–145. http://www.sciencedirect.com/science/article/pii/S0006349514005724.
58. Dahlén, B., and I. Pascher. 1979. Molecular arrangements in sphingolipids. Thermotropic phase behaviour of tetracosanoylphytosphingosine. *Chem. Phys. Lipids.* 24:119–133. http://www.sciencedirect.com/science/article/pii/0009308479900823.
59. Bernetti, M., and G. Bussi. 2020. Pressure control using stochastic cell rescaling. *J. Chem. Phys.* 153:114107.
60. Beutler, T. C., A. E. Mark, …, W. F. van Gunsteren. 1994. Avoiding singularities and numerical instabilities in free energy calculations based on molecular simulations. *Chem. Phys. Lett.* 222:529–539. https://linkinghub.elsevier.com/retrieve/pii/0009261494003971.
61. Lundborg, M., and E. Lindahl. 2015. Automatic GROMACS topology generation and comparisons of force fields for solvation free energy calculations. *J. Phys. Chem. B.* 119:810–823.
62. O'Boyle, N. M., M. Banck, …, G. R. Hutchison. 2011. Open Babel: an open chemical toolbox. *J. Cheminf.* 3:33. http://www.jcheminf.com/content/3/1/33/abstract.
63. Yesselman, J. D., D. J. Price, …, C. L. Brooks, 3rd. 2012. MATCH: an atom-typing toolset for molecular mechanics force fields. *J. Comput. Chem.* 33:189–202.
64. Vanommeslaeghe, K., E. Hatcher, …, A. D. Mackerell. 2010. CHARMM general force field: a force field for drug-like molecules compatible with the CHARMM all-atom additive biological force fields. *J. Comput. Chem.* 31:671–690.
65. Stone, J. 1998. An efficient library for parallel ray tracing and animation. Master's Thesis. University of Missouri-Rolla.
66. Humphrey, W., A. Dalke, and K. Schulten. 1996. VMD: visual molecular dynamics. *J. Mol. Graph.* 14:33–38. http://www.sciencedirect.com/science/article/pii/0263785596000185.
67. Marrink, S.-J., and H. J. C. Berendsen. 1994. Simulation of water transport through a lipid membrane. *J. Phys. Chem. A.* 98:4155–4168. https://doi.org/10.1021/j100066a040.
68. Venable, R. M., A. Krämer, and R. W. Pastor. 2019. Molecular dynamics simulations of membrane permeability. *Chem. Rev.* 119:5954–5997.
69. Lindahl, V., J. Lidmar, and B. Hess. 2018. Riemann metric approach to optimal sampling of multidimensional free-energy landscapes. *Phys. Rev. E.* 98:023312.
70. Mitragotri, S., D. A. Edwards, …, R. Langer. 1995. A mechanistic study of ultrasonically-enhanced transdermal drug delivery. *J. Pharmacol. Sci.* 84:697–706.
71. Scheuplein, R. J., I. H. Blank, …, D. J. Macfarlane. 1969. Percutaneous absorption of steroids. *J. Invest. Dermatol.* 52:63–70. http://www.sciencedirect.com/science/article/pii/S0022202X15476195.
72. An-eX Analytical Services Ltd. 2017. Assessment of the permeation of testosterone through human skin in vitro from saturated aqueous solution. *In* Technical Report E05/02/17.
73. Siddiqui, O., M. S. Roberts, and A. E. Polack. 1989. Percutaneous absorption of steroids: relative contributions of epidermal penetration and dermal clearance. *J. Pharmacokinet. Biopharm.* 17:405–424.
74. Scheuplein, R. J., and I. H. Blank. 1973. Mechanism of percutaneous absorption. Iv. Penetration of nonelectrolytes (alcohols) from aqueous solutions and from pure liquids. *J. Invest. Dermatol.* 60:286–296. http://www.nature.com.ezp.sub.su.se/jid/journal/v60/n5/abs/5617718a.html.
75. Finnin, B., K. A. Walters, and T. J. Franz. 2011. In vitro skin permeation methodology. *In* Topical and Transdermal Drug Delivery. John Wiley & Sons, Ltd, pp. 85–108.
76. van Hal, D. A., E. Jeremiasse, …, J. A. Bouwstra. 1996. Structure of fully hydrated human stratum corneum: a freeze-fracture electron microscopy study. *J. Invest. Dermatol.* 106:89–95. http://www.sciencedirect.com/science/article/pii/S0022202X1542408X.

77. Trommer, H., and R. H. H. Neubert. 2006. Overcoming the stratum corneum: the modulation of skin penetration. *Skin Pharmacol. Physiol.* 19:106–121. https://www.karger.com/Article/FullText/91978.

78. Abraham, M. H., H. S. Chadha, …, J. A. Gratton. 1999. Hydrogen bonding part 46: a review of the correlation and prediction of transport properties by an lfer method: physicochemical properties, brain penetration and skin permeability. *Pestic. Sci.* 55:78–88.

79. Mitragotri, S. 2003. Modeling skin permeability to hydrophilic and hydrophobic solutes based on four permeation pathways. *J. Contr. Release.* 86:69–92.

80. Roy, S. D., and G. L. Flynn. 1989. Transdermal delivery of narcotic analgesics: comparative permeabilities of narcotic analgesics through human cadaver skin. *Pharm. Res.* 6:825–832.

Biophysical Journal, Volume *121*

Supplemental information

Skin permeability prediction with MD simulation sampling spatial and alchemical reaction coordinates

Magnus Lundborg, Christian Wennberg, Jack Lidmar, Berk Hess, Erik Lindahl, and Lars Norlén

Supplement

Supplementary information for:
Skin Permeability Prediction with MD Simulation Sampling Spatial and Alchemical Reaction Coordinates

Magnus Lundborg[1,*], Christian Wennberg[1], Jack Lidmar[2], Berk Hess[3], Erik Lindahl[4,5], and Lars Norlén[6,7,*]

[1]ERCO Pharma AB, Science for Life Laboratory, Solna, Sweden
[2]Department of Physics, KTH Royal Institute of Technology, Stockholm, Sweden
[3]Department of Applied Physics, Science for Life Laboratory, KTH Royal Institute of Technology, Solna, Sweden
[4]Department of Biophysics and Biochemistry, Science for Life Laboratory, Stockholm University, Solna, Sweden
[5]Department of Physics, Swedish e-Science Research Center, KTH Royal Institute of Technology, Stockholm, Sweden
[6]Department of Cell and Molecular Biology (CMB), Karolinska Institutet, Stockholm, Sweden
[7]Dermatology Clinic, Karolinska University Hospital, Stockholm, Sweden
[*]Correspondence: magnus.lundborg@ercopharma.com and lars.norlen@ki.se

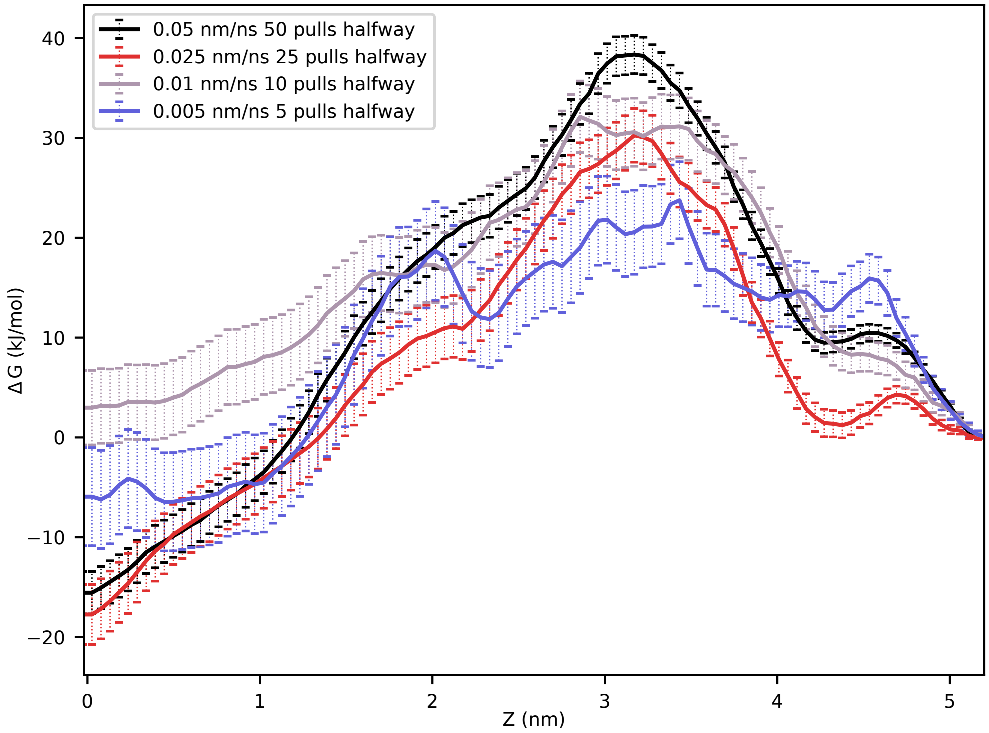

Figure S1: PMFs of testosterone, from forward-reverse pulling simulations, with different pulling speeds. The results are from 10 µs of sampling and the PMFs are calibrated to 0 at the ceramide sphingoid chain interface (5.2 nm), to make comparisons easier. The general trend is that slower pulling speeds give lower free energy barriers and more detailed PMFs. However, fewer pulls also means a larger variability. Two permeants were pulled halfway through the system in order to shorten the simulations.

S1 METHODS

When running AWH simulations it is possible to run multiple simulations in parallel, referred to as AWH walkers. The simulations can share their sampling, and thereby use the same bias, by communicating at regular intervals. This can improve the total simulation throughput compared to running a single simulation for a longer time. For the simulations of the lipid barrier structure 24 MD simulations with communicating AWH walkers were run, whereas 16 communicating AWH walkers were used for the hydration free energy calculations. The AWH histograms were equilibrated as part of the initial stage, using the criterion that at least 80 % of the target region was sampled within 20 % of the target distribution.

During the course of this project it was discovered that GROMACS 2021 running AWH with an alchemical free energy dimension did not properly take PME contributions into account along the alchemical dimension when calculating PME on a GPU accelerator. After this bug was fixed all hydration free energy calculations were rerun as well as at least two independent sets, with 24 communicating AWH walkers, each running 450 ns, calculating the permeability through the barrier. It was found that a systematic shift in the PMF, relative to the vacuum state, in the range of 0 kJ mol^{-1} to 13 kJ mol^{-1} depending on the permeant, was the only significant difference. The PMF results from the GROMACS version with the bug were shifted to align with the new correct output.

S2 RESULTS

S2.1 Comparisons to other MD simulation methods

In the data presented in this section we pulled testosterone using a constraint, i.e., an infinitely stiff spring, to avoid having to select a suitable spring constant for each permeant (1). Compared to the 2018 study we also equilibrated the system longer after inserting the two copies of the permeant into the system, (20 ns instead of 1 ns). As can be seen in Fig. S1 the results, for testosterone, were very dependent on the pulling speeds. As these results are not the focus of this study — they are just included for comparison — we will refer to the original paper(2) for a more thorough description of the methods.

We also evaluated umbrella sampling simulations for calculating the PMFs. We inserted four copies of testosterone, evenly

distributed along the reaction coordinate, and grew them in, by turning on their interactions with their environment, over a period of 1 ns. This was repeated in 80 steps along the reaction coordinate, giving 320 umbrella sampling positions at random lateral positions. During the umbrella simulations the permeants were held in place using a spring constant of 5000 kJ mol^{-1} nm^{-2}. The 80 simulations were run for 150 ns (for a total of 12 000 ns). To estimate the equilibration times (how much to discard in the beginning of the simulations) a time window of 100 ns, with a shifting starting time, was analyzed. As can be seen in Fig. S2, even if the difference between the shortest and longest equilibration times was not statistically significant, there was an obvious systematic drift in the PMF with increasing equilibration times, most clearly seen in Fig. S2b. This indicated that discarding 50 ns (out of 150 ns) might not be enough. While, in the case shown here, the effect is not very large for the symmetrized PMF (Fig. S2a) the long times to reach convergence was not very attractive. Comparing Fig. S2a) to the previously published results from FR pulling simulations(2), shown by the red curve in the Testosterone sub figure in Fig. S5, indicates that the height of the free energy profile from these umbrella simulations roughly matched the ones from FR pulling at a pulling speed of 0.2 nm ns^{-1}. However, they required approximately the same total simulation time as the pulling simulations at 0.005 nm ns^{-1}. We expect that the umbrella sampling simulations can be further optimized, and do not claim that umbrella sampling is better or worse than FR pulling. Both methods face similar challenges with the long correlation times of the skin's barrier system existing in a gel-like state.

S2.2 Comparison of testosterone PMFs from 2D AWH to FR Pulling

Using the slowest pulling speed shown in Fig. S1 we compared the data from FR pulling simulations at 10 and 30 μs to the two-dimensional AWH setup, that was used for this project, with 1, 3 and 5 independent sets of simulations (11 , 32 and 55 μs). The results are presented in Fig. S3 and show that the differences were small between the AWH simulations with 3 and 5 sets and that the results from 30 μs FR pulling were further away from the final AWH results than the results after 1 set (11 μs) of AWH simulations. It can also be noted that the results from FR pulling simulations seem to approach those from AWH, but since the output from 30 μs of FR pulling is still far from the AWH results it can be concluded that many more FR pulling simulations, in order to achieve a longer total simulation time, would be needed to come close to the AWH results. Looking also at the testosterone diffusion coefficient profile in Fig. S6 it can be concluded that the largest differences between FR pulling and AWH are observed in the region with the slowest diffusion, i.e., Z ~2.5 nm to ~4.5 nm.

S2.3 Permeability Coefficient Calculations

The permeability coefficients were calculated using Eq. 1 (in the main paper) using the PMF and diffusion coefficient profiles presented in Figs. S5 and S6. The results are presented in Table S1 and in Fig. 4 (in the main paper).

In this study we have been focusing on the permeability coefficients of neutral (non-ionized) forms of the permeants. There were eight molecules that are ionizable under the *ex vivo/in vitro* conditions: codeine, diclofenac, hydromorphone, ibuprofen, lidocaine, naproxen, nicotine and salicylic acid. For most of them there are published permeability coefficients available, whereas for codeine and hydromorphone the corrections by Abraham and Martins (3) were used. In order to examine whether the neutral forms of ionizable permeants were in agreement with experimental results we divided the molecules into two groups, separating those that are not ionizable under the *ex vivo/in vitro* conditions from those that are ionizable. The correlation plots from these analyses are presented in Fig. S9 and show that there is no clear difference between the two groups, but the permeability coefficients of the set of non-ionizable compounds are better predicted by MD simulations.

We also studied whether there was any clear correlation between permeant molecular mass or log $P_{o/w}$ and large differences between experimental and calculated permeability coefficients. The results are presented in Fig. S7.

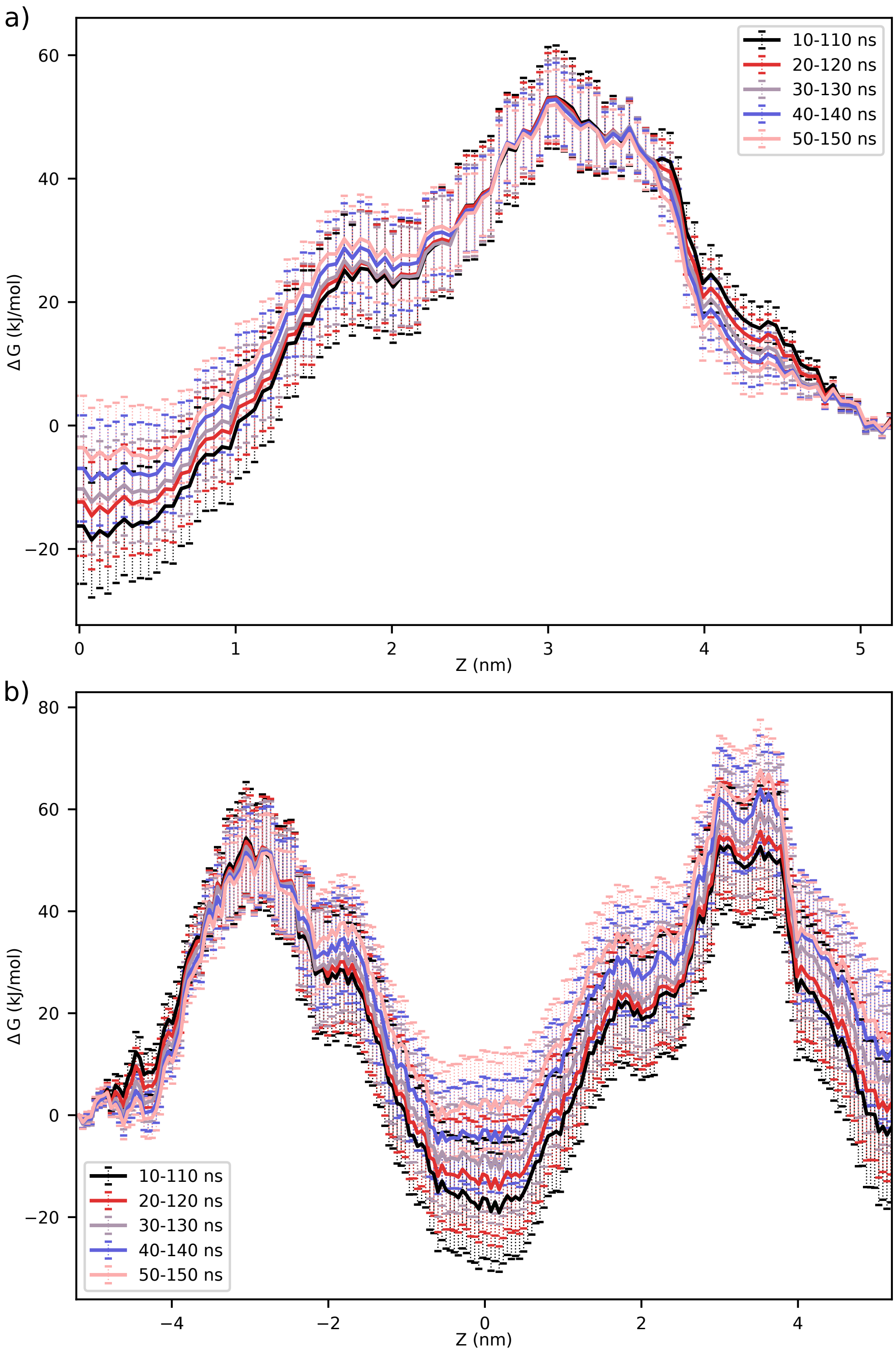

Figure S2: PMFs of testosterone from umbrella sampling simulations with increasing equilibration time. In a) the data is enforced to be periodic, with connected end points, and symmetrized. The PMFs are calibrated to 0 at the ceramide sphingoid chain interface (5.2 nm), to make comparisons easier. In b) the data is not symmetrized without connected end points (the PMF is set to 0 only at −5.2 nm).

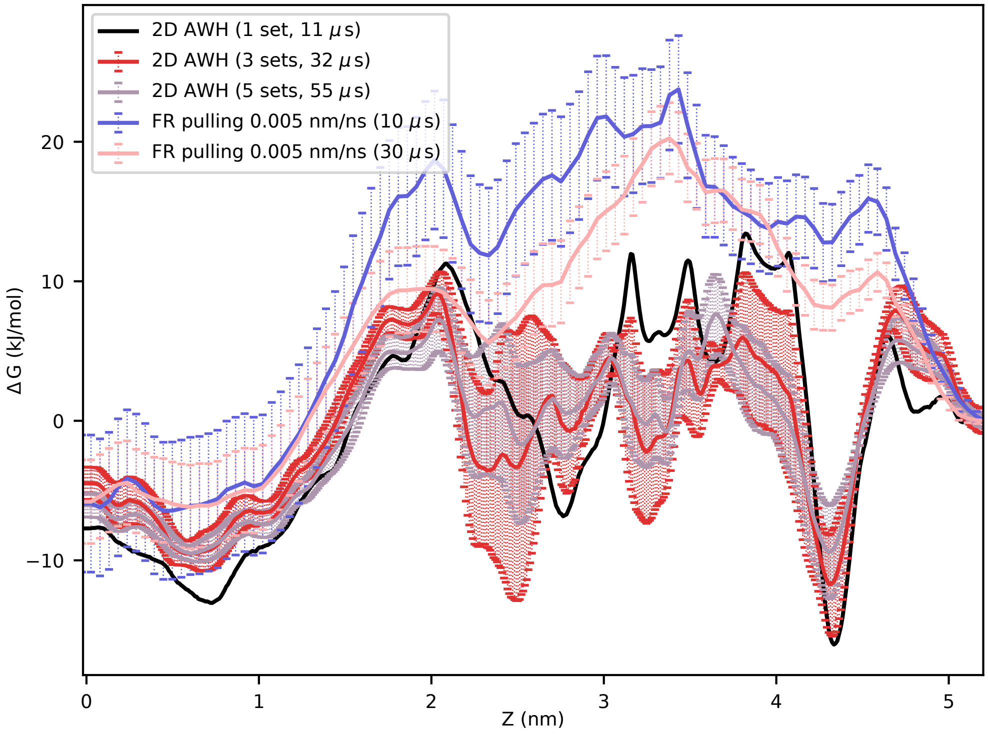

Figure S3: PMFs of testosterone comparing the 2D spatial/alchemical AWH (in the fully interacting alchemical λ state) results to slow forward-reverse pulling experiments. In both cases the PMFs are symmetrized and only half the PMFs are presented and the PMFs are calibrated to 0 at the ceramide sphingoid chain interface (5.2 nm), to make comparisons easier. AWH analyses do not give a reliable error estimate from one set of simulations, therefore there is no error presented for the 1 set, 11 µs AWH plot (in black). In the pulling experiments two permeants were pulled halfways through the system in order to shorten the simulations. FR pulling simulations of 30 µs required approximately the same computation time as 11 µs of 2D AWH.

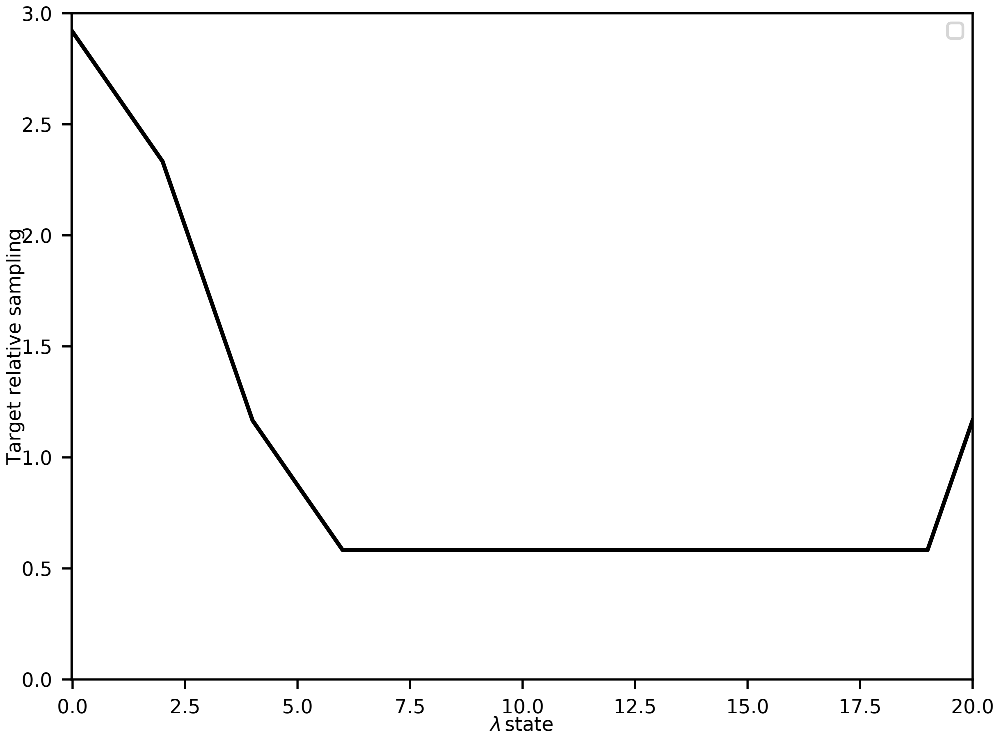

Figure S4: AWH target distribution along the alchemical free energy axis. λ state 0 was fully interacting and at λ state 20 there were no interactions between the molecule and its surroundings. Along the pull axis the target distribution was uniform.

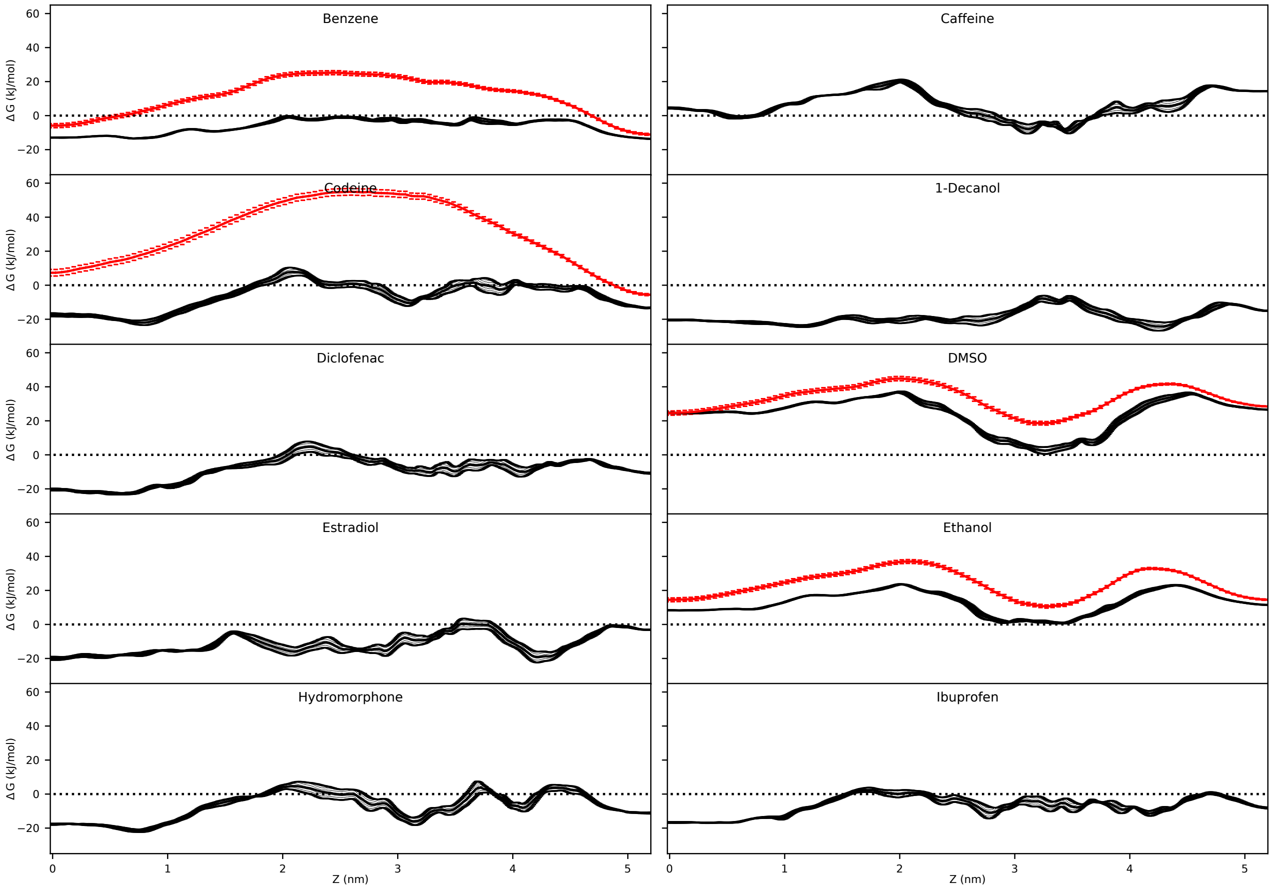

Figure S5: PMFs of 20 permeants. The PMFs are calibrated relative to the hydration free energies of the compounds. The profiles in black show the results from this study, whereas the profiles in red are from our previous FR pulling simulations (pulling at 0.2 nm ns^{-1}) (2). The curves in red were only calibrated based on the free energy difference, to the water solvent, at the ceramide sphingoid chain interface region (at ~5.2 nm). When calculating the permeability coefficients, the PMFs (in black) were shifted (up) so that their minima were ≥0 kJ mol^{-1}. I.e., the zero point was set to the lowest of the hydration free energy and the lowest point in the PMF. *Continued on next page.*

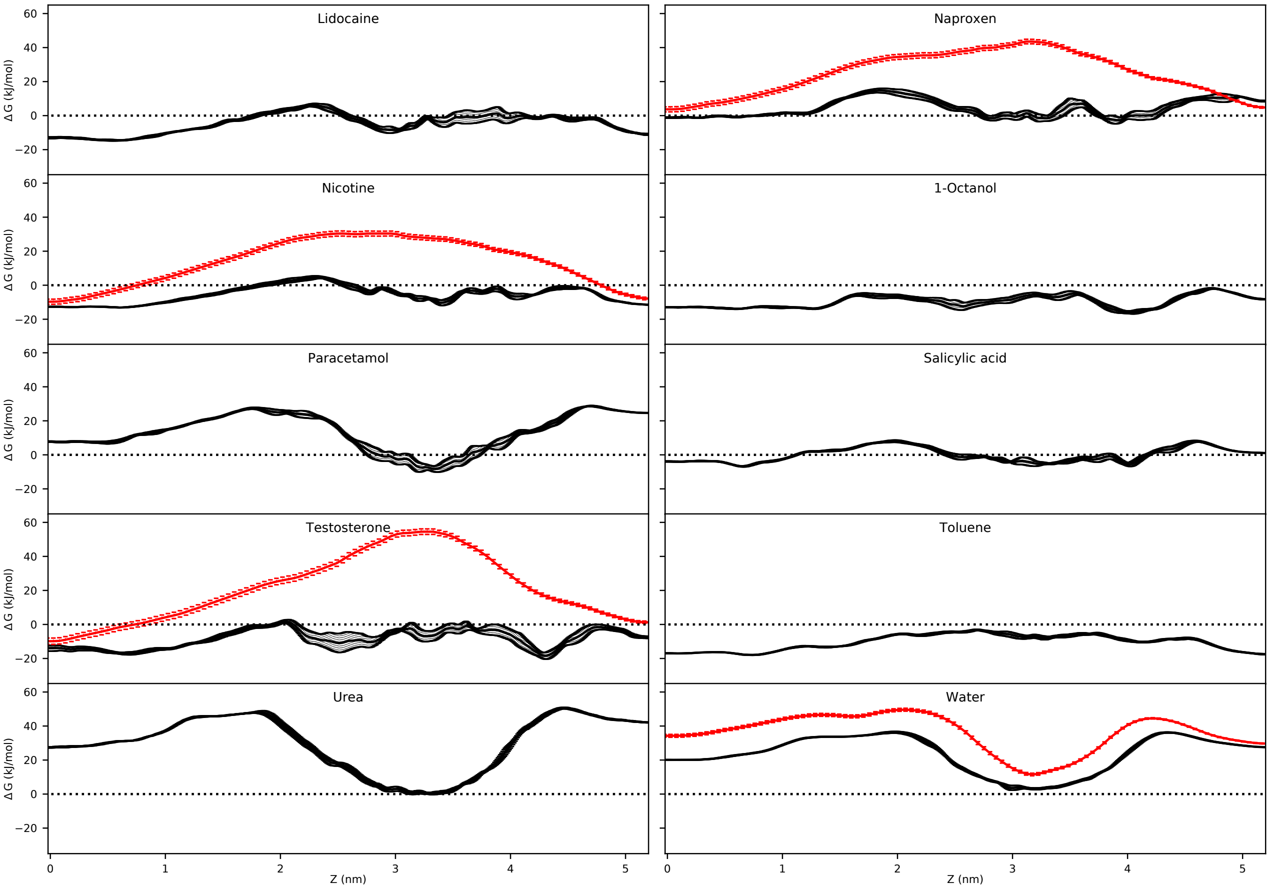

Figure S5: PMFs of 20 permeants. The PMFs are calibrated relative to the hydration free energies of the compounds. The profiles in black show the results from this study, whereas the profiles in red are from our previous FR pulling simulations (pulling at 0.2 nm ns^{-1}) (2). The curves in red were only calibrated based on the free energy difference, to the water solvent, at the ceramide sphingoid chain interface region (at ~5.2 nm). When calculating the permeability coefficients, the PMFs (in black) were shifted (up) so that their minima were ≥0 kJ mol^{-1}. I.e., the zero point was set to the lowest of the hydration free energy and the lowest point in the PMF. *Continued from previous page.*

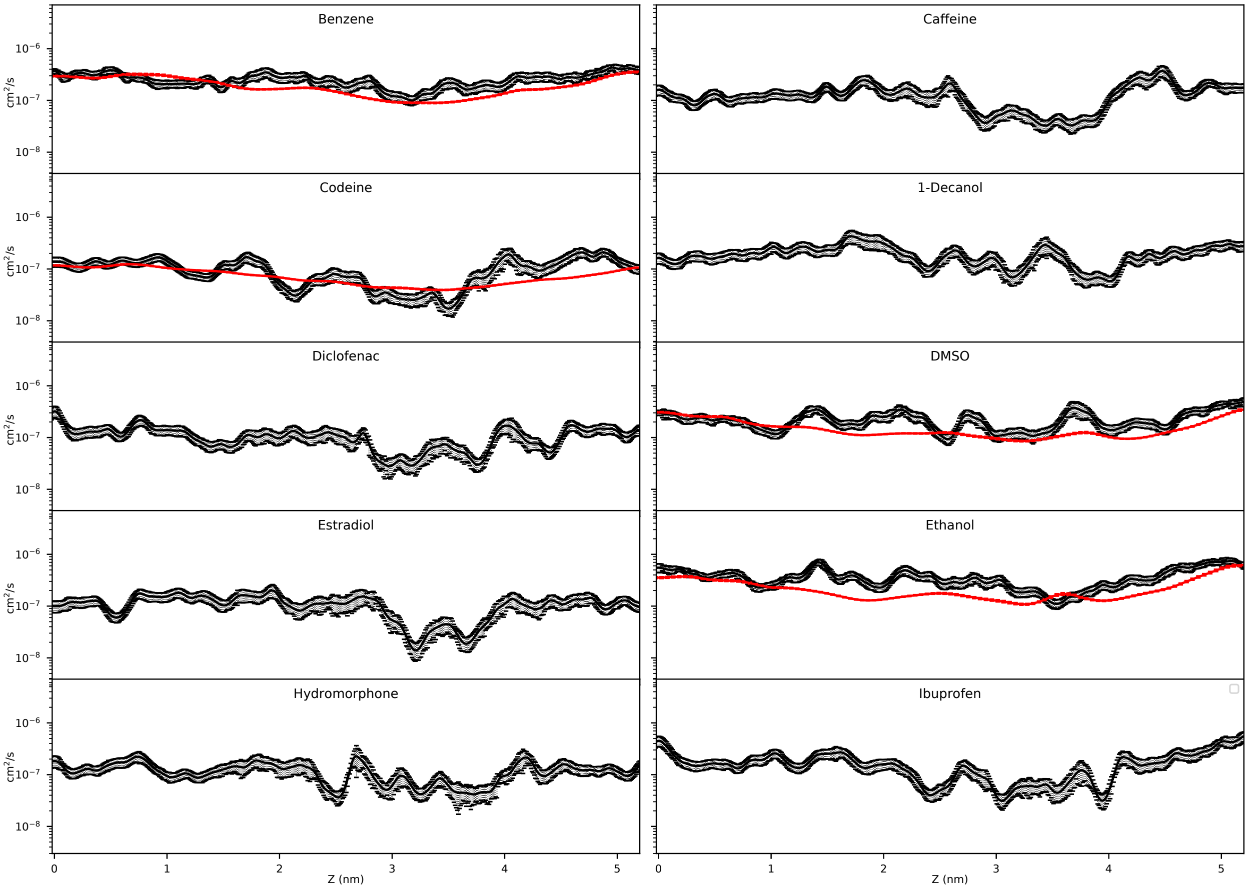

Figure S6: Diffusion coefficients of 20 permeants. The profiles in black show the results from this study, whereas the profiles in red are from our previous FR pulling simulations (pulling at $0.2\,\mathrm{nm\,ns^{-1}}$) (2). The diffusion profiles from AWH were smoothened using a running median filter of 0.2 nm width, whereas a Hanning filter of 11 point (0.5 nm) width was used for the diffusion profiles from FR pulling. *Continued on next page.*

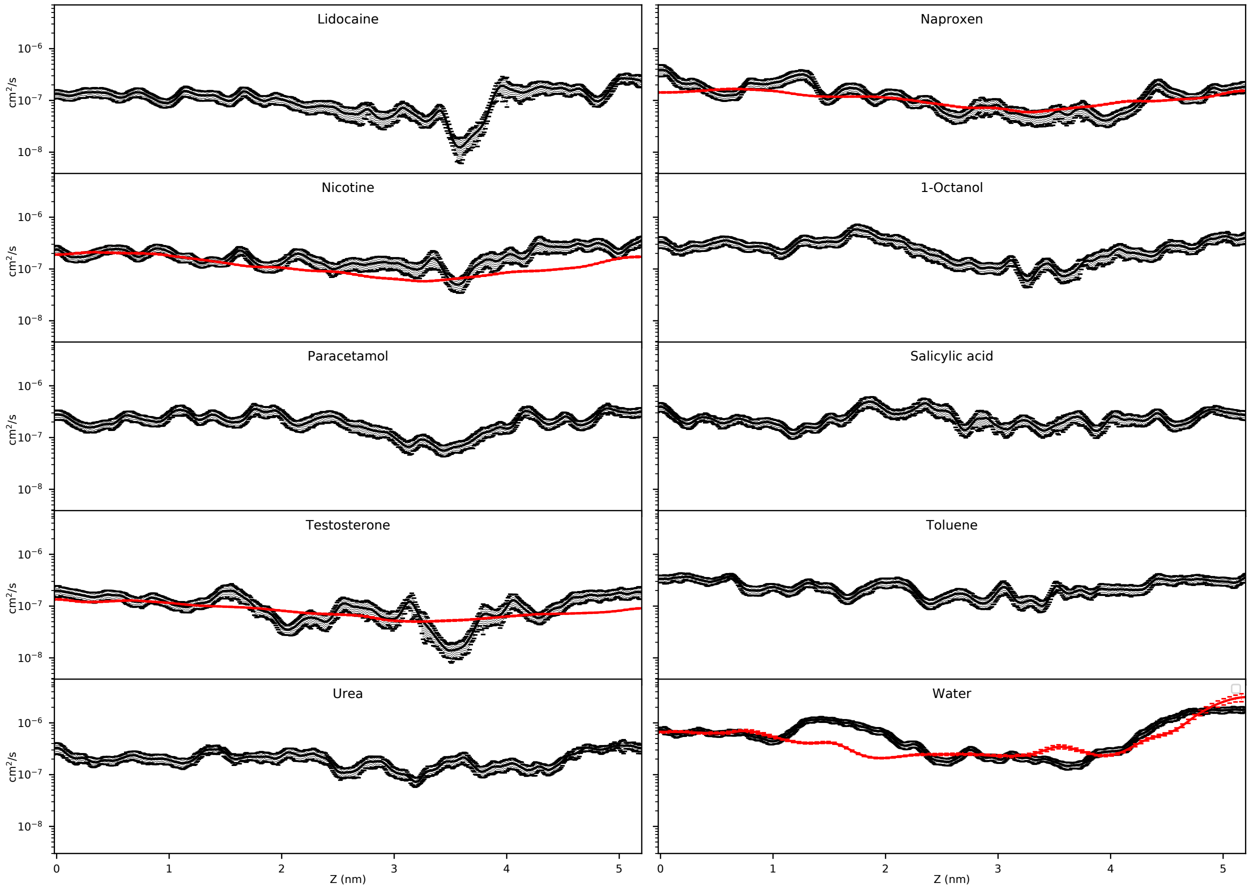

Figure S6: Diffusion coefficients of 20 permeants. The profiles in black show the results from this study, whereas the profiles in red are from our previous FR pulling simulations (pulling at 0.2 nm ns^{-1}) (2). The diffusion profiles from AWH were smoothened using a running median filter of 0.2 nm width, whereas a Hanning filter of 11 point (0.5 nm) width was used for the diffusion profiles from FR pulling. *Continued from previous page.*

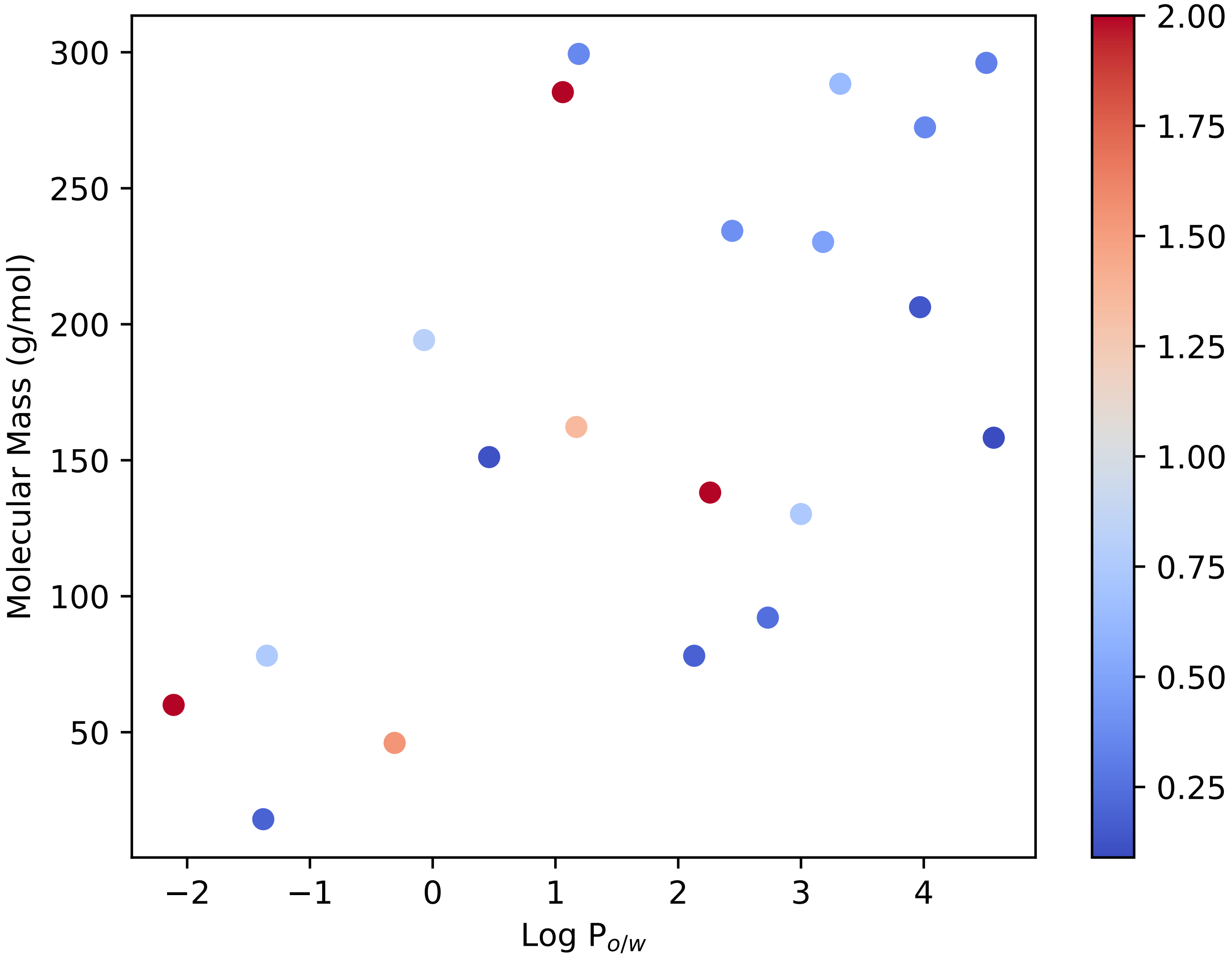

Figure S7: Molecular mass and log $P_{o/w}$ of permeants as well as their log K_P square difference (cm^2h^{-2}), between experimental data and calculation results, shown by the color scale.

Table S1: Permeability coefficients of unionized species at 32 °C, with corrections for temperature differences according to Abraham and Martins (3). If only observations from ionized species were found, corrections for pH/pK_a were then obtained from Abraham and Martins (3). The log $K_{P_{exp}}$ uncertainty is the standard error of the mean (SEM) of the measurements, if there were at least three reported measurements available. If there were only two experimental measurements the SEM was assumed to be 0.3 and shown in italics and if there was only one experimental measurement the SEM was reported as 0.4 (in italics). The calculated results are from 5 independent sets of simulations, each running 24 communicating AWH walkers for 450 ns.

**Permeant**	**log $K_{P_{exp}}$** (cm/h)	**log $K_{P_{calc}}$** (cm/h)
Benzene	−0.7 ± *0.3*[a]	−0.3 ± 0.1
Caffeine	−3.5 ± 0.2[b]	−2.6 ± 0.2
Codeine	−3.8 ± *0.4*[c]	−3.2 ± 0.4
1-Decanol	−0.8 ± *0.4*[d]	−0.5 ± 0.5
Diclofenac	−1.9 ± *0.4*[e]	−2.5 ± 0.3
DMSO	−3.1 ± *0.4*[f]	−4.0 ± 0.1
Estradiol	−2.4 ± 0.2[g]	−1.8 ± 0.4
Ethanol	−2.8 ± *0.3*[h]	−1.6 ± 0.1
Hydromorphone	−4.4 ± *0.4*[i]	−2.6 ± 0.3
Ibuprofen	−1.6 ± *0.4*[j]	−1.2 ± 0.1
Lidocaine	−2.2 ± *0.4*[k]	−1.6 ± 0.2
Naproxen	−1.6 ± *0.4*[l]	−0.9 ± 0.4
Nicotine	−2.0 ± *0.3*[m]	−0.8 ± 0.1
1-Octanol	−1.0 ± *0.4*[n]	−0.1 ± 0.2
Paracetamol	−4.3 ± *0.4*[o]	−4.0 ± 0.3
Salicylic acid	−1.7 ± *0.3*[p]	−0.3 ± 0.1
Testosterone	−2.5 ± 0.3[q]	−1.7 ± 0.4
Toluene	0.0 ± *0.4*[r]	−0.5 ± 0.1
Urea	−4.1 ± *0.3*[s]	−6.2 ± 0.1
Water	−3.1 ± 0.2[t]	−3.5 ± 0.1
Mean diff.		0.38
Mean abs. diff.		0.84
Mean sq. diff.		0.93[u]

[a] log $K_{P_{exp}}$ −0.80 cm h^{-1} at 25 °C (4) and −0.95 cm h^{-1} at 31 °C (5).
[b] log $K_{P_{exp}}$ −4.00 cm h^{-1} at 25 °C (4), −3.59 cm h^{-1} at 32 °C (6) and −3.19 cm h^{-1} at 32 °C (7).
[c] log $K_{P_{exp}}$ −4.31 cm h^{-1} at 37 °C and pH 7.4 (8) (pK_a 8.2).
[d] log $K_{P_{exp}}$ −1.10 cm h^{-1} at 25 °C (9).
[e] log $K_{P_{exp}}$ −1.74 cm h^{-1} at 37 °C, unionized (10).
[f] log $K_{P_{exp}}$ −3.10 cm h^{-1} at 5 % conc. at unspecified temperature (11). Cf., at 40 % conc. the log $K_{P_{exp}}$ was −1.80 cm h^{-1}.
[g] log $K_{P_{exp}}$ −2.01 cm h^{-1} at 37 °C (12), −2.28 cm h^{-1} at 30 °C (4), −2.34 cm h^{-1} at 34 °C (SC temperature) (13), −2.37 cm h^{-1} at 32 °C (14), −2.41 cm h^{-1} through full skin at 30 °C (15), −2.49 cm h^{-1} at 25 °C (4) and −3.52 cm h^{-1} at 26 °C (16).
[h] log $K_{P_{exp}}$ −3.14 cm h^{-1} at 25 °C (9) and −3.00 cm h^{-1} at 25 °C (17).
[i] log $K_{P_{exp}}$ −4.82 cm h^{-1} at 37 °C, pH 7.4 (8) (pK_a 8.2).
[j] log $K_{P_{exp}}$ −1.44 cm h^{-1} at 37 °C, presumably unionized (18).
[k] log $K_{P_{exp}}$ −2.15 cm h^{-1} at 32 °C, pH 10.0 (8) (pK_a 8.0).
[l] log $K_{P_{exp}}$ −1.42 cm h^{-1} at 37 °C, unionized (10).
[m] log $K_{P_{exp}}$ −1.71 cm h^{-1} at unknown temperature, unionized (19), −2.08 cm h^{-1} at 37 °C, unionized (20) .
[n] log $K_{P_{exp}}$ −1.28 cm h^{-1} at 25 °C (9).
[o] log $K_{P_{exp}}$ −4.30 cm h^{-1} at 32 °C (21).
[p] log $K_{P_{exp}}$ −1.52 cm h^{-1} at 37 °C, unionized (10) and −1.58 cm h^{-1} at 32 °C, pH 2.5 (22) (pK_a 3.0).
[q] log $K_{P_{exp}}$ −2.66 cm h^{-1} at 25 °C (4), −3.40 cm h^{-1} at 26 °C (16), −1.85 cm h^{-1} at 32 °C (23) and −3.07 cm h^{-1} at 25 °C (24).
[r] log $K_{P_{exp}}$ 0.0 cm h^{-1} at unknown temperature (25).
[s] log $K_{P_{exp}}$ −3.89 cm h^{-1} at 37 °C (26) and −4.04 cm h^{-1} at 34 °C (SC temperature) (13).
[t] log $K_{P_{exp}}$ −3.30 cm h^{-1} at 25 °C (9), −3.70 cm h^{-1} at 25 °C (9), −3.00 cm h^{-1} at 25 °C (17) and −3.40 cm h^{-1} at unspecified temperature (11).
[u] $cm^2\ h^{-2}$

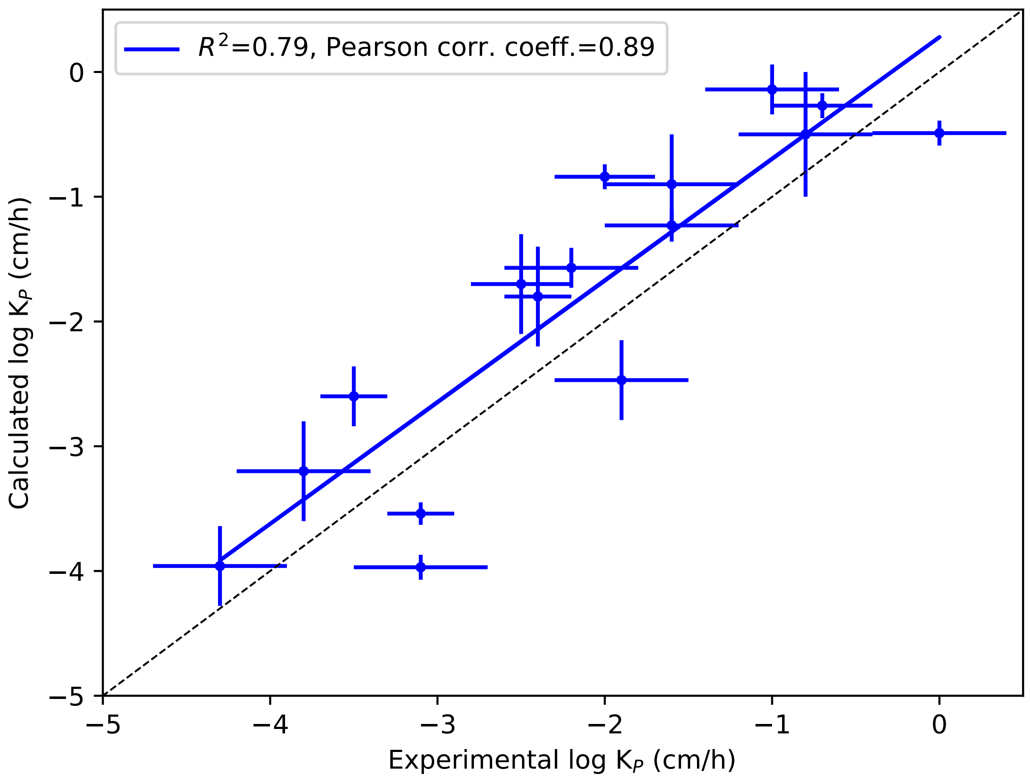

Figure S8: The relationship between experimental log K_P and those calculated from MD simulations, with the two largest outliers (urea and hydromorphone) excluded. The blue line shows the linear regression, whereas the dashed black line is the identity line. The error bars represent 1 SEM (standard error of the mean), which is approximated for experimental values. Details about the approximation is presented in the header of Table S1.

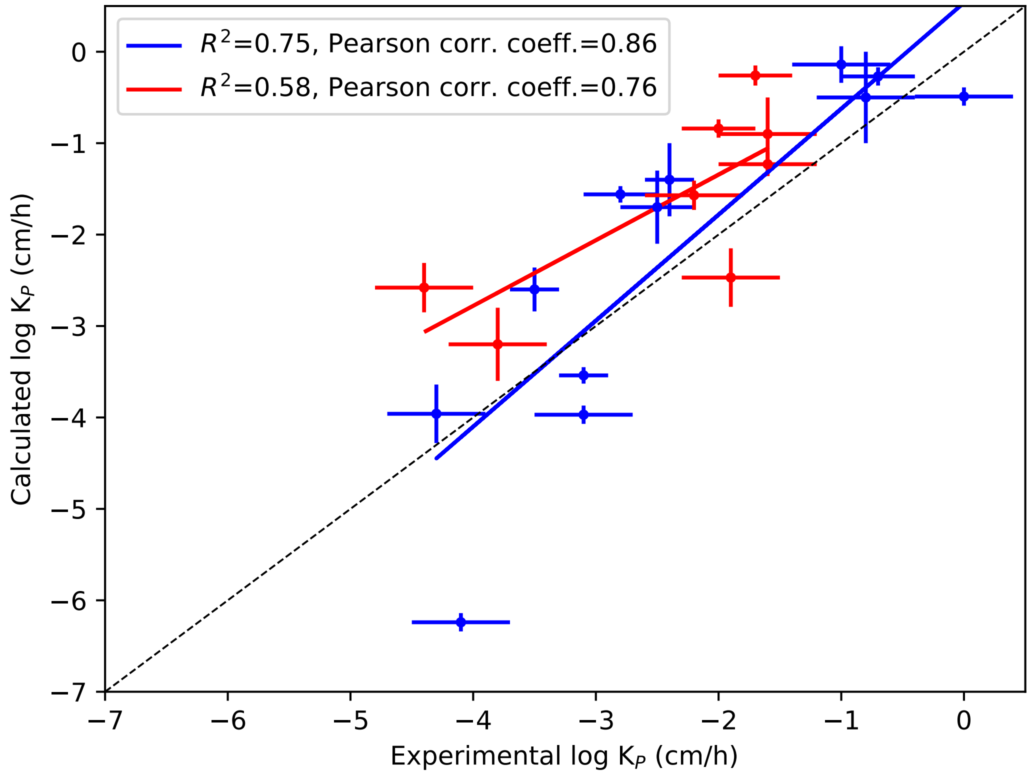

Figure S9: Separating molecules that are not ionizable (shown in blue) under the *ex vivo/in vitro* conditions from those that are ionizable (shown in red). The ionizable compounds are codeine, diclofenac, hydromorphone, ibuprofen, lidocaine, naproxen, nicotine and salicylic acid.

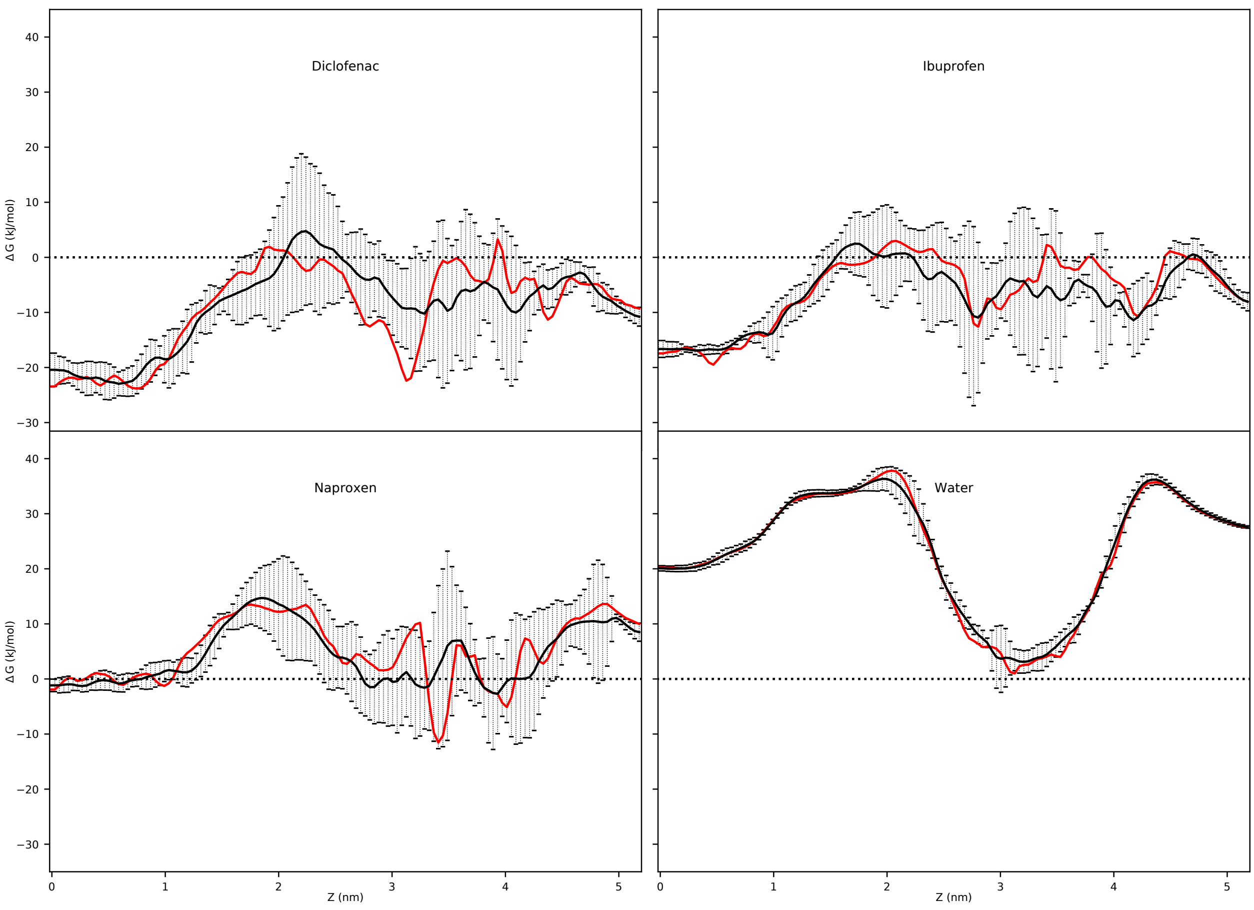

Figure S10: Comparing PMFs from simulations with 3 fs and 2 fs MD time steps. The black curves show the results from the five sets of simulations run with heavy hydrogen atoms and a 3 fs MD time step (cf. Fig.S5). The uncertainties here represent 2 STDs in order to allow a quick comparison to the red curves. The red curves show the results from one set of AWH simulations run with normal hydrogen mass and a 2 fs MD time step. Only every 4th data point is plotted in order to improve the clarity.

S2.4 Evaluating a 3 fs MD Time Step

The reported results are from simulations with a 3 fs MD iteration time step. That required increasing the mass of hydrogen atoms, by repartitioning mass from their bound heavy atom. In order to verify that this did not affect the results the PMFs and local diffusion coefficients of four molecules were calculated using normal hydrogen mass and a standard 2 fs MD iteration time step. The comparison is shown in Fig. S10 and S11. No significant differences were observed between the two alternative simulation parameter settings.

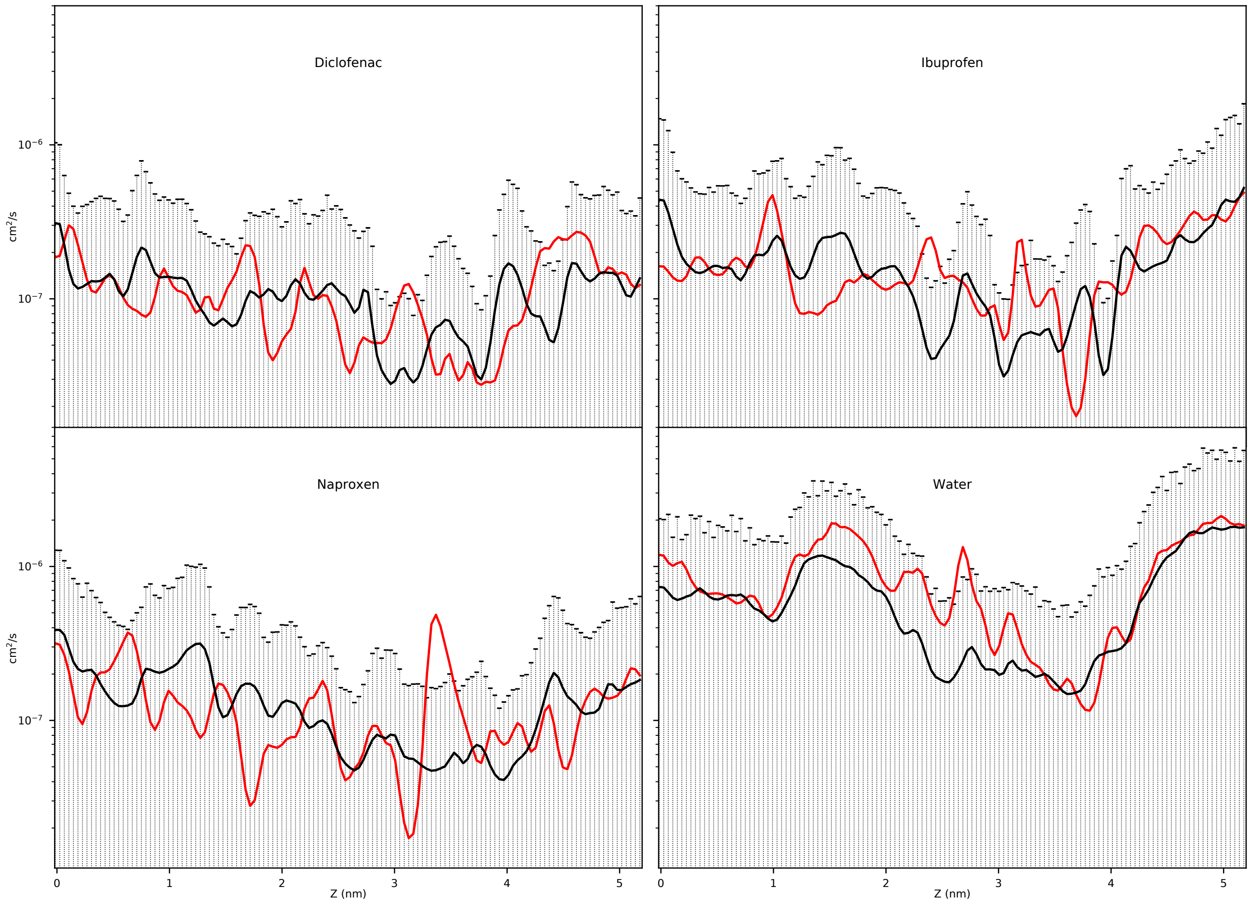

Figure S11: Comparing diffusion coefficients from simulations with 3 fs and 2 fs MD time steps. The black curves show the results from the five sets of simulations run with heavy hydrogen atoms and a 3 fs MD time step (cf. Fig.S6). The uncertainties here represent 2 STDs in order to allow a quick comparison to the red curves. The red curves show the results from one set of AWH simulations run with normal hydrogen mass and a 2 fs MD time step. Only every 4th data point is plotted in order to improve the clarity.

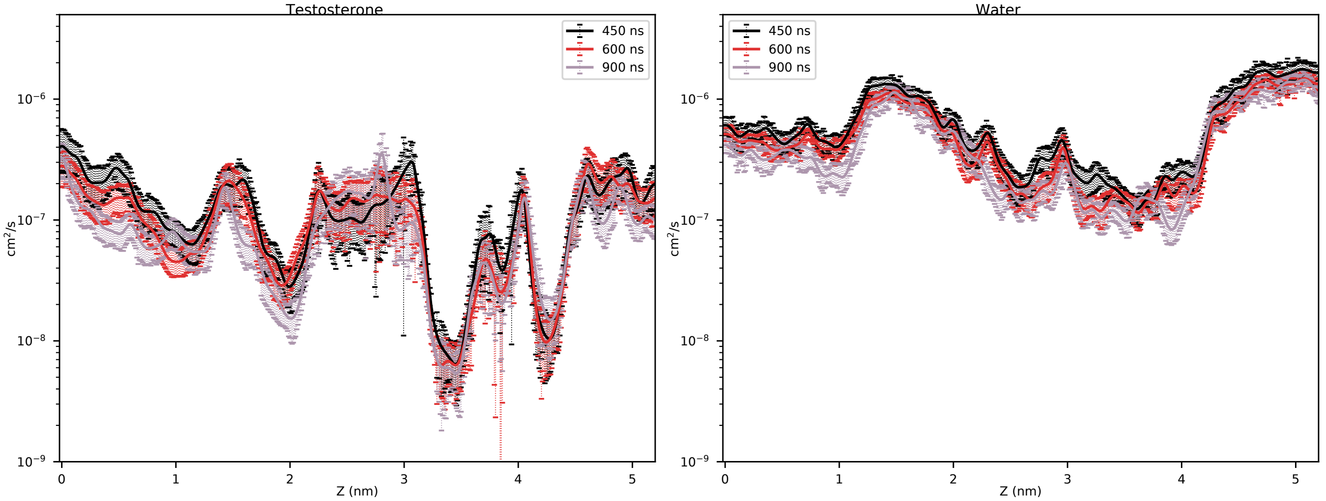

Figure S12: Calculated diffusion coefficients of testosterone and water from extended AWH simulations (24 communicating AWH walkers). The average diffusion coefficients through the system for testosterone were 1.3×10^{-7} cm^2 s^{-1}, 1.1×10^{-7} cm^2 s^{-1} and 8.8×10^{-8} cm^2 s^{-1} at 450 ns, 600 ns and 900 ns, respectively. For water they were 6.7×10^{-7} cm^2 s^{-1}, 5.5×10^{-7} cm^2 s^{-1} and 4.9×10^{-7} cm^2 s^{-1} at the same time points.

Table S2: Permeability coefficients of testosterone and water from extended AWH simulations. The AWH uncertainties from one single set of simulations (24 communicating AWH walkers) are rough estimates and are expected to be too low.

	**Simulation time (ns) per AWH walker (24 walkers)**		
**Permeant**	**450**	**600**	**900**
Testosterone	−2.9 ± 0.4	−2.8 ± 0.3	−2.8 ± 0.3
Water	−3.5 ± 0.2	−3.5 ± 0.2	−3.5 ± 0.2

S2.5 Convergence of Diffusion Coefficients

Fig. S12 shows how the calculated diffusion coefficients change with extended simulation times. Especially for testosterone it can be seen that longer simulation times result in lower diffusion coefficients. Table S2 shows the calculated permeability coefficients from the same simulations. Similar trends have been observed when lowering the pulling speed in FR simulations or increasing the sampling time in umbrella sampling simulations. A comparison of water diffusion coefficients from methods is shown in Fig. S13. The umbrella sampling results are from the same set of simulations, but the US 145-150 ns results discarded the first 145 ns from each simulation.

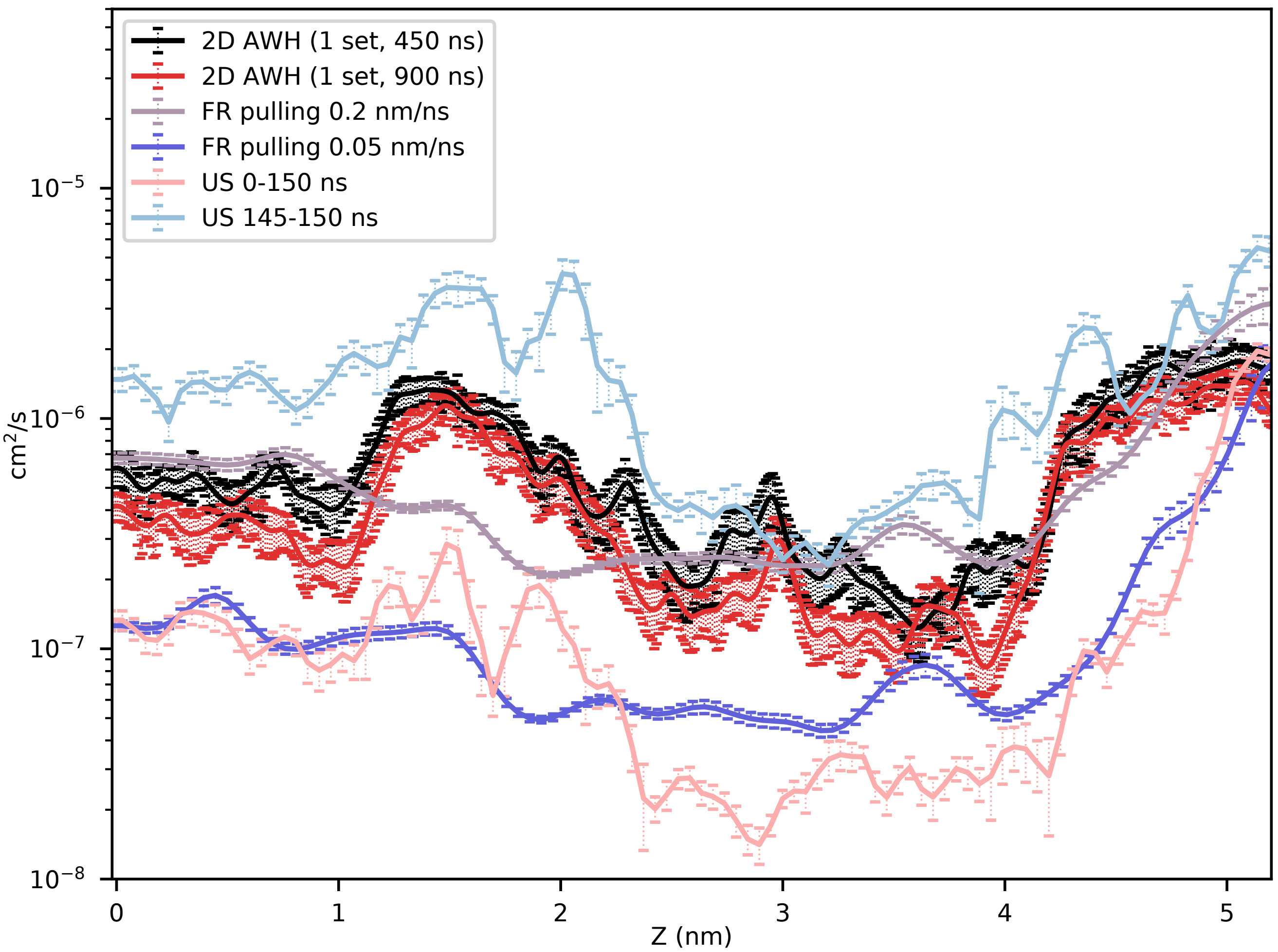

Figure S13: Calculated diffusion coefficients of water using 2D AWH, FR pulling and umbrella sampling. Longer sampling, or slower pulling, results in lower diffusion coefficients through the gel-like skin barrier structure. The AWH simulations results are from 1 set of 24 communicating AWH walkers, each running 450 or 900 ns for 11 and 22 µs, respectively. The 0.2 nm/ns FR pulling output is from 50 simulations pulling two molecules in each direction, for a total of 5 µs. The 0.05 nm/ns FR data is from 60 simulations pulling two molecules halfway across the system in each direction, for a total of 12 µs. The full set of umbrella sampling simulations is from 120 simulations, each with 4 water molecules, for a total of 18 µs for the full 0-150 ns data. The US 145-150 ns set only analyzed the last 5 ns from the same simulations. The diffusion profiles from AWH were smoothened using a running median filter of 0.2 nm width. A Hanning filter of 0.5 nm width was used for the diffusion profiles from FR pulling and 0.2 nm for umbrella sampling.

REFERENCES

1. Nategholeslam, M., C. G. Gray, and B. Tomberli, 2017. Stiff Spring Approximation Revisited: Inertial Effects in Nonequilibrium Trajectories. *J. Phys. Chem. B* 121:391–403. http://pubs.acs.org/doi/abs/10.1021/acs.jpcb.6b08701.

2. Lundborg, M., C. L. Wennberg, A. Narangifard, E. Lindahl, and L. Norlén, 2018. Predicting drug permeability through skin using molecular dynamics simulation. *J. Control. Release* 283:269–279. http://www.sciencedirect.com/science/article/pii/S016836591830292X.

3. Abraham, M. H., and F. Martins, 2004. Human skin permeation and partition: General linear free-energy relationship analyses. *J. Pharm. Sci.* 93:1508–1523. http://onlinelibrary.wiley.com/doi/10.1002/jps.20070/abstract.

4. Mitragotri, S., D. A. Edwards, D. Blankschtein, and R. Langer, 1995. A mechanistic study of ultrasonically-enhanced transdermal drug delivery. *J. Pharm. Sci.* 84:697–706. http://onlinelibrary.wiley.com/doi/10.1002/jps.2600840607/abstract.

5. Blank, I. H., and D. J. McAuliffe, 1985. Penetration of Benzene Through Human Skin. *J. Invest. Dermatol.* 85:522–526. http://www.sciencedirect.com/science/article/pii/S0022202X15438461.

6. Dias, M., A. Farinha, E. Faustino, J. Hadgraft, J. Pais, and C. Toscano, 1999. Topical delivery of caffeine from some commercial formulations. *Int. J. Pharm.* 182:41–47. http://www.sciencedirect.com/science/article/pii/S0378517399000678.

7. Uchida, T., W. R. Kadhum, S. Kanai, H. Todo, T. Oshizaka, and K. Sugibayashi, 2015. Prediction of skin permeation by chemical compounds using the artificial membrane, Strat-M™. *Eur. J. Pharm. Sci.* 67:113–118. http://www.sciencedirect.com/science/article/pii/S0928098714004163.

8. Roy, S. D., and G. L. Flynn, 1989. Transdermal Delivery of Narcotic Analgesics: Comparative Permeabilities of Narcotic Analgesics Through Human Cadaver Skin. *Pharm. Res.* 6:825–832. http://link.springer.com/article/10.1023/A:1015944018555.

9. Scheuplein, R. J., and I. H. Blank, 1973. Mechanism of Percutaneous Absorption. Iv. Penetration of Nonelectrolytes (alcohols) from Aqueous Solutions and from Pure Liquids. *J. Invest. Dermatol.* 60:286–296. http://www.nature.com.ezp.sub.su.se/jid/journal/v60/n5/abs/5617718a.html.

10. Singh, P., and M. S. Roberts, 1994. Skin permeability and local tissue concentrations of nonsteroidal anti-inflammatory drugs after topical application. *J. Pharmacol. Exp. Ther.* 268:144–151. https://jpet.aspetjournals.org/content/268/1/144.

11. Daugherty, J. E., 1997. Fundamentals of Receptor Assessment. *In* Assessment of Chemical Exposures: Calculation Methods for Environmental Professionals, CRC Press, 309.

12. Liu, P., W. I. Higuchi, A.-H. Ghanem, and W. R. Good, 1994. Transport of beta-Estradiol in Freshly Excised Human Skin in Vitro: Diffusion and Metabolism in Each Skin Layer. *Pharm. Res.* 11:1777–1784. https://doi.org/10.1023/A:1018975602818.

13. Intarakumhaeng, R., A. Wanasathop, and S. K. Li, 2018. Effects of solvents on skin absorption of nonvolatile lipophilic and polar solutes under finite dose conditions. *International Journal of Pharmaceutics* 536:405–413. https://linkinghub.elsevier.com/retrieve/pii/S0378517317310931.

14. Megrab, N. A., A. C. Williams, and B. W. Barry, 1995. Oestradiol permeation across human skin, silastic and snake skin membranes: The effects of ethanol/water co-solvent systems. *Int. J. Pharm.* 116:101–112. http://www.sciencedirect.com/science/article/pii/037851739400321U.

15. Galey, W. R., H. K. Lonsdale, and S. Nacht, 1976. The In Vitro Permeability Of Skin And Buccal Mucosa To Selected Drugs And Tritiated Water. *J. Invest. Dermatol.* 67:713–717. http://www.sciencedirect.com/science/article/pii/S0022202X15449334.

16. Scheuplein, R. J., I. H. Blank, G. J. Brauner, and D. J. Macfarlane, 1969. Percutaneous Absorption of Steroids. *J. Invest. Dermatol.* 52:63–70. http://www.sciencedirect.com/science/article/pii/S0022202X15476195.

17. Scheuplein, R. J., 1965. Mechanism of Percutaneous Adsorption. *J. Invest. Dermatol.* 45:334–346. http://www.sciencedirect.com/science/article/pii/S0022202X15470058.

18. Degim, I. T., W. J. Pugh, and J. Hadgraft, 1998. Skin permeability data: anomalous results. *Int. J. Pharm.* 170:129–133. http://www.sciencedirect.com/science/article/pii/S0378517398001136.

19. Flynn, G. L., 1990. Physicochemical determinants of skin absorption. *In* T. R. Gerrity, and C. J. Henry, editors, Principles of Route-to-route Extrapolation for Risk Assessment, Elsevier, New York, 93–127.

20. Zorin, S., F. Kuylenstierna, and H. Thulin, 1999. In vitro test of nicotine's permeability through human skin. Risk evaluation and safety aspects. *Ann. Occup. Hyg.* 43:405–413. https://doi.org/10.1093/annhyg/43.6.405.

21. Priprem, A., C. Khamlert, T. Pongjanyakul, S. Radapong, T. Rittirod, and P. Chitropas, 2008. Comparative Permeation Studies between Scale Region of Shed Snake Skin and Human Skin In vitro. *Am. J. Agricult. Biol. Sci.* https://agris.fao.org/agris-search/search.do?recordID=DJ2012079471.

22. Qvist, M. H., U. Hoeck, B. Kreilgaard, F. Madsen, and S. Frokjaer, 2000. Evaluation of Göttingen minipig skin for transdermal in vitro permeation studies. *European Journal of Pharmaceutical Sciences* 11:59–68. http://www.sciencedirect.com/science/article/pii/S0928098700000919.

23. An-eX Analytical Services Ltd, 2017. Assessment of the permeation of testosterone through human skin in vitro from saturated aqueous solution. Technical Report E05/02/17.

24. Siddiqui, O., M. S. Roberts, and A. E. Polack, 1989. Percutaneous absorption of steroids: relative contributions of epidermal penetration and dermal clearance. *J. Pharmacokinet. Biopharm.* 17:405–424.

25. Dutkiewicz, T., and H. Tyras, 1968. Skin absorption of toluene, styrene, and xylene by man. *Br. J. Ind. Med.* 25:243. https://www.ncbi.nlm.nih.gov/pmc/articles/PMC1008781/.

26. Peck, K. D., A. Ghanem, and W. I. Higuchi, 1994. Hindered Diffusion of Polar Molecules Through and Effective Pore Radii Estimates of Intact and Ethanol Treated Human Epidermal Membrane. *Pharm. Res.* 11:1306–1314. http://link.springer.com/10.1023/A:1018998529283.
